# Technology-Based Substance Use Interventions for Emerging Adults and College Students: A Systematic Review and Meta-Analysis

**DOI:** 10.1007/s11469-024-01433-7

**Published:** 2024-12-26

**Authors:** Audrey Hang Hai, Laura Curran, Jocelyn N. Simons, Kate B. Carey, Patrick S. Bordnick

**Affiliations:** 1 School of Social Work, Tulane University, 127 Elk Place, New Orleans, LA 70112 USA; 2 College of Behavioral and Community Sciences, Department of Mental Health Law and Policy, University of South Florida- Sarasota-Manatee, 8350 N Tamiami Trail, Sarasota, FL 34243 USA; 3 Department of Speech Pathology, Xavier University of Louisiana, 1 Drexel Drive, New Orleans, LA 70125 USA; 4 Center for Alcohol and Addiction Studies, Brown University, 121 South Main Street, Providence, Rhode Island 02903 USA; 5 Department of Behavioral and Social Sciences, Brown School of Public Health, Providence, 121 South Main Street, Providence, Rhode Island 02903 USA

**Keywords:** Technology-based interventions, Alcohol, Drug use, Emerging adults, College students, Meta-analysis

## Abstract

**Objective:**

To synthesize randomized controlled trial evidence on technology-based interventions’ (TBIs) effectiveness for substance use among emerging adults (EA)/college students (CS).

**Methods:**

Nine electronic databases were searched. Two reviewers independently screened studies, extracted data, and assessed evidence quality. We used robust variance estimation in meta-regression for effect size synthesis and moderator analysis.

**Results:**

Based on 130 studies, the overall between-group effect size was 0.23 (95% CI = 0.18, 0.28). The effect sizes for comparing TBIs with no treatment, standard care, and non-technology interventions were 0.25 (CI = 0.19, 0.31), 0.23 (CI = 0.15, 0.32), and 0.12 (CI = -0.02, 0.25), respectively. Older participants showed significantly larger effect sizes, and interventions using multiple technologies had larger effects than smartphone-based ones.

**Conclusion:**

TBIs are effective in reducing substance use in EA/CS, with outcomes comparable to non-technology interventions and advantages over no treatment and standard care. Future research should address drug-related outcomes, multi-technology approaches, age-appropriate designs, and cultural diversity.

**Supplementary Information:**

The online version contains supplementary material available at 10.1007/s11469-024-01433-7.

Substance use represents a major global public health concern, and poses significant societal and individual burdens, particularly among emerging adults (ages 18–24) and the subset of emerging adults that are college students. The trajectory of substance use typically initiates in early adolescence, peaks in emerging adulthood, and subsequently declines over time. This pattern has been found consistent across diverse cultures (Gopiram & Kishore, 2014). For example, in the United States (US), 1 in 3 emerging adults reported binge drinking and nearly 2 in 5 emerging adults used illicit drugs (SAMHSA, 2023). Emerging adults also have the highest prevalence of substance use disorder (SUD) (SAMHSA, 2023). Emerging adult substance use is also notable in other developed and developing countries, with past-month binge drinking prevalence rates of 50.7% in Germany, 45.4% in Chile, and 18.2% in South Korea, and past-year cannabis use rates of 32.9% in Chile, 26.6% in Spain, and 24.8% in New Zealand (Degenhardt et al., 2016). Furthermore, research suggests that the prevalence of substance use might be comparable or even higher among college students compared to non-college-attending emerging adults (Skidmore et al., 2016).

Substance use among emerging adults/college students poses a myriad of consequences that impact various facets of their lives. Academic and occupational challenges manifest as poor academic performance, absenteeism, and reduced workplace productivity (Allen et al., 2018; Benson et al., 2015; Sahker et al., 2019). Legal problems, such as arrests for offenses like driving under the influence, strained social relationships, financial struggles, and an elevated risk of accidents and injuries are also common consequences (CDC, 2024; Mundt et al., 2012; Tambling et al., 2022). Substance use may also lead to sexual risk-taking, mental health disorders, the development of substance use disorders, cognitive impairment, and, in severe cases, overdose and death (Caldeira et al., 2009; Kenne et al., 2017; SAMHSA & Office of the Surgeon General (US), 2016) Long-term health-related issues include an increased risk of liver disease, respiratory problems, cardiovascular issues, and cancer (SAMHSA & Office of the Surgeon General (US), 2016). In light of the high prevalence and significant consequences of substance use among emerging adults and college students, timely intervention is crucial to reduce harms accruing to individuals and their communities. However, the majority of emerging adults/college students dealing with substance use issues do not receive assistance, with 9 out of 10 emerging adults facing SUD going untreated (SAMHSA, 2024). This underscores the pressing need for effective and accessible interventions for this population.

Technology-based interventions (TBIs) (i.e., interventions delivered by technologies such as computers, websites, and mobile apps) offer innovative solutions to address the global public health concern associated with substance use among emerging adults/college students. The widespread adoption of technology among emerging adults/college students is evident globally. For example, as of 2021, emerging adults constitute a significant 23% of online users worldwide (Statista, 2023). Smartphone ownership is nearly ubiquitous among emerging adults in developed countries, ranging from 84% in Argentina to 99% in South Korea and the Netherlands (Pew Research Center, 2019). Even in developing countries, smartphone ownership rates are substantial, ranging from 37% in India to 85% in Brazil (Pew Research Center, 2019).

By leveraging technology, interventions can engage emerging adults/college students on platforms they are already familiar with and frequent regularly, enhancing overall engagement. TBIs can also extend support beyond clinical settings and provide timely support precisely when emerging adults/college students need it the most. Importantly, TBIs can be easily scaled to reach a greater number of emerging adults/college students in need and thus potentially make a more significant public health impact. In addition, TBIs offer a less stigmatizing alternative for those grappling with substance use by allowing discreet access to support. TBIs can also maximize intervention fidelity and may lower costs compared to traditional in-person interventions. Therefore, technology presents a highly promising avenue for disseminating substance use interventions to emerging adults/college students.

A rapidly expanding body of research has examined the effectiveness of TBIs for substance use, with several systematic reviews and meta-analyses consolidating studies on various types of TBIs for substance use among adolescents or adults. Reviews specific to young people (adolescents and emerging adults/college students) generally demonstrated promising benefits of TBIs in reducing alcohol and other drug use, with the predominant focus of the existing research being on alcohol use and TBIs that employ the personalized or normative feedback approach and web-based technology (Champion et al., 2019; Kazemi et al., 2021; Monarque et al., 2023; Ondersma et al., 2019). For example, Hutton et al.’s (2020) review supported the acceptability and effectiveness of TBIs (particularly text-messaging TBIs) as early intervention/prevention for reducing alcohol consumption among adolescents and emerging adults (Hutton et al., 2020). According to the meta-analysis by Tait and Christensen (2010), online interventions showed a small yet statistically significant effect on reducing substance use among adolescents and emerging adults facing problematic substance use, with an overall effect size of d = − 0.22 (95% CI, − 0.34 to − 0.10). Specifically, online interventions were effective for reducing alcohol consumption level (d = − 0.12, 95% CI = − 0.22 to − 0.02), frequency of binge or heavy drinking (d = − 0.35, 95% CI = − 0.64 to − 0.06), and alcohol-related social issues (d = − 0.57, 95% CI = − 0.98 to − 0.15), but not for preventing the development of alcohol-related problems among individuals who did not drink at baseline (Tait & Christensen, 2010). Additionally, O'Logbon (2023) conducted a systematic review (*N* = 42) and meta-analysis (*N* = 18), revealing small but statistically significant reductions in weekly alcohol consumption through TBIs (compared to controls) for youth and emerging adults with problematic substance use or SUD (effect size d = − 0.12, 95% CI = − 0.17 to − 0.06) (O’Logbon et al., 2023).

Similarly, reviews on TBIs for substance use among college students showed promising efficacy of TBIs for alcohol use/misuse and harm reduction and noted a lack of TBI research on college student drug use (effect sizes ranged from −0.01 to 0.38) (Berman et al., 2016; K. B. Carey et al., 2009, 2012; Dick et al., 2019; Elliott et al., 2008; Gulliver et al., 2015; Leeman et al., 2015). For example, Carey et al. (2009) found that computer-delivered interventions to reduce college student drinking demonstrated small but significant effects, with effect sizes ranging from d = 0.10 to d = 0.16 for drinking outcomes. In a later meta-analysis, Carey et al. (2012) reported that computer-delivered intervention participants showed reductions in drinking quantity, frequency, and peak intoxication at short-term follow-up (effect sizes d = 0.13 to d = 0.29), though these effects were not sustained over time. Gulliver et al. (2015) reviewed TBI for tobacco and other drug use in college students, reporting post-intervention effect sizes of d = 0.38 and d = − 0.01 for cannabis use interventions, and d = 0.28 for multi-targeted interventions addressing tobacco, alcohol, and cannabis (cannabis use intentions d = 0.27).

Several reviews also have examined TBIs for substance use among adults and generally supported TBIs’ effectiveness, with effect sizes ranging from 0.19 to 0.30. Kiluk et al. (2019) specifically analyzed technology-delivered cognitive-behavioral therapy (CBT) interventions for alcohol use, finding that CBT TBI, as a stand-alone treatment compared to minimal treatment control, demonstrated a small but significant effect (d = 0.20, 95% CI = 0.22–0.38). Additionally, when CBT TBI was combined with treatment as usual, it produced a stable, moderate effect (d = 0.30, 95% CI = 0.10–0.50) over a 12-month follow-up period (Kiluk et al., 2019). Riper et al.’s (2018) individual patient data meta-analysis supported web-based interventions’ efficacy for adult problem drinking (Riper et al., 2018). Hai et al.’s systematic review/meta-analysis found supporting evidence for TBIs’ efficacy in preventing and reducing substance use among women of childbearing age (d = 0.19, 95% CI = 0.02, 0.35) (Hai et al., 2019). Lin et al. (2019), Ashford et al. (2020), and Carreiro et al. (2020) also highlighted the acceptability and usability of TBIs, particularly telemedicine and mobile health applications, as effective and accessible tools for adult substance use treatment (Ashford et al., 2020; Carreiro et al., 2020; Lin et al., 2019). Perski et al. (2022) reviewed 14 studies on technology-mediated just-in-time adaptive substance use interventions, reporting moderate-to-high engagement and mixed evidence of effectiveness (Perski et al., 2022). Lastly, Bonfiglio et al.’s (2022) systematic review showed TBIs’ effectiveness in reducing substance use frequency, enhancing abstinence, and reducing dependence severity (Bonfiglio et al., 2022).

However, previous systematic reviews and meta-analyses have often combined emerging adults with younger (adolescents) or older (middle-older adults) age groups, potentially obscuring age-specific intervention effects. Arnett's theory of emerging adulthood posits that the period from 18 to 24 years old represents a distinct developmental stage characterized by identity exploration, instability, self-focus, feeling "in-between," and a sense of possibilities (Arnett, 2000, 2012). These features may make emerging adults respond to interventions differently. For instance, the identity exploration and sense of possibilities characteristic of this stage may make emerging adults more open to behavior change interventions. Conversely, the instability feature of emerging adulthood may present unique challenges for intervention effectiveness. Moreover, the transition to college represents a significant life change for many emerging adults. It is often accompanied by increased independence, exposure to new social environments, and heightened peer influence, which are factors that can significantly impact substance use behaviors (Patrick et al., 2024). Many interventions targeting college-attending emerging adults are specifically designed to address the unique stressors and social norms around substance use prevalent in college settings (Arterberry et al., 2024).

Given these theoretical considerations, it is plausible that the effectiveness of TBIs for substance use may differ for emerging adults from other age groups. By focusing specifically on emerging adults and college students, this meta-analysis aims to provide insights into the effectiveness of TBIs for this theoretically distinct developmental stage. We sought to synthesize the best quality research evidence from RCTs to provide implications for clinical/policy practice and future research by (a) systematically identifying and reviewing RCTs on TBIs for alcohol and drug use and their associated consequences among emerging adults/college students, (b) summarizing key characteristics of the trials and interventions, (c) critically assessing study quality, and (d) utilizing meta-analysis to estimate treatment effect sizes and identifying treatment moderators, including participant characteristics (e.g., age, gender, race/ethnicity, emerging adults/college students), TBI characteristics (e.g., duration, technology type, human contact), control condition, outcome, follow-up timing, and region.

## Materials and Methods

This systematic review was conducted following the Cochrane's guidelines for systematic reviews of interventions and the PRISMA guidelines (PRISMA checklist in Appendix A). The review protocol was prospectively registered with the PROSPERO (ID: CRD42021271078). The template data collection forms, the data extracted from included studies, the data used for all analyses, and the analytic code can be requested from the first author.

### Inclusion Criteria

To be included in the review, studies had to meet several preset criteria across different domains.

#### Type of Interventions

The primary focus was on TBIs designed to address alcohol and/or drug use and associated consequences. TBIs were defined as interventions that engage participants through interactive features, personalized content, or real-time feedback, delivered via platforms such as computers, websites, text messaging, interactive voice recognition, mobile apps, virtual reality, and other emerging technologies. These 'dynamic interventions' range from highly interactive formats, requiring active user engagement, to more passive formats, such as automated text message delivery, which offer scheduled or sequential support without requiring participant responses.

#### Control Conditions

Eligible control conditions included (a) no-treatment or waitlist control groups, (b) treatment-as-usual or standard care, and (c) active interventions that were not technology-based. This range of controls allowed for a broad assessment of TBI efficacy under various conditions.

#### Study Outcomes

The included studies had to report outcomes related to alcohol or drug use. Primary outcomes included alcohol/drug use indicators such as frequency or quantity of use, dosage, or the percentage of days abstinent. Secondary outcomes could involve cravings or substance use–related consequences, measured through instruments such as the Drinker Inventory of Consequences.

#### Study Population and Design

The target population for the studies included emerging adults aged 18–24 and college students. Only studies that employed a randomized controlled trial (RCT) design were included. RCTs were chosen due to their rigorous methodology, which reduces bias and allows for a more reliable assessment of intervention efficacy.

#### Publication Type

Both published and unpublished studies were eligible for inclusion to minimize publication bias and ensure comprehensive coverage of available evidence. This included peer-reviewed journal articles, dissertations, theses, conference proceedings, and other grey literature. Studies needed to be published in English to be included. While this criterion may introduce a risk of language bias, resource limitations prevented the inclusion of non-English studies and translations.

#### Data Reporting

Studies were required to provide sufficient data to calculate effect sizes for meta-analysis. If necessary data were not reported, we contacted the authors for additional information. If no response was received after repeated attempts, the study was excluded from the analysis.

### Exclusion Criteria

#### Ineligible Interventions

Interventions that only provided static information without interactive or personalized elements were excluded. For example, static information, such as non-interactive web pages or brochures, was excluded, as it lacks interactivity, personalization, or systematic engagement essential to dynamic TBIs.

#### Ineligible Control Conditions

Studies comparing a TBI with another TBI were excluded, as the focus of this meta-analysis was to evaluate the efficacy of TBIs relative to no treatment, standard care, or non-technology-based interventions. As a result, technology-based control conditions were considered ineligible.

#### Ineligible Outcomes

Studies that included no outcomes related to alcohol/drug use, craving, or consequence were excluded. Tobacco use, alcohol/drug harm reduction/prevention outcomes, such as substance use attitudes, negative/positive substance use expectancy, and perceived substance use norms, were considered ineligible outcomes.

#### Ineligible Study Population and Design

Studies that did not focus on emerging adults aged 18–24 or college students were excluded. Studies employing non-RCT designs, such as observational studies or quasi-experimental designs, were not eligible for inclusion to maintain methodological consistency across the included studies.

#### Ineligible Publication Type

Non-English publications were excluded, as the scope of this review was limited to English-language studies due to resource constraints.

#### Insufficient Data

Studies that did not report sufficient data for effect size calculation were excluded unless the authors provided the missing information after multiple contact attempts. If no response was received, the study was excluded from this review.

### Search Strategy

Nine electronic databases were searched from inception to July 2024: PubMed, Medline, Embase, Web of Science, CINAHL Complete, PsycINFO, SocINDEX, Cochrane Library (Clinical Trials only), and ProQuest Dissertation and Theses Global. The search was conducted initially on 11/16/2021, updated on 10/31/2022, and again on 7/24/2024. Database-specific search strategies were used for each database (Appendix B). In addition, the reference lists of relevant studies were reviewed to identify additional eligible studies.

### Selection of Studies

Two reviewers independently conducted the title and abstract screening and full-text screening and were blinded to each other's decisions using Covidence.org. The inter-rater agreement rate was 92.9%, calculated as the proportion of studies with consensus among reviewer pairs divided by the total number of studies screened. Disagreements between reviewers were resolved through discussions with a third reviewer.

### Data Extraction

Two independent reviewers extracted data from the included studies using a data extraction form. Extracted data included details regarding the characteristics of the studies, interventions, and participants, assessment outcomes, and associated measurements. We reached out to study authors for unreported data and additional details. Any discrepancies during data extraction were resolved through discussion with a third reviewer.

### Quality Assessment of Included Studies

Two reviewers independently conducted quality assessment utilizing the Cochrane Collaboration’s tool for assessing risk of bias in randomized trials (RoB 2.0). Studies were rated with low risk of bias, some concerns, and high risk of bias in six domains: (1) bias arising from the randomization process, (2) bias due to deviations from intended interventions, (3) bias due to missing outcome data, (4) bias in the measurement of the outcome, (5) bias in the selection of the reported result, and (6) overall risk of bias.

### Data Analysis

Data analysis was conducted following Cooper, Hedge, and Valentine’s meta-analysis handbook (Cooper et al., 2009). We used R Studio to conduct data analysis in four steps: (1) estimating individual between-group effect sizes (with baseline outcomes adjusted when available), (2) synthesizing effect size estimates and conducting moderator analyses using robust variance estimation (RVE) in meta-regression (using the robumeta package), (3) conducting sensitivity analysis, and (4) performing publication bias assessment (using the metafor package).

Effect sizes were estimated at the outcome level using Hedges' g effect size with small sample size correction (noted as *d*) (Cooper et al., 2009). When studies reported multiple relevant outcomes, we calculated separate effect sizes for each outcome to capture the full range of intervention effects. For each study, we extracted the following data for both intervention and control groups: means and standard deviations for continuous variables (using standard errors or confidence intervals to estimate standard deviations when unavailable), and frequencies or percentages for categorical variables. Sample sizes were recorded for all outcomes. In cases where studies provided insufficient data for direct calculation, we used available test statistics (e.g., t test, F statistics, Chi-square statistics, odds ratio) or contacted study authors for additional information. Positive effect sizes indicate improvement in the desired direction (e.g., decrease in drug use frequency). Statistical significance of results was inferred by an alpha level of 0.050 when the degrees of freedom (*df*) $\ge$ four, and an alpha level of 0.010, when the df $<$ four. Cohen’s criteria were used to interpret the magnitude of effect sizes: small effect (d = 0.2), medium effect (d = 0.5), large effect (d = 0.8) (Cohen, 1988).

We used RVE with random-effect model in meta-regression for synthesizing effect sizes and conducting moderator analyses. RVE is a meta-analytic method designed to handle dependent effect sizes, which are common in this review as most studies reported multiple intervention outcomes for each sample (Hedges et al., 2010; Tanner-Smith et al., 2016). RVE makes no assumption about the specific form of effect sizes’ sampling distributions and requires no information about the covariate structure of dependent effect sizes, making it advantageous over other methods such as generalized least squares estimation and multilevel meta-analysis (Hedges et al., 2010; Tanner-Smith & Tipton, 2014). Additionally, simulation studies have shown that RVE can yield accurate effect size estimates with as few as ten studies (Tanner-Smith & Tipton, 2014) and accurate moderator analysis results with 20–40 studies (Hedges et al., 2010; Tipton, 2013).

We estimated an overall effect size by pooling all effect sizes from studies included in this review. We also calculated subgroup effect sizes for 1) control conditions (no treatment/waitlist control, treatment as usual/standard care, non-technology intervention), 2) outcomes (alcohol, drug, unspecified/multiple substance use, consequences), 3) populations (general emerging adults, college students), 4) regions (e.g., North America, Europe, Oceania), 5) technology type (website, email, smartphone application, text messaging, social media, multiple technologies), 6) human contact (none, some), 7) publication type (journal article, dissertation/thesis), and 8) overall risk of bias rating (low risk, some concerns, high risk).

We conducted moderator analyses using RVE in meta-regression to investigate whether intervention effects varied by age, gender (% females in the sample), race/ethnicity, intervention duration (number of weeks, number of sessions), control condition, outcome, population, region, technology, human contact, publication type, and overall risk of bias rating. In addition, we conducted sensitivity analysis by comparing results with and without effect size outliers. We assessed the presence of outliers using standard formulas based on the interquartile range (IQR). Effect sizes that were three IQR below the first quartile or above the third quartile were considered outliers. Forty-three effect sizes met the outlier criteria. Pooled effect sizes with and without outliers were not significantly different from each other (Appendix C). Effect size synthesis and moderator analysis were performed with outliers removed. Follow-up assessment timing (number of weeks after baseline) was adjusted for pooling effect sizes and all meta-regression analyses. Finally, it is recommended to use a combination of methods for a comprehensive evaluation of publication bias, as no single method consistently outperforms the others (Carter et al., 2019). Therefore, we assessed publication bias using multiple methods, including Egger’s Regression Test to detect funnel plot asymmetry, the Selection Model to adjust for study selection bias, and Orwin’s Fail-safe N to estimate the robustness of the meta-analytic findings.

## Results

### Search Results

**Fig. 1:**
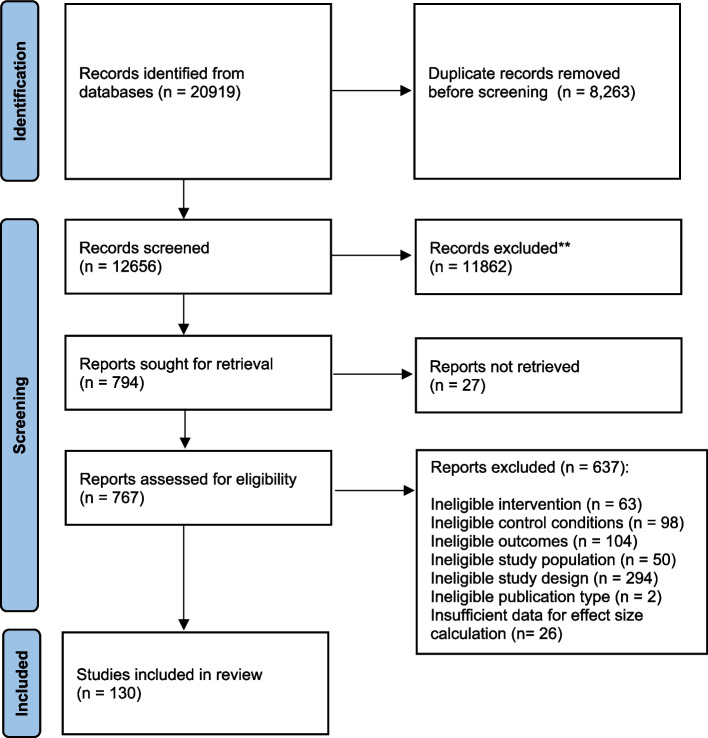
PRISMA flow chart

Figure 1 presents a PRISMA diagram of the screening process. After removing duplicates, 20,919 studies remained for screening; 8,263 studies were excluded based on titles and abstracts; and 637 studies were excluded in the full-text review. Finally, 130 studies met the inclusion criteria and were included in the present review.

### Study Characteristics

Appendix D presents the characteristics of the 130 studies under review, encompassing a total of 63,915 participants, with individual study sample sizes ranging from 18 to 7,809. This review included 119 peer-reviewed journal articles and 11 dissertations, with some articles encompassing multiple studies. More than 58% of the studies were conducted between 2015–2024 (58.5%, *n* = 76), and 41.5% (*n* = 54) between 2004–2013. Studies were from 18 countries, with the majority (*n* = 86, 66%) in the US, followed by the UK (*n* = 9, 6.9%), Sweden (*n* = 7, 5.4%), and Switzerland (*n* = 5, 3.8%). Across the studies that reported sample racial/ethnic compositions, 54.3% of participants were White, 6.4% Latinx, 6.0% Black, 6.9% Asian, and 25.8% other. One hundred and nine studies (*n* = 109, 83.8%) focused on college students, while twenty-one (16.2%) targeted a general emerging adult population. The average percentage of female participants across studies was 55.6%, with four studies focused exclusively on women and four only on men. For controls, 20 studies employed non-technology interventions (e.g., motivational interviewing), 83 studies used no intervention/waitlist control, and 24 studies utilized treatment as usual/standard care, with some studies including multiple types of controls.

### TBI Characteristics

About 70% of the TBIs (*n* = 90, %), 69.2%) utilized web-based/computer platforms. Twelve TBIs utilized text messaging (9.2%), eighteen used email, apps, or social media (13.8%), and nine used multiple forms of technology (6.9%). In terms of intervention content/component, the most frequently employed was personalized normative feedback. Other common intervention components/models included psychoeducation on information such as the consequences of substance use and protective behavioral strategies, motivational interviewing, and cognitive behavioral therapy. Most studies (*n* = 104, 80%) had fully automated TBIs with no human contact, and some (*n* = 25, 19.2%) incorporated human contact (missing information *N* = 1). Eighty-six TBIs’ (66.7%) communication was one-way (TBI providing information to participants), while 43 (33.3%) were two-way communication, enabling participants to respond to texts or feedback (missing information *N* = 1). TBI duration ranged from 0.5 weeks to 64 weeks.

### Quality Assessment Results

**Fig. 2:**
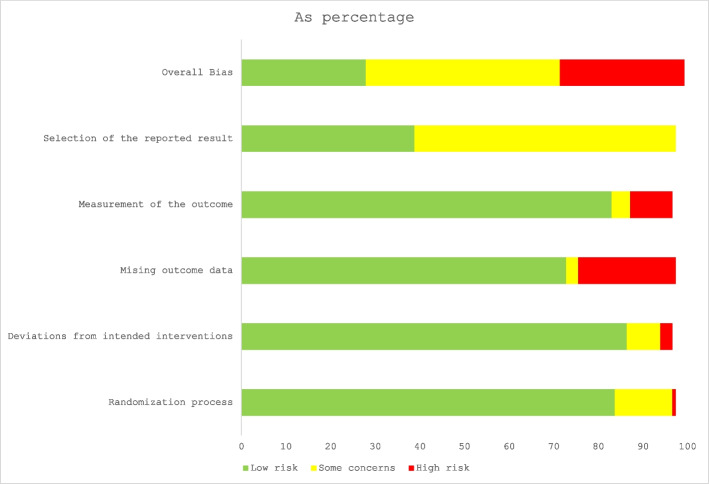
Risk of bias summary

Figure 2 and Appendix E present the risk of bias assessment results. Of the 130 studies under review, 89.2% (*n* = 116) were rated as low risk in the randomization process. Most studies (*n* = 116, 89.2%) were considered low risk for deviations from the intended intervention. Only 2.3% (*n* = 3) were rated high risk due to non-adherence to the intention-to-treat principle or blinding challenges commonly seen in social science. In terms of missing outcome data, 23.8% (*n* = 31) had some concerns or a high risk due to attrition rate above 10% and lack of evidence suggesting that the result was not biased by missing outcome data. For outcome measurement, 9.2% (*n* = 12) were high risk because the outcome assessors were the participants (self-report measures) who were not blinded to their intervention assignments. Regarding the selection of reported results, 53.8% (*n* = 70) raised some concerns, due to insufficient information to determine whether data was analyzed according to a pre-specified analysis plan.

### Meta-Analysis Results

Table 1 presents the overall and subgroup effect size estimates. The overall effect size representing TBIs’ effects on substance use and related consequences compared to controls, with 1194 effect sizes from 130 studies, was *d* = 0.23 (95% CI = 0.18, 0.28). Follow-up assessment timing was not a significant predictor of effect sizes (*b* = −0.001, SE = 0.001, *p* = 0.265). The effect sizes for comparing TBIs with no treatment/waitlist controls and treatment as usual/standard care were *d* = 0.25 (95% CI = 0.19, 0.31) and *d* = 0.23 (95% CI = 0.15–0.32), respectively. The effect size for comparing TBIs with non-technology-based interventions was *d* = 0.12, which was marginally significant but did not reach statistical significance (95% CI = −0.02–0.25). The sizes of TBIs’ effects on alcohol use, drug use, and substance use consequences were *d* = 0.22 (95% CI = 0.17, 0.28), *d* = 0.22 (95% CI = 0.06, 0.38), and *d* = 0.17 (95% CI = 0.05, 0.29), respectively. The effect size for unspecified/multiple substance use outcomes was *d* = 0.26.and not statistically significant (95% CI = −0.18, 0.70). The respective sizes of TBIs’ effects for the general emerging adult population and college student population were *d* = 0.33 (95% CI = 0.18, 0.48) and *d* = 0.21 (95% CI = 0.16, 0.27).

**Table 1:** Overall and subgroup effect size estimates

	Effect size N	Study N	*d*	SE	*t*	*df*	*p*	95% CI
Overall Effect Size	1194	130	0.23*	0.03	9.27	34.68	< .001	0.18–0.28
Control Type								
No treatment/Waitlist control	785	89	0.25*	0.03	8.11	27.71	< .001	0.19–0.31
Treatment as usual/Standard care	270	24	0.23*	0.04	5.84	17.04	< .001	0.15–0.32
Non-technology intervention	137	20	0.12	0.06	1.83	15.54	.087	−0.02–0.25
Outcome								
Alcohol use	859	109	0.22*	0.03	7.79	35.85	< .001	0.17–0.28
Drug use	154	27	0.22*	0.08	3.00	14.85	< .010	0.06–0.38
Unspecified/any substance use	40	10	0.26	0.17	1.50	5.13	.193	−0.18–0.70
Consequences	141	42	0.17*	0.06	2.86	31.00	< .010	0.05–0.29
Population								
Emerging adults	249	21	0.33*	0.07	4.59	16.58	< .001	0.18–0.48
College students	937	109	0.21*	0.03	7.39	73.96	< .001	0.16–0.27
Region								
North America	860	89	0.21*	0.03	6.76	63.56	< .001	0.15–0.27
Europe	207	29	0.19*	0.05	4.18	16.85	< .010	0.10–0.29
Oceania	84	7	**0.34**	0.15	2.34	6.78	.053	−0.01–0.67
South America	31	2	0.31	0.18	1.73	1.00	.333	−1.97–2.59
Technology								
Website/computer-based	766	90	0.20*	0.03	6.64	30.43	< .001	0.14–0.26
Email	88	5	0.31	0.13	2.31	3.39	.094	−0.09–0.71
Smartphone application	78	10	0.08	0.05	1.61	4.80	.170	−0.05–0.21
Text messaging	124	12	0.26*	0.09	3.01	6.68	< .050	0.05–0.46
Social media	77	3	0.37	0.33	1.15	2.35	.353	−0.84–1.59
Multiple technology	61	9	0.47*	0.11	4.40	9.06	< .010	0.23–0.71
Human Contact								
None	1005	104	0.21*	0.03	8.07	30.81	< .001	0.16–0.26
Some	181	25	0.32*	0.06	5.48	19.32	< .001	0.20–0.45
Publication Type								
Journal article	1128	119	0.21*	0.03	7.54	77.72	< .001	0.16–0.27
Dissertation/thesis	52	11	0.17*	0.06	2.83	5.09	< .050	0.02–0.33
Overall risk of bias rating								
Low risk	265	34	0.21*	0.04	5.24	22.54	< .001	0.12–0.30
Some concerns	646	63	0.26*	0.04	6.24	25.78	< .001	0.17–0.34
High risk	275	33	0.19*	0.04	4.56	20.53	< .001	0.11–0.28

*d* small sample corrected Hedge’s g, *SE* standard error, *df* degree of freedom, *CI* confidence interval. * *p* < .050 when df > = 4, * *p* < .010 when df < 4. Control type, outcome, and technology’s study Ns do not add up to 130 because of studies with multiple controls/outcomes/TBIs. Human contact study Ns do not add up to 130 because one study did not report relevant information. Regions (effect size N: Asia = 2; Africa = 10) and technology (effect size N: virtual reality = 3) with small sample sizes were included in the overall effect size calculation but excluded from subgroup effect size calculation. Heterogeneity: Tau^2^ = 0.06 (SE = 0.003), I^2^ = 88.41%, Q(df = 1193) = 9598.19, *p* < .001

The sizes of TBIs’ effects for North America (*d* = 0.21, 95% CI = 0.15, 0.27) and Europe (*d* = 0.19, 95% CI = 0.10, 0.29) were significant. In contrast, the effect size for TBIs in Oceania was marginally significant (*d* = 0.34, 95% CI = −0.01, 0.67), while the effect size for South America was based on only two studies and not significant (*d* = 0.31, 95% CI = −1.97, 2.59). The effect sizes for TBIs using websites/computer (*d* = 0.20, 95% CI = 0.14, 0.26), text messaging (*d* = 0.26, 95% CI = 0.05, 0.46), and multiple technologies (*d* = 0.47, 95% CI = 0.23, 0.71) were significant. In contrast, the effect sizes for TBIs using emails (*d* = 0.31, 95% CI = −0.09, 0.71), smartphone applications (*d* = 0.08, 95% CI = −0.05, 0.21), and social media (*d* = 0.37, 95% CI = −0.84, 1.59) were not significant, potentially due to low power as evidenced by the low degrees of freedom (*df* < 5). The respective effect sizes for TBIs without and with human contact were *d* = 0.21 (95% CI = 0.16, 0.26) and *d* = 0.32 (95% CI = 0.20, 0.45). TBIs’ effect size was *d* = 0.21 (95% CI = 0.16, 0.27) based on evidence from journal articles, and the effect size was *d* = 0.17 (95% CI = 0.02, 0.33) based on data from dissertations and theses. Effect sizes based on evidence from articles with low risk of bias, some concerns, and high risk of bias were *d* = 0.21 (95% CI = 0.12, 0.30), *d* = 0.26 (95% CI = 0.17, 0.34), and *d* = 0.19 (95% CI = 0.11, 0.28), respectively.

Individual effect sizes from primary studies are listed in Appendix F. The heterogeneity statistics revealed substantial variability among the studies: Tau^2^ = 0.06 (SE = 0.003), I^2^ = 88.41%, Q(df = 1193) = 9598.19, *p* < 0.001. To investigate potential sources of this heterogeneity, we conducted a moderator analysis, with the findings detailed in Table 2. Moderator analysis revealed that older participant age was significantly associated with larger TBI effect sizes (*b* = 0.02, *SE* = 0.01, *t* = 2.14, *p* < 0.050). Additionally, compared to TBIs delivered through smartphone applications, those that utilized multiple types of technology had significantly greater effect sizes (*b* = 0.28, *SE* = 0.11, *t* = 2.53, *p* < 0.050). The moderating effect of population (emerging adults vs college students), gender (% female), technology (smartphone application vs text messaging), and human contact (none vs some) were marginally significant (p: 0.053–0.059). TBIs’ effects did not differ significantly as a function of follow-up assessment timing, control type, outcome, race/ethnicity, region, technology, intervention duration, publication type, or overall risk of bias rating.

**Table 2:** Moderator analysis results

	Coefficient	SE	*t*	*df*	*p*
Control Type					
No treatment/Waitlist control vs Treatment as usual/Standard care	−0.02	0.04	−0.59	34.27	.561
No treatment/Waitlist control vs Non-technology intervention	−0.10	0.07	−1.51	25.19	.144
Treatment as usual/Standard care vs Non-technology intervention	−0.08	0.07	−1.16	37.40	.255
Outcome					
Alcohol use vs Drug use	−0.05	0.04	−1.04	27.87	.309
Alcohol use vs Unspecified/Multiple substance use	−0.02	0.10	−0.18	6.25	.866
Alcohol use vs Consequences	−0.06	0.05	−1.20	35.52	.236
Drug use vs Unspecified/Multiple substance use	0.03	0.11	0.27	8.61	.797
Drug use vs Consequences	−0.01	0.06	−0.18	44.06	.857
Unspecified/Multiple substance use vs Consequences	0.04	0.11	0.35	9.31	.731
Population					
Emerging adults vs College students	**−0.11**	0.05	−2.00	25.85	.056
Age	**0.02**	**0.01**	**2.14**	**29.94**	** < .050**
Gender (% female)	**0.002**	0.001	1.96	31.72	.059
Race/Ethnicity					
Asian vs White	0.001	0.003	0.43	14.54	.675
Black vs White	0.000	0.003	0.07	8.73	.949
Latinx vs White	−0.003	0.003	−1.07	8.94	.315
Other vs White	0.00004	0.001	−0.06	45.36	.954
Region					
North America vs Europe	−0.04	0.05	−0.81	50.38	.419
North America vs Oceania	0.13	0.13	1.05	8.33	.323
North America vs South America	0.06	0.15	0.43	1.05	.736
Europe vs Oceania	0.17	0.13	1.33	10.19	.212
Europe vs South America	0.10	0.15	0.69	1.13	.605
Oceania vs South America	−0.07	0.19	−0.37	1.62	.754
Technology					
Website vs Email	0.05	0.10	0.48	5.95	.650
Website vs Smartphone application	−0.09	0.05	−1.88	9.43	.091
Website vs Text messaging	0.07	0.07	1.02	13.83	.326
Website vs Social media	0.11	0.16	0.68	2.31	.561
Website vs Multiple technology	0.19	0.11	1.82	11.01	.096
Email vs Smartphone application	−0.13	0.10	−1.28	10.34	.227
Email vs Text messaging	0.03	0.12	0.22	10.48	.828
Email vs Social media	0.06	0.18	0.32	4.90	.764
Email vs Multiple technology	0.14	0.14	1.02	11.44	.330
Smartphone application vs Text messaging	**0.16**	0.08	2.03	17.77	.057
Smartphone application vs Social media	0.19	0.16	1.20	3.65	.302
Smartphone application vs Multiple technology	**0.28**	**0.11**	**2.53**	**16.89**	** < .050**
Text messaging vs Social media	0.03	0.17	0.19	3.55	.862
Text messaging vs Multiple technology	0.12	0.12	0.95	19.22	.355
Social media vs Multiple technology	0.09	0.19	0.46	3.95	.672
Human Contact					
None vs Some	**0.11**	0.06	2.00	33.68	.053
Intervention duration (# of weeks)	−0.004	0.003	−1.39	3.16	.253
Intervention duration (# of sessions)	0.0003	0.001	0.39	2.70	.727
Publication Type					
Journal Article vs Dissertation/Thesis	0.03	0.06	0.45	11.76	.658
Overall risk of bias rating					
Low risk vs Some concerns	0.04	0.05	0.91	57.93	.365
Low risk vs High risk	−0.02	0.05	−0.38	59.08	.706
Some concerns vs High risk	−0.06	0.05	−1.24	60.42	.220

Follow-up assessment timing, age, and % of female participants, and intervention duration were mean-centered. *SE* standard error, *df* degrees of freedom, *CI* confidence interval. * *p* < .05 when df > = 4, * *p* < .01 when df < 4

In assessing publication bias, Egger's Regression Test revealed a statistically significant intercept of 0.09 (SE = 0.02, t = 5.80, *p* < 0.001), indicating asymmetry in the funnel plot and suggesting potential publication bias, with smaller studies potentially reporting larger effect sizes. The selection model analysis further supported the likelihood of publication bias. The test for selection model parameters yielded a significant likelihood ratio test (LRT = 11.98, df = 1, *p* < 0.001), suggesting an underrepresentation of studies with less significant findings, which may have inflated the observed effect size. Finally, Orwin's Fail-safe N calculation showed that 1,297 additional "null" studies (effect size = 0) would be required to reduce the observed average effect size from 0.21 (i.e., pooled overall effect size without adjusting for assessment timing) to the target effect size of 0.10, indicating that the results are relatively robust despite the observed signs of publication bias.

## Discussion

This review integrates literatures that are often compartmentalized into college and non-college samples or adolescent and adult samples, focusing on emerging adulthood, and takes a global perspective. Synthesizing evidence from 131 RCTs, this review supports the effectiveness of TBIs, compared to control conditions, in reducing substance use and related consequences among emerging adults and college students, with an overall effect size of *d* = 0.23. While an effect size of 0.2 is considered small according to Cohen's benchmarks (Cohen, 1988), it aligns with the typical range observed in psychosocial interventions for substance use (Carney & Myers, 2012; Dutra et al., 2008; Tanner-Smith & Lipsey, 2015; Tanner-Smith et al., 2022). More importantly, this level of effect is often meaningful in the context of addiction treatment and prevention, where even modest improvements can have significant practical implications (E. Carey et al., 2023; Götz et al., 2022).

Effect sizes from the present review align closely with those reported in prior meta-analyses on TBIs. The overall effect size in this review, along with effect sizes across different control conditions, populations, regions, technology types, and publication formats, are consistent with previous findings for TBIs targeting both young people and adults. These prior meta-analyses typically report small-to-moderate effect sizes, with TBI effect sizes ranging from d = 0.19 to d = 0.30 for adult populations (Hai et al., 2019; Kiluk et al., 2019), d = 0.12 to d = 0.57 for adolescents and emerging adults (Champion et al., 2019; O’Logbon et al., 2023; Tait & Christensen, 2010), and d = 0.10 to d = 0.38 for college students (K. B. Carey et al., 2009, 2012; Gulliver et al., 2015). These results indicate that TBI effects are consistent across age groups. Although TBI effects on substance use outcomes are generally modest, they have meaningful implications for public health due to the scalability and accessibility of TBIs.

The majority of the RCTs under review compared TBIs with no treatment/waitlist controls and fewer used treatment as usual/standard care and non-technology interventions. TBIs demonstrated advantages over no treatment/waitlist controls (*d* = 0.25) and treatment as usual/standard care (*d* = 0.23). When compared to non-technology interventions, the effect size (*d* = 0.12) was marginal but not significant, suggesting that TBIs might perform at least as well as traditional non-technology interventions. Carey et al. (2012) similarly found that both face-to-face and computer-delivered interventions were equally effective in reducing overall alcohol consumption and related issues among college students. While Carey’s review notes subtle variations in specific outcomes—such as a slightly greater impact of face-to-face interventions on peak blood alcohol levels at intermediate assessments and a modest advantage of computer-delivered interventions in reducing heavy drinking frequency at long-term follow-up—both reviews ultimately reinforce the conclusion that TBIs are a viable alternative to traditional interventions. In terms of specific outcomes, TBIs’ effects on alcohol use (*d* = 0.22), drug use (*d* = 0.22), and substance use consequences (*d* = 0.17) outperformed control conditions. It is also noteworthy that a significantly larger number of studies investigated the effects of TBIs on alcohol use compared to drug use. There is a need for further research on TBIs for drug-related issues.

Most of the included studies focused on web/computer-based interventions, and some explored TBIs delivered through text messages or a combination of multiple technologies. This review indicates that web/computer-based interventions, text messaging, and interventions utilizing multiple technologies are effective in mitigating substance use, with effect sizes ranging from small to moderate (d = 0.20 for web/computer, d = 0.26 for text messaging, and d = 0.47 for multiple technologies). Notably, interventions employing multiple technologies demonstrated significantly greater effect sizes compared to those delivered through smartphone applications alone. This could be attributed to the potentially more engaging nature of multi-platform approaches or the increased intensity of interventions utilizing various technologies. Future research should systematically compare single-technology interventions with multi-platform approaches to determine whether this advantage in effectiveness holds in different contexts while also considering cost, accessibility, and feasibility. In addition, the current evidence base is insufficient to draw definitive conclusions about the effectiveness of TBIs using email, smartphone applications, social media, or emerging technologies such as virtual reality and artificial intelligence. Given the observed efficacy of TBIs employing established technologies, further research exploring the potential of newer technological platforms in substance use interventions is warranted.

We also found that within the emerging adult and college student demographic, older participants exhibited larger effect sizes in response to TBIs for substance use. This aligns with findings in broader adult populations, where individuals aged 55 and above have demonstrated better treatment responses to TBIs compared to younger people (Riper et al., 2018). Several factors may contribute to this pattern: older emerging adults may possess enhanced cognitive abilities and decision-making skills, enabling them to engage more effectively with intervention content; increased life experience can lead to a greater recognition of the negative consequences associated with substance use, fostering a stronger commitment to change; and older individuals might have heightened motivation to adopt healthier behaviors, possibly due to impending transitions into professional careers or family life (Arnett, 2000). Future research should explore the underlying mechanisms driving these age-related differences to inform the development of age-appropriate interventions.

The majority of studies included in this review were conducted in North American and European countries, primarily due to our focus on studies published in English. Our results lend support to the effectiveness of TBIs in addressing emerging adult/college student substance use in North America (*d* = 0.21), Europe (*d* = 0.19), and Oceania (*d* = 0.34). We recommend that future reviews broaden their scope to encompass TBI studies in non-English languages to examine TBIs for emerging adults/ college students across different socio-cultural contexts. Comparative analyses across various regions and languages can shed light on the potential adaptations and cultural considerations necessary for optimizing the effectiveness of TBIs on a global scale.

The findings of this review must be interpreted in light of its limitations. First, despite the rigorous and systematic search and screening procedures employed, some studies meeting the inclusion criteria may not have been identified. Secondly, while the studies included in this review utilized a variety of measures for each outcome construct, our approach aggregated effect sizes at the more generalized construct level (i.e., alcohol use, drug use, unspecified substance use, consequences) rather than on specific measures. This decision was necessitated by the relatively limited number of effect size estimates available for each specific measure, which, if analyzed separately, would have resulted in a diminished statistical power. Thirdly, our evidence is limited to English-language studies, primarily from Western countries. Caution should be exercised when generalizing these findings to other countries and cultures. Fourthly, we did not include specific intervention components or participants' baseline consumption status (e.g., substance use disorder diagnosis) as moderators, due to the limited availability of relevant data from primary studies. Future research should investigate whether, and how, TBI effectiveness varies by these factors to inform the development of more tailored and effective interventions.

## Conclusions

This review supports the effectiveness of TBIs in reducing substance use and its related consequences among emerging adults and college students. TBIs demonstrated advantages over no treatment/waitlist controls, as well as treatment-as-usual or standard care conditions, and showed comparable effectiveness to traditional non-technology-based interventions. While most studies focused on alcohol-related outcomes, the paucity of research on TBIs for drug-related issues highlights an important gap for future exploration. Interventions employing multiple technologies showed greater effectiveness compared to smartphone app-based interventions, suggesting the need to compare TBIs across different platforms. Additionally, evidence remains limited for TBIs using email, smartphone applications, social media, or emerging technologies such as virtual reality and artificial intelligence, necessitating further research in these areas. Age was identified as a moderator of TBI effectiveness, with older participants within the emerging adult demographic exhibiting larger effect sizes, underscoring the importance of developing age-appropriate interventions. Finally, while most studies originated from North America and Europe, future research should address cultural and regional differences to ensure broader applicability and effectiveness of TBIs globally.

## Supplementary Information

Below is the link to the electronic supplementary material.

Supplementary file1 (DOCX 27 KB)

Supplementary file2 (DOCX 29 KB)

Supplementary file3 (DOCX 15.4 KB)

Supplementary file4 (DOCX 193 KB)

Supplementary file5 (PDF 118 KB)

Supplementary file6 (PDF 95 KB)

## Data Availability

The raw coding data may be obtained upon request from the corresponding author.
