# Supplementary material for: Technology-Based Substance Use Interventions for Emerging Adults and College Students: A Systematic Review and Meta-Analysis
Source: Int J Ment Health Addict. 2024 Dec 26;24(1):603–22. doi: 10.1007/s11469-024-01433-7 (PMC12456661; doi:10.1007/s11469-024-01433-7)
Supplement: Supplementary file 2 — Supplementary file2 (DOCX 29 KB) [file 11469_2024_1433_MOESM2_ESM.docx]

**Appendix B- Search Strategies**

**PubMed**

| **Subject** | **Search #** | **Search Strategy** |
| --- | --- | --- |
| Substance use | 1 | "Substance-Related Disorders"[Mesh] OR "Alcohol-Related Disorders"[Mesh] OR "Alcoholic Intoxication"[Mesh] OR Alcoholism[Mesh] OR "Binge Drinking"[Mesh] OR "Amphetamine-Related Disorders"[Mesh] OR "Cocaine-Related Disorders"[Mesh] OR "Drug Overdose"[Mesh] OR "Inhalant Abuse"[Mesh] OR "Marijuana Abuse"[Mesh] OR "Narcotic-Related Disorders"[Mesh] OR "Opioid-Related Disorders"[Mesh] OR "Phencyclidine Abuse"[Mesh] OR "Substance Abuse, Intravenous"[Mesh] OR "Substance Abuse, Oral"[Mesh] OR ((substance[tiab] OR alcohol*[tiab] OR tobacco[tiab] OR drug[tiab] OR smok*[tiab]) AND (use[tiab] OR usage[tiab] OR user[tiab] OR misuse*[tiab] OR initiat*[tiab] OR abus*[tiab] OR problem*[tiab] OR heavy[tiab] OR binge[tiab] OR disorder*[tiab] OR dependen*[tiab] OR frequen*[tiab])) |
| technology-based interventions | 2 | (digital*[tiab] OR technol*[tiab] OR sensor*[tiab] OR robot*[tiab] OR internet*[tiab] OR "social media"[tiab] OR smartphone*[tiab] OR "smart phone*"[tiab] OR telephone*[tiab] OR phone*[tiab] OR online[tiab] OR ipad*[tiab] OR computer*[tiab] OR electronic*[tiab] OR Web[tiab]) OR ROBOTICS[Mesh] OR INTERNET[Mesh] OR "Social Media"[Mesh] OR TELEPHONE[Mesh] OR Smartphone[Mesh] OR "Computers, Handheld"[Mesh] OR COMPUTERS[Mesh] |
| Young adults/ college students | 3 | "young adult"[Mesh] OR (((college[tiab] OR undergraduate*[tiab] OR postsecondary[tiab] OR "post secondary"[tiab] OR post-secondary[tiab] OR university[tiab]) AND (age[tiab] OR aged[tiab] OR student*[tiab])) OR ((young[tiab] OR emerging[tiab]) AND (adult*[tiab] OR adulthood[tiab] OR people[tiab] OR person[tiab])) OR undergraduate*[tiab]) |
| randomized controlled trials | 4 | ("randomized controlled trial"[Publication Type] OR "controlled clinical trial"[Publication Type] OR "randomized"[Title/Abstract] OR "placebo"[Title/Abstract] OR "drug therapy"[MeSH Subheading] OR "randomly"[Title/Abstract] OR ("trial"[Title/Abstract] OR "groups"[Title/Abstract])) NOT ("animals"[MeSH Terms] NOT "humans"[MeSH Terms]) |
| Substance use AND technology-based interventions AND young adults/college students | 5 | #1 AND #2 AND #3 |
| #5 AND randomized controlled trials | 6 | #5 AND #4 |

**Medline**

| **Subject** | **Search #** | **Search Strategy** |
| --- | --- | --- |
| Substance use | 1 | (Substance-Related Disorders/ OR exp Alcohol-Related Disorders/ OR Alcoholic Intoxication/ OR Alcoholism/ OR Binge Drinking/ OR Amphetamine-Related Disorders/ OR Cocaine-Related Disorders/ OR Drug Overdose/ OR Inhalant Abuse/ OR Marijuana Abuse/ OR Narcotic-Related Disorders/ OR exp Opioid-Related Disorders/ OR Phencyclidine Abuse/ OR Substance Abuse, Intravenous/ OR Substance Abuse, Oral/) OR ((substance or alcohol* or tobacco or drug or smok*) adj3 ("use" or usage or user or misuse* or initiat* or abus* or problem* or heavy or binge or disorder* or dependen* or frequen*).ti,ab.) |
| Technology | 2 | (digital* or technol* or sensor* or robot* or internet* or social media or smartphone* or smart phone* or telephone* or phone* or online or ipad* or computer* or electronic* or Web).ti,ab. OR (ROBOTICS/ or INTERNET/ or exp Social Media/ or TELEPHONE/ or Smartphone/ or Computers, Handheld/ or COMPUTERS/) |
| young adults/ college students | 3 | exp young adult/ OR ((((college or undergraduate* or postsecondary or "post secondary" or post-secondary or university) adj3 (age or aged or student*)) or ((young or emerging) and (adult* or adulthood or people or person)) or undergraduate*).ab,ti.) |
| substance use and technology and young adults | 4 | 1 and 2 and 3 |
| RCTs | 5 | (((randomized controlled trial or controlled clinical trial).pt. or (Randomized or placebo or randomly or trial or groups).ab. or (drug therapy).fs.) not (exp animals/ not exp humans/)) |
| 17 and RCTs | 6 | 4 and 5 |

**Embase**

| **Subject** | **Search #** | **Search Strategy** |
| --- | --- | --- |
| Substance use | 1 | drug dependence'/exp OR 'drug dependence' OR 'alcoholism'/exp OR 'alcoholism' OR 'alcohol consumption'/exp OR 'alcohol consumption' OR 'alcohol intoxication'/exp OR 'alcohol intoxication' OR 'binge drinking'/exp OR 'binge drinking' OR 'alcohol abuse'/exp OR 'alcohol abuse' OR 'drug abuse'/exp OR 'drug abuse' OR 'amphetamine dependence'/exp OR 'amphetamine dependence' OR 'cocaine dependence'/exp OR 'cocaine dependence' OR 'drug overdose'/exp OR 'drug overdose' OR 'inhalant abuse'/exp OR 'inhalant abuse' OR 'cannabis addiction'/exp OR 'cannabis addiction' OR 'drug induced disease'/exp OR 'drug induced disease' OR 'opiate addiction'/exp OR 'opiate addiction' OR 'phencyclidine abuse'/exp OR 'phencyclidine abuse' OR 'substance use'/exp OR 'substance use' OR 'substance abuse'/exp OR 'substance abuse' OR ((substance OR alcohol* OR tobacco OR drug OR smok*) NEAR/3 (use OR usage OR user OR misuse* OR initiat* OR abus* OR problem* OR heavy OR binge OR disorder* OR dependen* OR frequen*)) |
| Technology | 2 | robotics'/exp/mj OR 'robotics'/exp OR 'robotics' OR 'internet'/exp/mj OR 'internet'/exp OR 'internet' OR 'social media'/exp OR 'social media' OR 'telephone'/mj/exp OR 'telephone'/exp OR 'telephone' OR 'smartphone'/exp OR 'smartphone' OR 'personal digital assistant'/exp OR 'personal digital assistant' OR 'computer'/exp/mj OR 'computer'/exp OR 'computer' OR 'information technology device'/exp OR 'information technology device' OR 'technology'/exp OR 'technology' OR 'web-based intervention'/exp OR 'web-based intervention' OR digital*:ti,ab OR technol*:ti,ab OR sensor*:ti,ab OR robot*:ti,ab OR internet*:ti,ab OR 'social media':ti,ab OR smartphone*:ti,ab OR 'smart phone*':ti,ab OR telephone*:ti,ab OR phone*:ti,ab OR online:ti,ab OR ipad*:ti,ab OR computer*:ti,ab OR electronic*:ti,ab OR web:ti,ab |
| Young adult | 3 | ((college OR undergraduate* OR postsecondary OR 'post secondary' OR 'post secondary' OR university) NEAR/3 (age OR aged OR student*)) OR ((young:ti,ab OR emerging:ti,ab) AND (adult*:ti,ab OR adulthood:ti,ab OR people:ti,ab OR person:ti,ab)) OR undergraduate*:ti,ab OR 'young adult'/exp OR 'young adult' |
| RCTs (Embase.com filter from Cochrane) | 4 | ‘randomized controlled trial’/de OR ‘controlled clinical trial’/de OR random*:ti,ab,tt OR ‘randomization’/de OR ‘intermethod comparison’/de OR placebo:ti,ab,tt OR (compare:ti,tt OR compared:ti,tt OR comparison:ti,tt) OR ((evaluated:ab OR evaluate:ab OR evaluating:ab OR assessed:ab OR assess:ab) AND (compare:ab OR compared:ab OR comparing:ab OR comparison:ab)) OR (open NEXT/1 label):ti,ab,tt OR ((double OR single OR doubly OR singly) NEXT/1 (blind OR blinded OR blindly)):ti,ab,tt OR ‘double blind procedure’/de OR (parallel NEXT/1 group*):ti,ab,tt OR (crossover:ti,ab,tt OR ‘cross over’:ti,ab,tt) OR ((assign* OR match OR matched OR allocation) NEAR/6 (alternate OR group OR groups OR intervention OR interventions OR patient OR patients OR subject OR subjects OR participant OR participants)):ti,ab,tt OR (assigned:ti,ab,tt OR allocated:ti,ab,tt) OR (controlled NEAR/8 (study OR design OR trial)):ti,ab,tt OR (volunteer:ti,ab,tt OR volunteers:ti,ab,tt) OR ‘human experiment’/de OR Trial:ti,tt |
|  | 5 | (((random* NEXT/1 sampl* NEAR/8 (‘cross section*’ OR questionnaire* OR survey OR surveys OR database or databases)):ti,ab,tt) NOT (‘comparative study’/de OR ‘controlled study’/de OR ‘randomised controlled’:ti,ab,tt OR ‘randomized controlled’:ti,ab,tt OR ‘randomly assigned’:ti,ab,tt)) OR (‘cross‐sectional study’/de NOT (‘randomized controlled trial’/de OR ‘controlled clinical study’/de OR ‘controlled study’/de OR ‘randomised controlled’:ti,ab,tt OR ‘randomized controlled’:ti,ab,tt OR ‘control group’:ti,ab,tt OR ‘control groups’:ti,ab,tt)) OR (‘case control*’:ti,ab,tt AND random*:ti,ab,tt NOT (‘randomised controlled’:ti,ab,tt OR ‘randomized controlled’:ti,ab,tt)) OR (‘systematic review’:ti,tt NOT (trial:ti,tt OR study:ti,tt)) OR (nonrandom*:ti,ab,tt NOT random*:ti,ab,tt) OR ‘random field*’:ti,ab,tt OR (‘random cluster’ NEAR/4 sampl*):ti,ab,tt OR (review:ab AND review:it NOT trial:ti,tt) OR (‘we searched’:ab AND (review:ti,tt OR review:it)) OR ‘update review’:ab OR (databases NEAR/5 searched):ab OR ((rat:ti,tt OR rats:ti,tt OR mouse:ti,tt OR mice:ti,tt OR swine:ti,tt OR porcine:ti,tt OR murine:ti,tt OR sheep:ti,tt OR lambs:ti,tt OR pigs:ti,tt OR piglets:ti,tt OR rabbit:ti,tt OR rabbits:ti,tt OR cat:ti,tt OR cats:ti,tt OR dog:ti,tt OR dogs:ti,tt OR cattle:ti,tt OR bovine:ti,tt OR monkey:ti,tt OR monkeys:ti,tt OR trout:ti,tt OR marmoset*:ti,tt) AND ‘animal experiment’/de) OR (‘animal experiment’/de NOT (‘human experiment’/de OR ‘human’/de)) |
|  | 6 | #4 NOT #5 |
| Substance use AND Technology AND young adult | 7 | #1 AND #2 AND #3 |
| w/ RCTs | 8 | #6 AND #7 |

**Web of Science**

| **Subject** | **Search #** | **Search Strategy** |
| --- | --- | --- |
| Substance Use | 1 | TS="Substance use" OR ALL="Substance-Related Disorders" OR ALL="Alcohol-Related Disorders" OR ALL="Alcoholic Intoxication" OR ALL=Alcoholism OR ALL="Binge Drinking" OR ALL="Amphetamine-Related Disorders" OR ALL="Cocaine-Related Disorders" OR ALL="Drug Overdose" OR ALL="Inhalant Abuse" OR ALL="Marijuana Abuse" OR ALL="Narcotic-Related Disorders" OR ALL="Opioid-Related Disorders" OR ALL="Phencyclidine Abuse" OR ALL="Substance Abuse, Intravenous" OR ALL="Substance Abuse, Oral" OR (((TI=substance OR AB=substance) OR (TI=alcohol* OR AB=alcohol*) OR (TI=tobacco OR AB=tobacco) OR (TI=drug OR AB=drug) OR (TI=smok* OR AB=smok*)) AND ((TI=use OR AB=use) OR (TI=usage OR AB=usage) OR (TI=user OR AB=user) OR (TI=misuse* OR AB=misuse*) OR (TI=initiat* OR AB=initiat*) OR (TI=abus* OR AB=abus*) OR (TI=problem* OR AB=problem*) OR (TI=heavy OR AB=heavy) OR (TI=binge OR AB=binge) OR (TI=disorder* OR AB=disorder*) OR (TI=dependen* OR AB=dependen*) OR (TI=frequen* OR AB=frequen*))) |
| Technology | 2 | ((TI=digital* OR AB=digital*) OR (TI=technol* OR AB=technol*) OR (TI=sensor* OR AB=sensor*) OR (TI=robot* OR AB=robot*) OR (TI=internet* OR AB=internet*) OR (TI="social media" OR AB="social media") OR (TI=smartphone* OR AB=smartphone*) OR (TI="smart phone*" OR AB="smart phone*") OR (TI=telephone* OR AB=telephone*) OR (TI=phone* OR AB=phone*) OR (TI=online OR AB=online) OR (TI=ipad* OR AB=ipad*) OR (TI=computer* OR AB=computer*) OR (TI=electronic* OR AB=electronic*) OR (TI=Web OR AB=Web)) OR ALL=ROBOTICS OR ALL=INTERNET OR ALL="Social Media" OR ALL=TELEPHONE OR ALL=Smartphone OR ALL="Computers, Handheld" OR ALL=COMPUTERS |
| Young Adults | 3 | ALL="young adult" OR ((((TI=college OR AB=college) OR (TI=undergraduate* OR AB=undergraduate*) OR (TI=postsecondary OR AB=postsecondary) OR (TI="post secondary" OR AB="post secondary") OR (TI=post-secondary OR AB=post-secondary) OR (TI=university OR AB=university)) AND ((TI=age OR AB=age) OR (TI=aged OR AB=aged) OR (TI=student* OR AB=student*))) OR (((TI=young OR AB=young) OR (TI=emerging OR AB=emerging)) AND ((TI=adult* OR AB=adult*) OR (TI=adulthood OR AB=adulthood) OR (TI=people OR AB=people) OR (TI=person OR AB=person))) OR (TI=undergraduate* OR AB=undergraduate*)) |
|  | 4 | #1 AND #2 AND #3 |
| RCT filter | 5 | TS=(randomised OR randomized OR randomisation OR randomisation OR placebo* OR (random* AND (allocat* OR assign*) ) OR (blind* AND (single OR double OR treble OR triple) )) NOT TS=(animal or animals or pisces or fish or fishes or catfish or catfishes or sheatfish or silurus or arius or heteropneustes or clarias or gariepinus or fathead minnow or fathead minnows or pimephales or promelas or cichlidae or trout or trouts or char or chars or salvelinus or salmo or oncorhynchus or guppy or guppies or millionfish or poecilia or goldfish or goldfishes or carassius or auratus or mullet or mullets or mugil or curema or shark or sharks or cod or cods or gadus or morhua or carp or carps or cyprinus or carpio or killifish or eel or eels or anguilla or zander or sander or lucioperca or stizostedion or turbot or turbots or psetta or flatfish or flatfishes or plaice or pleuronectes or platessa or tilapia or tilapias or oreochromis or sarotherodon or common sole or dover sole or solea or zebrafish or zebrafishes or danio or rerio or seabass or dicentrarchus or labrax or morone or lamprey or lampreys or petromyzon or pumpkinseed or pumpkinseeds or lepomis or gibbosus or herring or clupea or harengus or amphibia or amphibian or amphibians or anura or salientia or frog or frogs or rana or toad or toads or bufo or xenopus or laevis or bombina or epidalea or calamita or salamander or salamanders or newt or newts or triturus or reptilia or reptile or reptiles or bearded dragon or pogona or vitticeps or iguana or iguanas or lizard or lizards or anguis fragilis or turtle or turtles or snakes or snake or aves or bird or birds or quail or quails or coturnix or bobwhite or colinus or virginianus or poultry or poultries or fowl or fowls or chicken or chickens or gallus or zebra finch or taeniopygia or guttata or canary or canaries or serinus or canaria or parakeet or parakeets or grasskeet or parrot or parrots or psittacine or psittacines or shelduck or tadorna or goose or geese or branta or leucopsis or woodlark or lullula or flycatcher or ficedula or hypoleuca or dove or doves or geopelia or cuneata or duck or ducks or greylag or graylag or anser or harrier or circus pygargus or red knot or great knot or calidris or canutus or godwit or limosa or lapponica or meleagris or gallopavo or jackdaw or corvus or monedula or ruff or philomachus or pugnax or lapwing or peewit or plover or vanellus or swan or cygnus or columbianus or bewickii or gull or chroicocephalus or ridibundus or albifrons or great tit or parus or aythya or fuligula or streptopelia or risoria or spoonbill or platalea or leucorodia or blackbird or turdus or merula or blue tit or cyanistes or pigeon or pigeons or columba or pintail or anas or starling or sturnus or owl or athene noctua or pochard or ferina or cockatiel or nymphicus or hollandicus or skylark or alauda or tern or sterna or teal or crecca or oystercatcher or haematopus or ostralegus or shrew or shrews or sorex or araneus or crocidura or russula or european mole or talpa or chiroptera or bat or bats or eptesicus or serotinus or myotis or dasycneme or daubentonii or pipistrelle or pipistrellus or cat or cats or felis or catus or feline or dog or dogs or canis or canine or canines or otter or otters or lutra or badger or badgers or meles or fitchew or fitch or foumart or foulmart or ferrets or ferret or polecat or polecats or mustela or putorius or weasel or weasels or fox or foxes or vulpes or common seal or phoca or vitulina or grey seal or halichoerus or horse or horses or equus or equine or equidae or donkey or donkeys or mule or mules or pig or pigs or swine or swines or hog or hogs or boar or boars or porcine or piglet or piglets or sus or scrofa or llama or llamas or lama or glama or deer or deers or cervus or elaphus or cow or cows or bos taurus or bos indicus or bovine or bull or bulls or cattle or bison or bisons or sheep or sheeps or ovis aries or ovine or lamb or lambs or mouflon or mouflons or goat or goats or capra or caprine or chamois or rupicapra or leporidae or lagomorpha or lagomorph or rabbit or rabbits or oryctolagus or cuniculus or laprine or hares or lepus or rodentia or rodent or rodents or murinae or mouse or mice or mus or musculus or murine or woodmouse or apodemus or rat or rats or rattus or norvegicus or guinea pig or guinea pigs or cavia or porcellus or hamster or hamsters or mesocricetus or cricetulus or cricetus or gerbil or gerbils or jird or jirds or meriones or unguiculatus or jerboa or jerboas or jaculus or chinchilla or chinchillas or beaver or beavers or castor fiber or castor canadensis or sciuridae or squirrel or squirrels or sciurus or chipmunk or chipmunks or marmot or marmots or marmota or suslik or susliks or spermophilus or cynomys or cottonrat or cottonrats or sigmodon or vole or voles or microtus or myodes or glareolus or primate or primates or prosimian or prosimians or lemur or lemurs or lemuridae or loris or bush baby or bush babies or bushbaby or bushbabies or galago or galagos or anthropoidea or anthropoids or simian or simians or monkey or monkeys or marmoset or marmosets or callithrix or cebuella or tamarin or tamarins or saguinus or leontopithecus or squirrel monkey or squirrel monkeys or saimiri or night monkey or night monkeys or owl monkey or owl monkeys or douroucoulis or aotus or spider monkey or spider monkeys or ateles or baboon or baboons or papio or rhesus monkey or macaque or macaca or mulatta or cynomolgus or fascicularis or green monkey or green monkeys or chlorocebus or vervet or vervets or pygerythrus or hominoidea or ape or apes or hylobatidae or gibbon or gibbons or siamang or siamangs or nomascus or symphalangus or hominidae or orangutan or orangutans or pongo or chimpanzee or chimpanzees or pan troglodytes or bonobo or bonobos or pan paniscus or gorilla or gorillas or troglodytes) |
| w/ RCTs | 6 | #4 AND #5 |

**CINAHL**

| **Subject** | **Search #** | **Search Strategy** |
| --- | --- | --- |
| Substance use | 1 | (MH "Substance Use (Omaha)") OR (MH "Overdose+") OR (MH "Opiate Overdose") OR (MH "Drug Abuse (Saba CCC)") OR (MH "Addictions Nursing") OR (MH "Substance Abuse (Saba CCC)+") OR (MH "Substance Use Disorders+") OR (MH "Substance Abuse, Intravenous") OR (MH "Substance Abusers+") OR (MH "Substance Dependence+") OR (MH "Substance Abuse+") OR (MH "Inhalant Abuse") OR (MH "Alcohol-Related Disorders+") OR (MH "Alcoholic Intoxication+") OR (MH "Alcoholism") OR (MH "Alcoholic Beverages+") OR (MH "Alcoholics") OR (MH "Alcohol Drinking+") OR (MH "Alcohol Abuse+") OR (MH "Binge Drinking") OR (MH "Drinking Behavior+") OR (MH "Recreational Drug Use") OR (MH "Intravenous Drug Users") OR (MH "Street Drugs+") OR (((TI substance OR AB substance) OR (TI alcohol* OR AB alcohol*) OR (TI tobacco OR AB tobacco) OR (TI drug OR AB drug) OR (TI smok* OR AB smok*)) AND ((TI use OR AB use) OR (TI usage OR AB usage) OR (TI user OR AB user) OR (TI misuse* OR AB misuse*) OR (TI initiat* OR AB initiat*) OR (TI abus* OR AB abus*) OR (TI problem* OR AB problem*) OR (TI heavy OR AB heavy) OR (TI binge OR AB binge) OR (TI disorder* OR AB disorder*) OR (TI dependen* OR AB dependen*) OR (TI frequen* OR AB frequen*))) |
| Technology | 2 | ((TI digital* OR AB digital*) OR (TI technol* OR AB technol*) OR (TI sensor* OR AB sensor*) OR (TI robot* OR AB robot*) OR (TI internet* OR AB internet*) OR (TI "social media" OR AB "social media") OR (TI smartphone* OR AB smartphone*) OR (TI "smart phone*" OR AB "smart phone*") OR (TI telephone* OR AB telephone*) OR (TI phone* OR AB phone*) OR (TI online OR AB online) OR (TI ipad* OR AB ipad*) OR (TI computer* OR AB computer*) OR (TI electronic* OR AB electronic*) OR (TI Web OR AB Web)) OR (MH "Robotics+") OR (MH "Technology+") OR (MH "Digital Technology+") OR (MH "Information Technology+") OR (MH "Internet+") OR (MH "Internet-Based Intervention") OR (MH "Social Media+") OR (MH "Telephone+") OR (MH "Cellular Phone+") OR (MH "Smartphone") OR (MH "Mobile Applications") OR (MH "Computers, Hand-Held+") OR (MH "Text Messaging+") OR (MH "Computers and Computerization+") |
| Young adults or college students | 3 | (MH "Young Adult") OR (MH "Students, College") OR (MH "Students, Undergraduate") OR ((((TI college OR AB college) OR (TI undergraduate* OR AB undergraduate*) OR (TI postsecondary OR AB postsecondary) OR (TI "post secondary" OR AB "post secondary") OR (TI post-secondary OR AB post-secondary) OR (TI university OR AB university)) AND ((TI age OR AB age) OR (TI aged OR AB aged) OR (TI student* OR AB student*))) OR (((TI young OR AB young) OR (TI emerging OR AB emerging)) AND ((TI adult* OR AB adult*) OR (TI adulthood OR AB adulthood) OR (TI people OR AB people) OR (TI person OR AB person))) OR (TI undergraduate* OR AB undergraduate*)) |
| Substance use AND Technology AND young adults | 4 | S1 AND S2 AND S3 |
| RCTs (Cochrane) | 5 | ( MH ( randomized controlled trials OR double‐blind studies OR single‐blind studies OR random assignment OR pretest‐posttest design OR cluster sample ) OR TI ( randomised OR randomized ) OR AB random* OR TI trial OR ( (MH (sample size) AND AB (assigned OR allocated OR control)) ) OR MH ( placebos OR crossover design OR comparative studies ) OR AB ( (control W5 group) OR (cluster W3 RCT) OR PT (randomized controlled trial)) ) NOT ( ( MH animals+ OR MH (animal studies) OR TI (animal model*) ) NOT MH (human) ) |
|  | 6 | S4 AND S5 |

**PsycINFO**

| **Subject** | **Search #** | **Search Strategy** |
| --- | --- | --- |
| Substance use | 1 | DE "Substance Related and Addictive Disorders" OR DE "Substance Use Disorder" OR DE "Alcohol Use Disorder" OR DE "Cannabis Use Disorder" OR DE "Drug Abuse" OR DE "Drug Dependency" OR DE "Inhalant Abuse" OR DE "Opioid Use Disorder" OR DE "Cannabis Use Disorder" OR DE "Alcohol Use Disorder" OR DE "Alcohol Abuse" OR DE "Alcohol Intoxication" OR DE "Alcohol Withdrawal" OR DE "Alcoholism" OR DE "Underage Drinking" OR DE "Acute Alcoholic Intoxication" OR DE "Chronic Alcoholic Intoxication" OR DE "Alcohol Drinking Attitudes" OR DE "Binge Drinking" OR DE "Social Drinking" OR DE "Alcohol Drinking Patterns" OR DE "Drug Overdoses" OR DE "Intravenous Drug Usage" OR DE "Drug Usage" OR DE "Marijuana Usage" OR DE "Prescription Drug Misuse" OR DE "Drug Seeking" OR DE "Prescription Drug Misuse" OR DE "Drug Addiction" OR DE "Drug Usage Attitudes" OR DE "Polydrug Abuse" OR (((TI substance OR AB substance) OR (TI alcohol* OR AB alcohol*) OR (TI tobacco OR AB tobacco) OR (TI drug OR AB drug) OR (TI smok* OR AB smok*)) AND ((TI use OR AB use) OR (TI usage OR AB usage) OR (TI user OR AB user) OR (TI misuse* OR AB misuse*) OR (TI initiat* OR AB initiat*) OR (TI abus* OR AB abus*) OR (TI problem* OR AB problem*) OR (TI heavy OR AB heavy) OR (TI binge OR AB binge) OR (TI disorder* OR AB disorder*) OR (TI dependen* OR AB dependen*) OR (TI frequen* OR AB frequen*))) |
| Technology | 2 | DE "Robotics" OR DE "Avatars" OR DE "Human Robot Interaction" OR DE "Intelligent Agents" OR DE "Social Robotics" OR DE "Internet" OR DE "Blog" OR DE "Online Social Networks" OR DE "Smartphones" OR DE "Mobile Applications" OR DE "Social Media" OR DE "Telephone Systems" OR DE "Mobile Phones" OR DE "Smartphones" OR DE "Computers" OR DE "Analog Computers" OR DE "Cloud Computing" OR DE "Computer Games" OR DE "Computer Peripheral Devices" OR DE "Computer Software" OR DE "Digital Computers" OR DE "Microcomputers" OR DE "Mobile Devices" OR DE "Digital Interventions" OR DE "Wireless Technologies" OR DE "Telemetry" OR DE "Mobile Technology" OR DE "Mobile Applications" OR DE "Wearable Devices" OR DE "Human Technology Interaction" OR DE "Human Computer Interaction" OR DE "Screen Time" OR DE "Technology" OR DE "Assistive Technology" OR DE "Automation" OR DE "Information and Communication Technology" OR ((TI digital* OR AB digital*) OR (TI technol* OR AB technol*) OR (TI sensor* OR AB sensor*) OR (TI robot* OR AB robot*) OR (TI internet* OR AB internet*) OR (TI "social media" OR AB "social media") OR (TI smartphone* OR AB smartphone*) OR (TI "smart phone*" OR AB "smart phone*") OR (TI telephone* OR AB telephone*) OR (TI phone* OR AB phone*) OR (TI online OR AB online) OR (TI ipad* OR AB ipad*) OR (TI computer* OR AB computer*) OR (TI electronic* OR AB electronic*) OR (TI Web OR AB Web)) |
| Young adults or college students | 3 | DE "Emerging Adulthood" OR DE "College Students" OR DE "Community College Students" OR DE "Education Students" OR DE "Junior College Students" OR DE "Nursing Students" OR DE "ROTC Students" OR ((((TI college OR AB college) OR (TI undergraduate* OR AB undergraduate*) OR (TI postsecondary OR AB postsecondary) OR (TI "post secondary" OR AB "post secondary") OR (TI post-secondary OR AB post-secondary) OR (TI university OR AB university)) AND ((TI age OR AB age) OR (TI aged OR AB aged) OR (TI student* OR AB student*))) OR (((TI young OR AB young) OR (TI emerging OR AB emerging)) AND ((TI adult* OR AB adult*) OR (TI adulthood OR AB adulthood) OR (TI people OR AB people) OR (TI person OR AB person))) OR (TI undergraduate* OR AB undergraduate*)) |
| substance use and technology and young adults | 4 | S1 AND S2 AND S3 |
| RCTs | 5 | DE "Clinical Trials" OR DE "Randomized Controlled Trials" OR (DE "Placebo") OR ((TI random* OR AB random*) OR (TI sham OR AB sham) OR (TI placebo* OR AB placebo*) OR (((TI singl* OR AB singl*) OR (TI doubl* OR AB doubl*)) W1 ((TI blind* OR AB blind*) OR (TI dumm* OR AB dumm*) OR (TI mask* OR AB mask*))) OR (((TI tripl* OR AB tripl*) OR (TI trebl* OR AB trebl*)) W1 ((TI blind* OR AB blind*) OR (TI dumm* OR AB dumm*) OR (TI mask* OR AB mask*))) OR ((TI control* OR AB control*) N3 ((TI study OR AB study) OR (TI studies OR AB studies) OR (TI trial* OR AB trial*) OR (TI group* OR AB group*))) OR (TI Nonrandom* OR AB Nonrandom*) OR (TI "non random*" OR AB "non random*") OR (TI non-random* OR AB non-random*) OR (TI quasi-random* OR AB quasi-random*) OR (TI quasirandom* OR AB quasirandom*) OR (TI allocated OR AB allocated) OR (((TI "open label" OR AB "open label") OR (TI open-label OR AB open-label)) N5 ((TI study OR AB study) OR (TI studies OR AB studies) OR (TI trial* OR AB trial*))) OR (((TI equivalence OR AB equivalence) OR (TI superiority OR AB superiority) OR (TI non-inferiority OR AB non-inferiority) OR (TI noninferiority OR AB noninferiority)) N3 ((TI study OR AB study) OR (TI studies OR AB studies) OR (TI trial* OR AB trial*))) OR (((TI pragmatic OR AB pragmatic) OR (TI practical OR AB practical)) N3 (TI trial* OR AB trial*)) OR (((TI quasiexperimental OR AB quasiexperimental) OR (TI quasi-experimental OR AB quasi-experimental)) N3 ((TI study OR AB study) OR (TI studies OR AB studies) OR (TI trial* OR AB trial*))) OR ((TI phase OR AB phase) N3 ((TI III OR AB III) OR (TI 3 OR AB 3)) N3 ((TI study OR AB study) OR (TI studies OR AB studies) OR (TI trial* OR AB trial*)))) |
| w/ RCTs | 6 | S4 AND S5 |

**Cochrane Library**

| **Search #** | **Search Strategy** |
| --- | --- |
| 1 | [mh "Substance-Related Disorders"] OR [mh "Alcohol-Related Disorders"] OR [mh "Alcoholic Intoxication"] OR [mh Alcoholism] OR [mh "Binge Drinking"] OR [mh "Amphetamine-Related Disorders"] OR [mh "Cocaine-Related Disorders"] OR [mh "Drug Overdose"] OR [mh "Inhalant Abuse"] OR [mh "Marijuana Abuse"] OR [mh "Narcotic-Related Disorders"] OR [mh "Opioid-Related Disorders"] OR [mh "Phencyclidine Abuse"] OR [mh "Substance Abuse, Intravenous"] OR [mh "Substance Abuse, Oral"] OR ((substance:ti,ab OR alcohol*:ti,ab OR tobacco:ti,ab OR drug:ti,ab OR smok*:ti,ab) AND (use:ti,ab OR usage:ti,ab OR user:ti,ab OR misuse*:ti,ab OR initiat*:ti,ab OR abus*:ti,ab OR problem*:ti,ab OR heavy:ti,ab OR binge:ti,ab OR disorder*:ti,ab OR dependen*:ti,ab OR frequen*:ti,ab)) |
| 2 | (digital*:ti,ab OR technol*:ti,ab OR sensor*:ti,ab OR robot*:ti,ab OR internet*:ti,ab OR "social media":ti,ab OR smartphone*:ti,ab OR ("smart" NEXT phone*):ti,ab OR telephone*:ti,ab OR phone*:ti,ab OR online:ti,ab OR ipad*:ti,ab OR computer*:ti,ab OR electronic*:ti,ab OR Web:ti,ab) OR [mh ROBOTICS] OR [mh INTERNET] OR [mh "Social Media"] OR [mh TELEPHONE] OR [mh Smartphone] OR [mh "Computers, Handheld"] OR [mh COMPUTERS] |
| 3 | [mh "young adult"] OR (((college:ti,ab OR undergraduate*:ti,ab OR postsecondary:ti,ab OR "post secondary":ti,ab OR post-secondary:ti,ab OR university:ti,ab) AND (age:ti,ab OR aged:ti,ab OR student*:ti,ab)) OR ((young:ti,ab OR emerging:ti,ab) AND (adult*:ti,ab OR adulthood:ti,ab OR people:ti,ab OR person:ti,ab)) OR undergraduate*:ti,ab) |
| 4 | #1 AND #2 AND #3 |

**ProQuest Dissertations & Theses Global**

| **Subject** | **Search #** | **Search Strategy** |
| --- | --- | --- |
| Substance Use | 1 | ((TI,AB(substance) OR TI,AB(alcohol*) OR TI,AB(tobacco) OR TI,AB(drug) OR TI,AB(smok*)) AND (TI,AB(use) OR TI,AB(usage) OR TI,AB(user) OR TI,AB(misuse*) OR TI,AB(initiat*) OR TI,AB(abus*) OR TI,AB(problem*) OR TI,AB(heavy) OR TI,AB(binge) OR TI,AB(disorder*) OR TI,AB(dependen*) OR TI,AB(frequen*))) |
| Technology | 2 | (TI,AB(digital*) OR TI,AB(technol*) OR TI,AB(sensor*) OR TI,AB(robot*) OR TI,AB(internet*) OR TI,AB("social media") OR TI,AB(smartphone*) OR TI,AB("smart phone*") OR TI,AB(telephone*) OR TI,AB(phone*) OR TI,AB(online) OR TI,AB(ipad*) OR TI,AB(computer*) OR TI,AB(electronic*) OR TI,AB(Web)) |
| young adults or college students | 3 | (((TI,AB(college) OR TI,AB(undergraduate*) OR TI,AB(postsecondary) OR TI,AB("post secondary") OR TI,AB(post-secondary) OR TI,AB(university)) AND (TI,AB(age) OR TI,AB(aged) OR TI,AB(student*))) OR ((TI,AB(young) OR TI,AB(emerging)) AND (TI,AB(adult*) OR TI,AB(adulthood) OR TI,AB(people) OR TI,AB(person))) OR TI,AB(undergraduate*)) |
|  | 4 | mainsubject.Exact("placebo effect" OR "medical treatment" OR "clinical outcomes" OR "clinical trials") OR placebo* OR random* OR "comparative stud*" OR clinical NEAR/3 trial* OR research NEAR/3 design OR evaluat* NEAR/3 stud* OR prospectiv* NEAR/3 stud* OR (singl* OR doubl* OR trebl* OR tripl*) NEAR/3 (blind* OR mask*) |
|  | 5 | S1 AND S2 AND S3 |
|  | 5 | S4 AND S5 |

**SocINDEX**

| **Subject** | **Search #** | **Search Strategy** |
| --- | --- | --- |
| Substance Use | 1 | DE "INTRAVENOUS drug abuse" OR DE "SUBSTANCE abuse" OR DE "ALCOHOLISM" OR DE "CAFFEINE habit" OR DE "DRUG abuse" OR DE "AMPHETAMINE abuse" OR DE "COCAINE abuse" OR DE "DRUG addiction" OR DE "HEROIN abuse" OR DE "INTRAVENOUS drug abuse" OR DE "MARIJUANA abuse" OR DE "MEDICATION abuse" OR DE "SEDATIVE abuse" OR DE "SUBSTANCE-induced disorders" OR DE "YOUNG adults -- Substance use" OR DE "WOMEN -- Substance use" OR DE "STUDENTS -- Substance use" OR DE "MEN -- Substance use" OR DE "COLLEGE students -- Substance use" OR DE "ALCOHOL drinking" OR DE "BINGE drinking" OR DE "STUDENTS -- Alcohol use" OR DE "ALCOHOLIC intoxication" OR DE "YOUNG adults -- Alcohol use" OR DE "DRINKING of alcoholic beverages & psychology" OR DE "NARCOTICS -- Overdose" OR DE "INHALANT abuse" OR DE "AEROSOL sniffing" OR DE "GLUE sniffing" OR DE "PAINT sniffing" OR DE "DRUG addicts" OR (((TI substance OR AB substance) OR (TI alcohol* OR AB alcohol*) OR (TI tobacco OR AB tobacco) OR (TI drug OR AB drug) OR (TI smok* OR AB smok*)) AND ((TI use OR AB use) OR (TI usage OR AB usage) OR (TI user OR AB user) OR (TI misuse* OR AB misuse*) OR (TI initiat* OR AB initiat*) OR (TI abus* OR AB abus*) OR (TI problem* OR AB problem*) OR (TI heavy OR AB heavy) OR (TI binge OR AB binge) OR (TI disorder* OR AB disorder*) OR (TI dependen* OR AB dependen*) OR (TI frequen* OR AB frequen*))) |
|  | 2 | DE "INTERNET" OR DE "VIRTUAL communities" OR DE "SOCIAL media" OR DE "BLOGS" OR DE "COMPUTER bulletin boards" OR DE "ONLINE chat" OR DE "CELL phones" OR DE "TELEPHONES" OR DE "COMPUTERS" OR DE "PERSONAL computers" OR DE "TECHNOLOGY" OR DE "APPROPRIATE technology" OR DE "COMMUNICATION & technology" OR DE "EDUCATIONAL technology" OR DE "ELECTRONICS -- Social aspects" OR DE "INFORMATION technology" OR DE "MEDICAL technology" OR ((TI digital* OR AB digital*) OR (TI technol* OR AB technol*) OR (TI sensor* OR AB sensor*) OR (TI robot* OR AB robot*) OR (TI internet* OR AB internet*) OR (TI "social media" OR AB "social media") OR (TI smartphone* OR AB smartphone*) OR (TI "smart phone*" OR AB "smart phone*") OR (TI telephone* OR AB telephone*) OR (TI phone* OR AB phone*) OR (TI online OR AB online) OR (TI ipad* OR AB ipad*) OR (TI computer* OR AB computer*) OR (TI electronic* OR AB electronic*) OR (TI Web OR AB Web)) |
|  | 3 | ((((TI college OR AB college) OR (TI undergraduate* OR AB undergraduate*) OR (TI postsecondary OR AB postsecondary) OR (TI "post secondary" OR AB "post secondary") OR (TI post-secondary OR AB post-secondary) OR (TI university OR AB university)) AND ((TI age OR AB age) OR (TI aged OR AB aged) OR (TI student* OR AB student*))) OR (((TI young OR AB young) OR (TI emerging OR AB emerging)) AND ((TI adult* OR AB adult*) OR (TI adulthood OR AB adulthood) OR (TI people OR AB people) OR (TI person OR AB person))) OR (TI undergraduate* OR AB undergraduate*)) OR DE "COLLEGE students" OR DE "UNDERGRADUATES" OR DE "VOCATIONAL school students" OR DE "YOUNG adults" OR DE "AFRICAN American young adults" OR DE "LGBTQ+ young adults" OR DE "YOUNG men" OR DE "YOUNG women" OR DE "YUPPIES" |
|  | 4 | S1 AND S2 AND S3 |
|  | 5 | (DE "RANDOMIZED controlled trials") OR (DE "BLIND experiment") OR TI ( randomised OR randomized ) OR AB random* OR TI trial OR ( ((TI (sample size) OR AB (sample size)) AND AB (assigned OR allocated OR control)) ) OR TI ( placebos OR crossover design OR comparative studies ) OR AB ( placebos OR crossover design OR comparative studies ) OR AB ( (control W5 group) OR (cluster W3 RCT) OR AB (randomized controlled trial)) ) |
|  | 6 | S4 AND S5 |
