# Supplementary material for: Technology-Based Substance Use Interventions for Emerging Adults and College Students: A Systematic Review and Meta-Analysis
Source: Int J Ment Health Addict. 2024 Dec 26;24(1):603–22. doi: 10.1007/s11469-024-01433-7 (PMC12456661; doi:10.1007/s11469-024-01433-7)
Supplement: Supplementary file 3 — Supplementary file3 (DOCX 15.4 KB) [file 11469_2024_1433_MOESM3_ESM.docx]

Appendix C Sensitivity analyses results (meta-regression for estimating the overall effect size)

|  | b | SE | t | df | p | 95% CI | |
| --- | --- | --- | --- | --- | --- | --- | --- |
| With outliers | | | | | | | |
| Intercept | **0.24** | 0.03 | 9.55 | 124.55 | <.001 | 0.19 | 0.29 |
| With outliers + Assessment timing adjusted | | | | | | | |
| Intercept | **0.25** | 0.03 | 7.94 | 35.39 | <.001 | 0.19 | 0.31 |
| Assessment timing | -0.001 | 0.001 | -0.77 | 3.31 | 0.49 | -0.004 | 0.002 |
| Without outliers | | | | | | | |
| Intercept | **0.22** | 0.02 | 10.45 | 122.31 | <.001 | 0.18 | 0.26 |
| Without outliers + Assessment timing adjusted | | | | | | | |
| Intercept | **0.23** | 0.03 | 9.27 | 34.68 | <.001 | 0.18 | 0.28 |
| Assessment timing | -0.001 | 0.001 | -1.35 | 3.16 | 0.265 | -0.003 | 0.001 |

Note. Assessment timing refers to the timing of the follow-up assessments such as four weeks post baseline.
