# Supplementary material for: Technology-Based Substance Use Interventions for Emerging Adults and College Students: A Systematic Review and Meta-Analysis
Source: Int J Ment Health Addict. 2024 Dec 26;24(1):603–22. doi: 10.1007/s11469-024-01433-7 (PMC12456661; doi:10.1007/s11469-024-01433-7)
Supplement: Supplementary file 4 — Supplementary file4 (DOCX 193 KB) [file 11469_2024_1433_MOESM4_ESM.docx]

Appendix D. Characteristics of studies under review (N=130).

| **Author, Year; Country; Sample Size (N)** | **Participant Characteristics (Young adult/college student; Mean Age; % Female; Race/Ethnicity; and Any Special Characteristic); Setting** | **Technology-Based Intervention (description; communication type; human contact; length/frequency)** | **Control** | **Outcomes of Interest (measures)** |
| --- | --- | --- | --- | --- |
| (Alfonso et al., 2013); USA; N=173 | College students; Avg age 18.8; 43% female; 4% Asian, 5% Black, 8% Latinx, 81% White, 2% other | Web-based/computer intervention; e-CHUG: an online, self-administered, personalized normative feedback intervention (PNFI) that reports on various aspects of alcohol consumption; 1 way communication; no human contact; length NR; frequency NR | Non-tech intervention: BASICS, an individualized alcohol assessment and feedback intervention for college students that is designed for delivery in two 50-minute sessions and includes cognitive-behavioral skills training, motivational enhancement and personalized feedback | Average blood alcohol content (BAC); Peak BAC; Peak number of drinks consumed in one sitting; Negative alcohol-related consequences (Rutgers Alcohol Problem Index) |
| (Andersson, 2015); Sweden; N=1678 | College students; Avg age 23.2; 41% female; Race/ethnicity NR | Two Web-based/computer interventions; Intervention content included personalized normative feedback and protective behavioral strategies aimed at reducing intoxication; two interventions with largely the same but slightly different content/delivery order; One way communication; No human contact; Length NR; Frequency NR | No treatment/waitlist control | DDQ mean BAC (16-item Daily Drinking Questionnaire [DDQ] and estimations of mean BAC on a weekly basis); Quantity and frequency of drinking (quantity and frequency scores from DDQ); alcohol consumption, signs of alcohol dependence, and alcohol-related harm during the past year (Alcohol Use Disorders Identification Test [AUDIT]) |
| (Andrade et al., 2024)  Denmark, N= 931 | College students; Avg age 23.8; 63% female; Race/ethnicity NR | a web-based program aimed at reducing alcohol consumption among university students. It incorporated a mix of nudging techniques, based on the theory of planned behavior, to alter students' drinking habits without enforcing prohibitive measures; Two-way communication; No human contact; Length 8 weeks; Frequency 3 sessions/week | Treatment as usual | # of times drinking alcohol in past month, # of binge drinking in past month, # of drinks consumed on typical drinking day, past month |
| (Arazan et al., 2023); USA; N=1064 | College students; Avg age NR; 59.5% female; 2% Asian, 3% Black, 16% Latinx, 62% White, 17% other | Email intervention; e-card with strategies to minimize harm associated with celebratory drinking; One way communication; No human contact; Length NR; Frequency Nr | No treatment/waitlist control | Summed scale of negative outcomes (22 statements) (survey created in part using questions from the Rutgers Alcohol Problem  Index [RAPI]) |
| (Barnett et al., 2007); USA N=225 | College students; Avg age 18.8; 51.1% female; 14% Asian, 2% Black, 10% Latinx, 74% White, 0% Other | Web-based/computer intervention; an individual session with Alcohol 101: the program features a “virtual party” that has a number of different “rooms”, starting with a virtual bar in which participants can observe the effects of gender, weight, drink type, and speed of consumption on BAC; One way communication; No human contact; Length NR; Frequency NR | Non-tech intervention: brief motivational intervention | Alcohol problems (Young Adult Alcohol Problems Screening Test); Avg estimated BAC (Timeline Follow Back, [TLFB]); Average number of drinks per day (TLFB); Number of heavy drinking days (TLFB); Number of drinking days (TLFB); Avg number of drinks per drinking day (TLFB) |
| (Bedendo et al., 2024)  Brazil; N= 931 | College students; Avg age 24.4; 66.4% female; Race/ethnicity NR | web-based Personalized Normative Feedback (PNF) to address and reduce alcohol consumption, using two versions: Single PNF and Multiple PNF; One-way communication; No human contact; Length 24 weeks; single session | Assessment only | Typical number of drinks per week, Maximum number of drinks per week, Drinking frequency (days/week), Number of consequences |
| (Bendtsen et al., 2012); Sweden; N=5227 | College students; Avg age NR; 52.1% female; Race/ethnicity NR | Email (+ computer/web) intervention; personalized feedback on alcohol use, and general information on alcohol use and its consequences; One way communication; No human contact; Length NR; Frequency NR | No treatment/waitlist control | Frequency of monthly heavy episodic drinking (AUDIT); Dependence score (AUDIT); Problem score (AUDIT); Total score (AUDIT) |
| (Bendtsen et al., 2015); Sweden; N=1605 | College students; Avg age NR; 49.3% female; Race/ethnicity NR | Web-based/computer intervention; computer-based comprehensive normative feedback on alcohol use; One way communication; No human contact; Length NR; Frequency NR | No treatment/waitlist control | Highest estimated blood alcohol concentration (eBAC); motivation to change; Frequency of drinking (days/week); Frequency of heavy episode drinking occasions; Number of drinks per drinking day; Weekly alcohol consumption (g/week) (the sum of alcohol consumed in standard drinks for each of the 7 days in a typical week); Proportion drinking above national guidelines |
| (Bernstein et al., 2018);USA; N=200 | College students; Avg age NR; 69% female; 3% Asian, 2% Black, 9% Latinx, 86% White, 0% other | Text-based; text-based intervention before planned birthday celebration, includes normative feedback; text/MMS; two way communication; no human contact; .5 minutes length; frequency NR | No treatment/waitlist control | 21^st^ birthday drinking norms (“How many drinks do you think a typical University of Rhode Island student of your gender consumes on his/her 21^st^ birthday?” ) |
| (Bertholet et al., 2015b) study 1; Switzerland; N=896 | General young adult population; Avg Age 21.0; 0% female; Race/ethnicity NR | Web-based/computer intervention; personalized feedback on alcohol use, and general information on alcohol use and its consequences; one way communication; no human contact; length NR; frequency NR | No treatment/waitlist control | Monthly binge drinking prevalence (defined as 6 or more drinks in one occasion); Number of alcohol-related consequences (from 12 alcohol related consequences over the past 6 months); AUDIT score; Number of drinks/week (using quantity/frequency survey questions on weekly alcohol consumption) |
| (Bertholet et al., 2015a) study 2; Switzerland; N=21 | General young adult population ; Avg Age 20.8; 0% female ; Race/ ethnicity NR | Web-based/computer intervention; Normative feedback on alcohol use; one way communication; no human contact; length NR; frequency NR | No treatment/waitlist control | Binge drinking prevalence; Alcohol related consequences; AUDIT score; Number of drinks per week |
| (Bertholet et al., 2018); Switzerland; N=737 | General young adult population; Avg Age 20.7; 0% female; Race/ethnicity NR | Web-based/computer intervention; Normative feedback on alcohol use; one way communication; no human contact; length NR; frequency NR | No treatment/waitlist control | Number of drinks/week; Monthly or more binge drinking prevalence |
| (Bertholet et al., 2023); Switzerland; N= 1770 | College students; Avg age 22.4; 54.1% female; Race/ethnicity NR | Smartphone-based app designed to help university students with unhealthy alcohol use. The app offered personalized feedback and self-monitoring tools; Two-way communication; No human contact; Length 12 weeks; Frequency 1 session/week | No treatment | Number of standard drinks per week, Number of heavy drinking days (past 30 days), Max number of drinks on one occasion (past 30 days), Alcohol related consequences, Academic performance |
| (Bewick et al., 2008); UK; N=506 | College students; Avg Age 21.3; 69% female; Race/ethnicity NR | Web-based intervention; Website based personalize feedback on alcohol consumption and social norms; one way communication; no human contact; Length 12 minutes, frequency NR | No treatment/waitlist control | Units per occasion; Units per week |
| (Bewick et al., 2010); UK; N=1112 | College students; Avg age 21.5; 73% female; 92% White, 8% other | Web-based/computer intervention; Unitcheck: fully automated, personalized feedback on alcohol consumption; One way communication; No human contact; Length NR; Frequency NR | No treatment/waitlist control | Total units consumed over the last week (7-day retrospective drinking diary for the previous week); Avg units consumed per drinking occasion over the last week (7-day retrospective drinking diary) |
| (Bewick et al., 2013); UK; N=1618 | College students; Avg Age 20.8; 69% female; 88% White, 12% other | Web-based/computer intervention; Unit-check: fully automated personalized-feedback on alcohol consumption; one way communication; No human contact; Length 15 minutes, frequency NR | No treatment/waitlist control | Units consumed on average drinking occasion (drinking diary); Units consumed over the previous week (drinking diary) |
| (Bonar et al., 2021); USA; N=63 | General young adult population; Avg age 21.7; 66.7% female; 0% Asian, 52% Black, 8% Latinx, 38% White, 2% other | App + web intervention; BI counseling session and daily booster messages tailored to substance use motives sent via a mobile app; Two way communication; Some human contact; Length 4 minutes; Frequency NR | Non-tech intervention: 5 minute health resource brochure | Total past 2-month alcohol consequences (semi-structured interview); Total past 2-month substance use consequences; Substance use days/month (including cannabis and alcohol); Total alcohol consumption (drinks/ month); Total cannabis consumption (joints/ month) (semi-structured interview) |
| (Bonar et al., 2022); USA; N=149 | General young adult population; Avg age 21; 55.7% female; 0% Asian, 17% Black, 17% Latinx, 67% White, 0% other | Web-based intervention; online content from "e-coaches" themed around cannabis use; One way communication; No human contact; Length 8 minutes; Frequency NR | No treatment attention control | total quantity vaped (TLFB for all below); past 30-day use of cannabis; number of times used per day; quantity consumed per day; total quantity of edibles; total days used edibles; total times used edibles; total quantity dabbed; total days vaped; total alcohol + cannabis co-use days; total alcohol use days; total days dabbed; total cannabis use days; total days smoked; total quantity smoked; total times vaped; total alcohol drinks; total times smoked; total times dabbed; total times used cannabis; total quantity vaped; total quantity smoked |
| (Bonar et al., 2024)  ; USA; N=102 | General young adult population; Avg age 20.9; 49% female; 2.95% Asian, 11.75% Black, 23.55% Latinx, 61.75 White, 0% other | 8-week social media-based program delivered via Snapchat, targeting high-intensity drinking (HID) in emerging adults; Two-way communication; Some human contact; Length 8 weeks; Frequency 8 sessions/week | Psychoeducational website referral | past month HID (high intensity drinking) frequency, past 2 month AUDIT-C score, total drinks in past 7 days, total HID days in past 7 days, total typical weekly drinks, total typical # HID days in a week, past 2 month alcohol consequences count, past 2 month alcohol impaired driving, past 2 month cannabis frequency, past 2 month cannabis impaired driving |
| (Borsari et al., 2014); USA; N=57 | College students; Avg age 19.1; 39% female; 96% White, 4% other | Email+phone intervention; pBMI sessions providing personalized feedback emailed to the participant; Two way communication; Some human contact; Length NR; Frequency NR | No treatment/waitlist control | Alcohol related problems (Brief Young Adult Alcohol Consequences Questionnaire [BYAACQ]) |
| (Braitman & Lau‐Barraco, 2018); USA; N=537 | College students; Avg age 19.7; 67.4% female; 4% Asian, 37% Black, 10% Latinx, 49% White, 0% other | Email (+computer/web) intervention; Alcohol 101 PlusTM for 60 minutes: an online intervention on alcohol education, with additional email personalized feedback; One way communication; No human contact; Length NR; Frequency NR | Treatment as usual: Participants in the control condition navigated through a general health education session developed by Lilly for Better Health. It offers interactive resources such as health screeners, quizzes, knowledge builders, and self-management tools to help individuals prevent or manage health conditions. | Typical BAC (Modified Daily Drinking Questionnaire [DDQ]); Peak BAC (Modified DDQ); Frequency (Modified DDQ); Peak drinks (Modified DDQ); Problems (YAACQ); Quantity (Modified DDQ) |
| (Bryant, 2009); USA; N=191 | College students; Avg Age 18.7; 76% female; 3% Asian, 9% Black, 0% Latinx, 82% White, 6% other | Email intervention; BASICS: personalized feedback emailed to participants; one way communication; No human contact; Length NR; Frequency NR | Treatment as usual: generic as feedback | Frequency (Days/Week) (Perceived Peer Alcohol Use, or PPAU); Quantity (Drinks/occasion) (PPAU); # of days felt drunk from alcohol use in past 30 days; # of days felt high from alcohol use in past 30 days |
| (Buckner et al., 2020); USA’ N=63 | College students; Avg age 19.1; 83.1% female; Race/ethnicity NR | Web-based intervention; online personalized feedback intervention; One way communication; No human contact; Length NR; Frequency NR | No treatment/waitlist control | # of drinking related problems (RAPI); heavy drinking quantity (DDQ); typical drinking quantity (DDQ); past 2-week cannabis frequency (TLFB); # of cannabis-related problems (Brief Marijuana Consequences Questionnaire) |
| (Butler & Correia, 2009); USA; N=84 | College students; Avg age 20.5; % female NR; 92% White, 8% other | Computer intervention: Computer based personalized feedback; One way communication; No human contact; Length NR; Frequency NR | 1 Non-tech intervention: The specific content included in  the feedback was identical in both the face-to-face and computerized feedback condition. Participants in the face-to-face feedback condition met with a graduate clinician to review a printed feedback form using a motivational interviewing approach.  2 No treatment/waitlist control | Drinking occasions (DDQ); Binge episodes (DDQ); Drinks per week (DDQ); RAPI scores (RAPI) |
| (Cameron et al., 2015); UK; N=2621 | College students; Avg age 18.8; 55.3% female; 14% Asian, 2% Black, 0% Latinx, 77% White, 6% other | Web-based/computer intervention; self-affirmation manipulation + health behavior modules; One way communication; No human contact; Length NR; Frequency NR | No treatment/waitlist control | Recreational drug use (3 cm long hair samples were liquefied and analyzed for biochemical markers); alcohol related problems (AUDIT score); # of days binge drinking in previous 7 days (retrospective 7-day recall drinking diary); units in the last 7 days (retrospective 7-day recall drinking diary); alcohol consumption (fatty acid ethyl esters, a biochemical marker); recreational drug use (single sample count method) |
| (Carey et al., 2009); USA; N=198 | College students; Avg age 19.2; 46% female; 91% White, 9% other | Web-based/computer intervention; Interactive program that explores alcohol-related issues in a “virtual campus”; One way communication; Some human contact; Length NR; Frequency NR | Treatment as usual: brief motivational interviewing | Peak BAC; average number of drinks consumed on a typical drinking day; the number of standard drinks consumed in a typical week; maximum number of drinks consumed in one day; hours spent drinking on that day; Drinking during a typical week (using two 7-day grids); Heaviest drinking week in the month before the sanction event (using two 7-day grids); maximum number of drinks consumed in a single day; number of hours spent drinking on that day; Typical drinks per occasion (modified DDQ); Binge frequency in last month (modified DDQ); Drinks per typical week in a month (modified DDQ); Drinks in the heaviest drinking week of the last month (modified DDQ); Alcohol problems (RAPI) |
| (Carey et al., 2011); USA; N=677 | College students; Avg age 19.0; 36% female; Race/ethnicity NR | Web-based/computer interventions; TBI a) Alcohol 101 Plus CD-ROM program and TBI b) Alcohol Edu for Sanctions computer-based program; Two way communication; No human contact; Length NR; Frequency NR | 1 Treatment as usual (brief motivational interviewing as active control)  2 Waitlist control | Alcohol related consequences (RAPI total score); Drinks per heaviest week (Two 7-day grids, patterned after the DDQ); drinks consumed in a typical week; drinks consumed in the heaviest week in the last month |
| (Carey et al., 2024)  ; USA; N=484 | College students; Avg age 18.7; 44% female; 10.4% Asian, 2.3% Black, 10.6% Latinx, 76.6% White, 0% other | Computer-based Personalized Feedback Intervention (PFI) combined with a Self-Affirmation (SA) exercise to reduce drinking among college students mandated to attend an alcohol education program; One-way communication; No human contact; Length 52 weeks; Single session | Non tech intervention: Attention control (no-affirmation) | Typical drinking quantity, Peak drinking quantity, Binge frequency, Alcohol-related consequences |
| (Chavez & Palfai, 2021); USA; N=30 | College students; Avg age 18.9; 70% female; 30% Asian, 3% Black, 7% Latinx, 60% White, 0% other | App + web intervention; personalized feedback on alcohol use, and general information on alcohol use and its consequences; Two way communication; No human contact; Length 5 minutes; Frequency 1 text/week | No treatment/waitlist control | Past-month heavy drinking episode frequency; drinking consequences (BYAACQ total score for the past month) |
| (Chiauzzi et al., 2005); USA; N=265 | College students; Avg age 19.9; 54% female; 11% Asian, 3% Black, 8% Latinx, 73% White, 5% other | Web-based intervention; An interactive website that offers motivational feedback to encourage risk reduction behaviors; One way communication; No human contact; Length 4 min; Frequency 1 | Treatment as usual: General educational content (Alcohol and You) was intended to provide a comparison with the educational content found at many websites | Average consumption/drinking day; Peak consumption (Maximum number of drinks consumed/drinking day); Peak consumption (binge drinking days/week); Frequency of consumption (drinking days/week); Alcohol composite score (sum of Z scores); Peak consumption during special occasion drinking over past 3 months; Quantity of consumption (drinks/week); Total consumption during special occasion past 3 months |
| (Choi et al., 2023)  ; USA; N=283 | College students; Avg age 18.25; 82% female; 19% Asian, 17% Black, 7% Latinx, 46% White, 11% other | An online Personalized Feedback Program (PFP) targeting college students' alcohol and cannabis use; One-way communication; No human contact; Length 12 weeks; Single session | Non tech intervention: University resource information only | Alcohol use, Cannabis use |
| (Christoff & Boerngen-Lacerda, 2015); Brazil; N=458 | College students; Avg age 24; 57.7% female; Race/ethnicity NR | Web-based intervention; simple and rapid interactive website; One way communication; No human contact; Length NR; Frequency NR | Treatment as usual: (ASSIST/MBIi); No treatment/waitlist control: assessment only control | Low Risk Limit Scores (for alcohol, tobacco, cannabis, summation of other drugs, and total substance involvement) indicating low, moderate, or high risk for substance involvement (Alcohol, Smoking, and Substance Involvement Screening Test [ASSIST], ASSISTc, or ASSISTi) |
| (Chun et al., 2022); South Korea; N=183 | College students; Avg age NR; 100% female; Race/ethnicity NR | Web-based intervention; eight-week web-based intervention consisted of assessment, normative feedback, and motivational planning; Two way communication; No human contact; Length 8 minutes; Frequency NR | No treatment/waitlist control | Drinking behavior (AUDIT-C) |
| (Collins et al., 2014); USA; N=724 | College students; Avg age 20.8; 56% female; 18% Asian, 1% Black, 7% Latinx, 67% White, 8% other | Web-based/computer intervention; personalized feedback on alcohol use, and general information on alcohol use and its consequences; One way communication; No human contact; Length NR; Frequency NR | No treatment/waitlist control: assessment only control | Alcohol problems (RAPI); drinking frequency (The Frequency–Quantity [F-Q] questionnaire); alcohol quantity (TLFB) |
| (Conner et al., 2024)  ; USA; N=781 | College students; Avg age 21.7; 41.2% female; Race/ethnicity NR | A web-based program called Cannabis eCHECKUP TO GO, which provided personalized normative feedback (PNF) and protective behavioral strategies (PBS) to help college students reduce cannabis use; One-way communication; No human contact; Length 4 weeks; Single session | Non tech intervention: Healthy stress management information | Cannabis use |
| (Côté et al., 2018); Canada; N=588 | General young adult population; Avg age 19.8; 54.3% female; Race/ethnicity NR | Web-based intervention; web-based intervention to reduce cannabis use; One way communication; No human contact; Length NR; Frequency NR | No treatment/waitlist control | Proportion of Cannabis Use in the Past Month (never, several times but not every week, several times in a week, every day) |
| (Croom et al., 2009); USA; N=3216 | College students; Avg age NR; 50.9% female; 25% Asian, 4% Black, 6% Latinx, 63% White, 2% other | Web-based intervention; web based interactive alcohol education course; One way communication; No human contact; Length NR; Frequency NR | No treatment/waitlist control | Mean # of drinks in post 2 weeks; Drinking Frequencies (mean number of drinks in previous 2 weeks) |
| (Croom et al., 2015) University A; USA; N=2007 | College students; Avg age NR; 49.9% female; 23% Asian, 4% Black, 7% Latinx, 60% White, 7% other | Web-based; Alcohol-Wise, an online alcohol education course; One way communication; No human contact; Length NR; Frequency NR | No treatment/waitlist control | Alcohol consumption; Typical week; Drank most; BAC; Drinking games; Heavy Episode; Extreme ritualistic alcohol consumption (male 11+ drinks or a female 8+ drinks on at least one day of the 2-week  drink calendar) |
| (Croom et al., 2015) University B; USA; N=2027 | College students; Avg age NR; 62.9% female; 23% Asian, 4% Black, 7% Latinx, 60% White, 7% Other | Web-based/computer intervention; Alcohol-Wise, an online alcohol education course; One way communication; No human contact; Length NR; Frequency NR | No treatment/waitlist control | Alcohol consumption; Typical week; Drank most; BAC; Drinking games; Heavy Episode; Extreme ritualistic alcohol consumption |
| (Crotwell, 2016); USA; N=168 | College students; Avg age 19.6; 73% female; 3% Asian, 2% Black, 39% Latinx, 45% White, 11% other | Web-based/computer intervention; A goal-setting learning module; One way communication; No human contact; Length NR; Frequency NR | No treatment/waitlist control | Average Drinks per Drinking Day -typical week and heaviest week; Max number of drinks in one day-heaviest week and typical week (DDQ); Total number of drinks- typical week and heaviest week (DDQ) |
| (Donohue et al., 2004); USA; N=113 | College students; Avg age 20.6; 55.8% female; 13% Asian, 8% Black, 12% Latinx, 63% White, 4% other | Web-based/computer intervention; CD-ROM psychoeducational prevention program for alcohol misuse; Two way communication; No human contact; Length NR; Frequency NR | Non-tech intervention: the Cognitive Behavioral Therapy Program involved  a systematic review of negative consequences associated with alcohol intoxication | Drinks per occasion (CAGE); Drinking days per month (CAGE); Drinks per month (CAGE) |
| (Donovan et al., 2012); USA; N=558 | College students; Avg age 18.4; 63.0% female; 1% Asian, 4% Black, 4% Latinx, 91% White, 1% other | Web-based/computer intervention; MyStudentBody-Parent: text and video based literature and lessons; Two way communication; Some human contact; Length 4 minutes; Frequency 1 | Treatment as usual: e-newsletter seven electronic newsletters sent to parent participants via e-mail. The content also focused on college-student AOD use and associated risks, but it was purely educational and did not strongly emphasize communication skill-building. | Binge drinking (how many times in the past 30 days they had consumed five drinks or more (four, for female participants) in about a two-hour period) |
| (Donovan et al., 2015); USA; N=319 | College students; Avg age 21.4; 73% female; 1% Asian, 7% Black, 14% Latinx, 77% White, 0% other | Web-based/computer intervention; wellness and success online intervention; One way communication; No human contact; Length 2 minutes; Frequency 0.25 | Non tech intervention: 3 educational newsletters with information that matched the content of the web-based intervention, plus monthly booster emails | Heavy episodic drinking (how many days in last 2 weeks they had consumed at least 4 (women) or 5 (men) standard drinks in a row); Past 30-day substance use [tobacco, cannabis, other illegal drugs] (Short Index of Problems- Drugs [SIP-D]); Days used in past 30 days (SIP-D); Drug related consequences [DUI, personal, physical, social, or financial] (SIP-D); Sum score (SIP-D); Max. drinks (past week); Total drinks (past week); Alcohol related consequences (RAPI) |
| (D. M. Doumas & Hannah, 2008); USA; N=124 | General young adult population; Avg age NR; 73% female; 0% Asian, 0% Black, 12% Latinx, 87% White, 1% other | Web-based/computer interventions; TBI a) web-based personalized feedback designed to reduce high-risk drinking, TBI b) web-based feedback plus a 15-minute motivational interviewing session; Two way communication; Some human contact; Length NR; Frequency NR | No treatment/waitlist control | Frequency of drinking to intoxication; Number of drinks in a typical week for each day of the week; Peak drinking quantity in the past month;  Typical weekend drinking (DDQ) |
| (M. Doumas et al., 2009); USA; N=87 | College students; Avg age 22.0; 41% female; 0% Asian, 0% Black, 13% Latinx, 79% White, 8% other | Web-based/computer intervention; Brief web-based program is designed to reduce high risk drinking; One way communication; No human contact; Length NR; Frequency NR | No treatment/waitlist control | Frequency of drinking to intoxication; Alcohol-related problems (RAPI); Weekly drinking quantity (DDQ modified version) |
| (D. M. Doumas et al., 2011); USA; N=350 | College students; Avg age 18.0; 65% female; 3% Asian, 0% Black, 4% Latinx, 90% White, 3% Other | Web-based/computer intervention; Brief web-based program is designed to reduce high risk drinking; One way communication; No human contact; Length NR; Frequency NR | No treatment/waitlist control | Frequency of drinking to intoxication; Peak drinking quantity in the past month; Alcohol-related consequences (RAPI); Weekly drinking quantity (modified DDQ) |
| (D. M. Doumas et al., 2014); USA; N=350 | College students; Avg age 18.0; 65% female; 3% Asian; 0% Black; 4% Latinx, 90% White, 3% other | Web-based/computer intervention; brief web-based program is designed to reduce high-risk drinking; One way communication; No human contact; Length NR; Frequency NR | No treatment/waitlist control | Drinking consequences: % within group (tracking university sanctions for violation of the campus alcohol policy); % within sanction (tracking university sanctions for violation of the campus alcohol policy); # of students who received sanctions |
| (Dunn et al., 2020); USA; N=121 | College students; Avg age 19.4; 33% female; 3% Asian, 4% Black, 11% Latinx, 75% White, 7% Other; | Web-based/computer intervention; 45 minute web-based interactive program; One way communication; Some human contact; Length NR; Frequency NR | Non-tech intervention: 3 brief motivational interviewing session lasting between 45 and 55 minutes | Mean BAC; Peak BAC; Mean drinks per sitting (DPS); Binge; Peak DPS; Drinking days per month; Mean drinks per week; Harms total |
| (Edwards et al., 2020); USA; N=161 | College students; Avg age 20.0; % female NR; 0% Asian, 6% Black, 5% Latinx, 84% White, 5% other | Text messaging intervention; text-messaging focused on protective behavioral strategies; One way communication; No human contact; Length NR; Frequency NR | No treatment/waitlist control | Alcohol related consequences (BYAACQ); alcohol consumption (DDQ) |
| (Elliott & Carey, 2012); USA; N=245 | College students; Avg age 20.5; 73% female; 57% White, 43% other | Web-based/computer intervention; Marijuana eCHECKUP TO GO (e-TOKE) for Universities & Colleges; One way communication; No human contact; Length NR; Frequency NR | No treatment/waitlist control | cannabis use/initiation (percent initiating in the past month) (survey question) |
| (Elliott, 2012); USA; N=317 | College students; Avg age 19.3; 52% female; 78% White, 22% other | Web-based/computer intervention; E-TOKE: web-based intervention for cannabis use; One way communication; No human contact; Length NR; Frequency NR | No treatment/waitlist control | Cannabis abuse symptoms; Cannabis dependence symptoms; Cannabis problems (Rutgers Marijuana Problems Inventory [RMPI]); days used in past month (slightly modified cannabis use items from the Revised Drug History Questionnaire) |
| (Epton et al., 2014); UK; N=1445 | College students; Avg age 18.9; 58.4% female; 9% Asian, 2% Black, 0% Latinx, 67% White, 22% other | App+ web intervention; U@Uni web-based resources; One way communication; No human contact; Length NR; Frequency NR | No treatment/waitlist control | Have not taken recreational drugs (single count method); Have taken recreational drugs (single count method); Mean dependency (AUDIT); Mean problems (AUDIT); Mean consumption (AUDIT); Mean number of days binge drinking in last 7 days (drinkers only) (General Lifestyle Survey); Mean units in last 7 days (General Lifestyle Survey); Mean alcohol objective (fatty acid ethyl esters, FAEE) |
| (Fearnow-Kenney et al., 2016); USA; N=647 | College students; Avg age 18.4; 53.2% female; 1% Asian, 10% Black, 5% Latinx, 83% White, 1% other | Web-based/computer intervention; myPlaybook: web-based intervention program designed specifically for college student-athletes that focuses on the prevention of alcohol and other drug use; One way communication; No human contact; Length NR; Frequency NR | No treatment/waitlist (delayed control) | Negative alcohol expectancies; general alcohol effects (Alcohol Expectancy Questionnaire) |
| (Gajecki et al., 2014); Sweden; N=1932 | College students; Avg age NR; % female NR; Race/ethnicity NR | Mobile application intervention; Promillekoll app; One way communication; No human contact; Length 7 minutes; Frequency NR | No treatment/waitlist control | Estimated blood alcohol content (eBAC)/week (DDQ); Peak eBAC/month (DDQ); Binge occasions (number per week) (AUDIT); Frequency (drinking occasions per week) (DDQ); Quantity (standard glasses/week) (DDQ) |
| (Gajecki et al., 2017); Sweden; N=186 | College students; Avg age 25.4; 69.1% female; Race/ethnicity NR | Mobile application intervention; web-based app focused on alcohol consumption; One way communication; No human contact; Length NR; Frequency NR | No treatment/waitlist control | Quantity, frequency, and binge occasions (# of standard glasses consumed or binge occasions during a 7 day period) (DDQ); Average eBAC over a 7 day period); Peak eBAC during last 30 days; EBAC (/week); Risk for excessive weekly drinking; Binge occasions (number per week); Peak eBAC (per month); Frequency (drinking occasions per week); Quantity (standard glasses per week) |
| (Ganz et al., 2018); Germany; N=981 | College students; Avg age 23.3; 51.6% female; Race/ethnicity NR | Web-based/computer intervention; eCHUG (social norms and protective behavioral strategies); One way communication; Human contact NR; Length NR; Frequency NR | No treatment/waitlist control | Peak BAC (Widmark factor); Frequency of heavy drinking occasions (Self-report questionnaire); Alcohol-related problems; Alcohol consumption (adaptation of the Daily Drinking Questionnaire) |
| (Geisner et al., 2015); USA; N=311 | College students; Avg age 20.1; 62.4% female; 19% Asian, 1% Black, 8% Latinx, 60% White, 12% other | Web-based/computer intervention; personalized feedback and in-depth psychoeducation regarding potential relationship between alcohol and depression; One way communication; No human contact; Length NR; Frequency NR | Non-tech intervention: referral to information, view treatment resource information for depression and substance use, but did not view any personalized feedback or intervention materials | Alcohol related consequences; Typical weekly drinks |
| (Gilmore et al., 2015); USA; N=207 | College students; Avg age 18.8; 100% female; 21% Asian, 4% Black, 10% Latinx, 58% White, 9% other | Web-based/computer intervention; web-based personalized feedback intervention using gender specific feedback; One way communication; No human contact; Length NR; Frequency NR | No treatment/waitlist control | Frequency of heavy episode drinking |
| (Gonzales et al., 2016); USA; N=80 | General young adult population; Avg age 20.5; 28.5% female; 7% Asian, 10% Black, 37% Latinx, 43% White, 1% other | Text messaging intervention; daily text messages guided by a disease management conceptual wellness mode for recovery from substance use disorders; Two way communication; No human contact; Length 12 minutes; Frequency 7 | Non-tech intervention: aftercare-as-usual control group consisted of the  standard protocol that was given at community-based  treatment programs | Used primary substance of abuse for which treatment was received; relapse to primary drug over time |
| (Goodness, 2019) USA; N=49 | College students; Avg age 26.2; 51% female; 8.15% Asian, 2.05% Black, 14.25% Latinx, 75.55% White | Web-based/computer intervention; E-CHECKUP TO GO Marijuana; Two way communication; No human contact, Length NR, Frequency NR | Assessment only | Percent of students have not used marijuana in the past 3 months, percent of students use marijuana at least once a month, percent of students use marijuana more than you, marijuana related consequences, frequency of use |
| (Haug et al., 2022)  ; Switzerland; N=1351 | College students; Avg age 17.3; 43.4% female; Race/ethnicity NR | "Ready4life," a mobile app-based program aimed at addiction prevention among apprentices, included personalized coaching through a conversational chatbot, focusing on promoting life skills and reducing risky behaviors; Two-way communication; No human contact; Length 16 weeks; Frequency 1 session/week | Assessment only (no intervention) | quantity of alcohol use past 30 days, quantity of cigarettes smoked past 30 days, cannabis smoking days past 30 days |
| (Hester et al., 2012) Experiment 1; USA; N=144 | College students; Avg age 20.7; 37.5% female; 1% Asian, 6% Black, 28% Latinx, 57% White, 10% other | Web-based/computer intervention; The College Drinker’s Check-up (CDCU); One way communication; No human contact; Length NR; Frequency NR | No treatment/waitlist control: assessment only | Number of drinks in two heavier episodes in previous month; standard drinks in a typical week; peak BAC in a typical; two heavier episodes in previous month; number of drinks in two heavier episodes in previous month |
| (Hester et al., 2012) Experiment 2; USA; N=82 | College students; Avg age 20.7; 37.5% female; 1% Asian, 6% Black, 28% Latinx, 57% White, 10% other | Web-based/computer intervention; The College Drinker’s Check-up (CDCU); One way communication; No human contact; Length NR; Frequency NR | No treatment/waitlist control: assessment only | Number of drinks in two heavier episodes in previous month; standard drinks in a typical week; peak BAC in a typical; two heavier episodes in previous month |
| (Hides et al., 2018); Australia; N=197 | General young adult population; Avg age 20.4; 77.7% female; Race/ethnicity NR | Mobile application intervention; protective behavioral strategies using Ray's Night Out interactive trivia questions; Two way communication; No human contact; Length NR; Frequency NR | No treatment/waitlist control | RSOD (risky single occasion drinking > 4 SUDs); typical number of SDUs (standard drink units); maximum number of SDUs; Frequency and number of problems (RAPI); AUDIT |
| (Hogan, 2005)  ; UK; N=75 | College students; Avg age 21.2; 60% female; Race/ethnicity Nr | Web-based/computer intervention; CBI: designed to motivate participants to change their current alcohol use; Two way communication; Some human contact; Length 12 minutes; Frequency Nr | No treatment/waitlist control | # of alcohol binges (Quantity/Frequency Alcohol Consumption Questionnaire); weekly alcohol consumption; drinking related problems (Drinker's Inventory of Consequences [DrInC-2R]) |
| (Hogan & Cox, 2024)  , UK; N=90 | College students; Avg age 21.05; 61.5% female; Race/ethnicity NR | Two brief, computerized motivational approaches designed to help university students reduce excessive drinking: Computerized Brief Intervention (CBI) and Enhanced Computerized Brief Intervention (CBI-E); One-way communication; No human contact; Length 12 weeks; Single session | No treatment | weekly units of alcohol consumed; number of binge episodes in the past 12 weeks; DrinC score (Drinker's Inventory of Consequences); for both CBI and CBI-E, males and females |
| (Hustad et al., 2010); USA; N=82 | College students; Avg age 18.1; 51% female; 89% White, 11% other | Web-based/computer intervention; e-CHUG: an online, self-administered, personalized normative feedback intervention that reports on various aspects of alcohol consumption; One way communication; No human contact; Length NR; Frequency NR | No treatment/waitlist control | Typical estimated BAC, past month; Peak estimated BAC, past month; typical drinks/drinking occasion, past month; Peak drinks/drinking occasion, past month; Number of negative alcohol consequences, past month (Young Adult Alcohol Consequences Questionnaire, or YAACQ); Ave. no. of drinks/week, past month; Number of heavy drinking episodes, past month |
| (Ji et al., 2023)  ; China; N=60 | General young adult population; Avg age 23.29; 100% female; 100% Asian, 0% other | Virtual reality (VR)-based motivational reinforcement and desensitization program aimed at reducing psychological cravings and addiction memories in young female adults with methamphetamine (MA) dependence; Two-way communication; Some human contact; Length 4 weeks; Frequency 2 sessions/week | Regular detoxification management | Psychological craving, addiction memory intensity |
| (Kazemi et al., 2020); USA; N=379 | College students; Avg age 19.0; 40.4% female; 3% Asian, 11% Black, 4% Latinx, 77% White, 5% other | Mobile application intervention; two brief in person brief motivational interventions sessions + app: includes personalized feedback and blood alcohol concentration tracking; Two way communication; Some human contact; Length 2 minutes; Frequency NR | Non-tech intervention: in-person Brief Motivational Interviewing | Peak BAC (Widmark’s basic formula); Total alcohol consequences in the past month (YACCQ); AUDIT score |
| (Koelen et al., 2024)  ; Netherlands, N=801 | College students; Avg age 23.9; 71.5 female; Race/ethnicity NR | A transdiagnostic internet-based Cognitive Behavioral Therapy (iCBT) program targeting university students with elevated anxiety and depression, included alcohol use and cannabis use outcomes; Two-way communication; Some human contact; Length NR; Frequency NR | Treatment as usual | Alcohol use, Substance use |
| (Kofler, 2021); Canada; N=63 | General young adult population; Avg age 22.1; 36.5% female; 33% Asian, 10% Black, 10% Latinx, 47% White, 0 % other | App and web intervention; brief personalized feedback intervention; Two way communication; No human contact; Length 2 minutes; Frequency 7 | No treatment/waitlist control | Cannabis-related consequences (51-item Marijuana Consequences Questionnaire, or MACQ) |
| (Kypri & McAnally, 2005); New Zealand; N=218 | College students; Avg age 20.2; 49% female; Race/ethnicity NR | Web-based/computer intervention; online assessment plus personalized feedback and social norms; One way communication; No human contact; Length NR; Frequency NR | No treatment/waitlist control | Percentage compliant with recommendations for alcohol consumed per occasion |
| (Laliberte, 2017)  USA; N=100 | College students; Avg age NR; 45% female; 41% Asian, 5% Black, 0% Latinx, 47% White, 7% other | Web-based/computer intervention; 15-20 minute interactive computer program based on principles of motivational interviewing; Two way communication; No human contact; Length NR; Frequency NR | Non-tech intervention: education only: participants were given nonpersonalized, factual information about alcohol consumption | YAACQ T2 score; Past month alcohol use (TLFB) |
| (Lane et al., 2012); USA; N=103 | College students; Avg age 18; 50.5% female; 0% Asian, 9% Black, 4% Latinx, 85% White, 2% other | Web-based/computer intervention; e-CHUG: an online, self-administered, personalized normative feedback intervention that reports on various aspects of alcohol consumption; One way communication; No human contact; Length NR; Frequency NR | Non-tech intervention: Self management alcohol condition | # of drinks consumed |
| (Leavens et al., 2020); USA; N=268 | College students; Avg age 19.8; 61% female; 1% Asian, 3% Black, 5% Latinx, 83% White, 8% other | Web-based/computer intervention; personalized feedback on protective behavioral strategies; One way communication; No human contact; Length NR; Frequency NR | No treatment/waitlist control | Past-month experience with alcohol-related consequences (BYAACQ); Peak drinking quantity (DDQ); Drinks per week (DDQ) |
| (Lee et al., 2014); USA; N= 783 | College students; Avg age 20.5, 56.1% female; 19.9% Asian, 12.5% other, 67.6% White | SB BASICS (Spring Break Brief Alcohol Screening and Intervention for College Students), using a web-based model of delivery | Assessment only control | Spring Break related alcohol use, alcohol related consequences |
| (Lee et al., 2021); USA; N=142 | College students; Avg age 22.8; 69.7% female; 13% Asian, 6% Black, 14% Latinx, 59% White, 8% other | Web-based/computer intervention; intervention feedback session consisted of a 1-hr conversation guided by personalized feedback and delivered using Motivational Interviewing-style; Two way communication; No human contact; Length NR; Frequency NR | No treatment/waitlist control | Estimated peak BAC (DDQ); Number of binge episodes (DDQ); Drinks per week total (DDQ); Estimated peak BAC (DDQ); Alcohol-related consequences (BYAACQ) |
| (Leeman et al., 2016); USA; N=208 | College students; Avg age 19.9; 62.5% female; 0% Asian, 2% Black, 4% Latinx, 60% White, 35% other | Web-based/computer based intervention; THRIVE: protective behavioral strategies and personalized feedback on alcohol consumption patterns; One way communication; No human contact; Length NR; Frequency NR | No treatment/waitlist control (assessment + education) | Frequency of drinking days per week (abbreviated DDQ-R); heavy episodic drinking frequency, past 30 days (abbreviated DDQ-R); Overall drinks per week (abbreviated DDQ-R); alcohol-related problems (RAPI); peak number of drinks in a day, past 30 days (participant self-report) |
| (Lewis et al., 2014); USA; N=480 | College students; Avg age 20.4; 57.6% female; 13% Asian, 0% Black, 0% Latinx, 70% White, 18% other | Web-based/computer intervention; VSee, a web-conferencing and screen-sharing software package, includes personalized feedback and 1-hr conversation using motivational interviewing; One way communication; No human contact; Length NR; Frequency NR | No treatment/waitlist control | Perceived drinking frequency (measures for perceived descriptive drinking); perceived drinks per week; perceived drinks per occasion; perceived drinks consumed prior to sex; perceived frequency of drinking prior to sex; Typical drinking frequency and typical drinks per occasion (using items from the  Quantity/Frequency/Peak Alcohol Use Index); Alcohol-related negative consequences (BYAACQ); drinks per week (DDQ) |
| (Lovecchio, 2008)  ; USA; N=1093 | College students; Avg age NR; 53.7% female; 7% Asian, 5% Black, 2% Latinx, 81% White, 6% other | Web-based/computer intervention; on-line alcohol prevention program intended to deliver information to entire groups of students; One way communication; No human contact; Length 2 minutes; Frequency unlimited log on/off | No treatment/waitlist control: assessment only/delayed control | Number of days during a typical week students drink (item 151 on attitudes/behavior survey); Negative Mental Consequences of Alcohol Use (attitudes/behavior survey); Responsible Drinking at Parties/Events (attitudes/behavior survey); avg number of drinks consumed on weekend (item 121 and 122 on attitudes/behavior survey)l average number of drinks consumed during a single event (item 149 on attitudes/behavior survey) |
| (Lovecchio et al., 2010); USA; N=1620 | College students; Avg age 18; 54.3% female; 81% White, 20% other | Web-based/computer intervention; AlcoholEdu for College 8.0; One way communication; No human contact; Length NR; Frequency NR | No treatment/waitlist control | Proportion of heavy, episodic drinkers; High-risk alcohol behaviors; Drinking-related behavioral consequences; Drinking-related psychological consequences; Total number of drinks past 2 weeks |
| (M. Mason et al., 2014); USA; N=18 | College students; Avg age 19.3; 55.5% female; 0% Asian, 22% Black, 11% Latinx, 67% White, 0% other | Personalized text messaging intervention; Two way communication; No human contact; Length .5 minute, Frequency 4-6 messages/day | No treatment/waitlist control | Avg #drinks/occasion last month (Participants answered questions concerning the frequency with which they used alcohol, cannabis, and other substances in the past month); #Drinks, last occasion (same as above); Maximum #drinks past month (same as above); #Drinks past week (same as above); AUDIT total (10 item AUDIT) |
| (M. J. Mason et al., 2018); USA; N=101 | General young adult population; Avg age 20.3; 42.7% female; 4% Asian, 9% Black, 2% Latinx, 77% White, 7% other | Text messaging intervention; Peer Network  Counseling-txt (PNC-txt) using motivational interviewing principles; Two way communication; No human contact; Length 4 minutes; Frequency 3.5 | No treatment/waitlist control | Past 12-month felt such strong urge to use that you used; Past 12-month problems with relationships due to cannabis use (% yes); Past 30- day kept using cannabis when promised self not to; Past 30-day memory problems; past 30 day number of days used largest amount; past 30-day cannabis use; peer network health; Past 30-day memory problems |
| (Maybery et al., 2022); Australia; N=41 | General young adult population; Avg age 21.8; 92.7% female; 10% White, 90% other | Web-based/computer intervention; mi.spot (mental illness, supportive, preventative, online, targeted) is a 6 week, professionally moderated and manualized online intervention; Two way communication; Some human contact; Length 6 minutes; Frequency NR | No treatment/waitlist control | Substance use (NR) |
| (McCambridge et al., 2013); Sweden; N=7809 | College students; Avg age NR; 52.2% female; Race/ethnicity NR | Web-based/computer intervention; routine provision of assessment and feedback via email; One way communication; No human contact; Length NR; Frequency NR | No treatment/waitlist control | Risky drinking (according to the Swedish definition of at least one heavy episode of drinking 5 drinks [of 12 g of alcohol] or more for men or 4 drinks or more for women in the past month or weekly consumption of more than 14 drinks for men or more than 9 drinks for women); frequency of drinking (AUDIT-C); number of heavy episodes of drinking per month (AUDIT); typical quantity consumed (AUDIT); AUDIT-C (The first three items of the Alcohol Use Disorders Identification Test) |
| (Miller, 2015)  USA; N=191 | College students; Avg age 19.8; 50.9% female; 90% White, 10% other | Email (+ computer/web) intervention; personalized feedback intervention with norms only as part of feedback; One way communication; No human contact; Length NR; Frequency NR | No treatment/waitlist control | Peak BAC (DDQ); alcohol related consequences (BYAACQ); drinking quantity per week (DDQ) |
| (Miller et al., 2016); USA; N=212 | College students; Avg age 19.9; 57% female; 1% Asian, 2% Black, 3% Latinx, 87% White, 7% other | Computer intervention; PFI-Enhanced: personalized feedback intervention -norms descriptive normative feedback plus feedback on spending money on alcohol; One way communication; No human contact; Length NR; Frequency NR | No treatment/waitlist control | Peak BAC (Widmark’s [1932] formula); alcohol-related consequences (BYAACQ); drinks consumed per week (DDQ) |
| (Moore et al., 2005); USA; N=116 | College students; Avg age 21.7; 57.8% female; 9% Asian, 8% Black, 10% Latinx, 70% White, 4% other | Web-based/computer intervention; website based "newsletters" focused on alcohol use; One way communication; No human contact; Length 4 minutes; Frequency 1 | Non-tech intervention: same newsletters as the web-based intervention sent through postal mail | 12-month frequency of alcohol use ("Alcohol & You" web-based survey for all below); 2-week frequency of binge drinking; 30-day quantity of alcohol use; 30-day frequency of "get drunk"; 30-day frequency of binge drinking; 30-day quantity of "get drunk"; 30-day frequency of alcohol use; 30-day greatest number of drinks at one time |
| (Moreira et al., 2012); UK; N=1751 | College students; Avg age NR; 61.5% female; Race/ethnicity NR | Web-based/computer intervention; PNF: brief personalized normative feedback via email; One way communication; No human contact; Length NR; Frequency NR | No treatment/waitlist control | Weekly drinking (proportion) (drinking diary); frequency of drinking per month (one item asking how often the respondent drank); Alcohol-related problems (a newly-developed self-reported scale with nine possible problems); quantity of drinking per occasion (one item asking how many drinks/units a respondent usually consumed on a drinking occasion); AUDIT score |
| (Murphy et al., 2015); USA; N=133 | College students; Avg age 18.6; 49.6% female; 1% Asian, 30% Black, 2% Latinx, 64% White, 3% other | Web-based/computer intervention; e-CHUG: an online, self-administered, personalized normative feedback intervention (PNFI) that reports on various aspects of alcohol consumption; One way communication; No human contact; Length NR; Frequency NR | 1 Non-tech intervention: BASICS, an individualized alcohol assessment and feedback intervention for college students that is designed for delivery in two 50-min sessions and includes cognitive-behavioral skills training, motivational enhancement and personalized feedback  2 No treatment/waitlist control | Alcohol problems (YAACQ); drinks per week (DDQ); self-ideal discrepancy (how alcohol was affecting their relationships with friends on a scale from 1 to 7); Normative and self-ideal discrepancy (using the Discrepancy Ratings Questionnaire); heavy drinking (how many times in the past month they had engaged in a heavy drinking episode in a 2-hour period of time.) |
| (Murphy et al., 2010) Study 1; USA; N=74 | College students; Avg age 21.2; 59% female; 2% Asian, 23% Black, 2% Latinx, 73% White, 0% other | Web-based/computer intervention; e-CHUG: an online, self-administered, personalized normative feedback intervention (PNFI) that reports on various aspects of alcohol consumption; One way communication; No human contact; Length NR; Frequency NR | Non-tech intervention: BASICS, an individualized alcohol assessment and feedback intervention for college students that is designed for delivery in two 50-min sessions and includes cognitive-behavioural skills training, motivational enhancement and personalized feedback | Self-ideal discrepancy (assessed how alcohol was affecting their relationships with friends on a scale from 1 to 7); Normative and self-ideal discrepancy (Discrepancy Ratings Questionnaire); heavy drinking (how many times in the past month they had engaged in a heavy drinking episode in a 2-hour period of time); drinks per week (DDQ) |
| (Neighbors et al., 2006); USA; N=217 | College students; Avg age 19.67; 55.6% female; 98% White, 2% other | Web-based/computer intervention; computer based; Communication type NR; No human contact; Length NR; Frequency NR | No treatment/waitlist control | Alcohol-related problems (modified RAPI); Alcohol consumption (DDQ) |
| (Neighbors et al., 2009); USA; N=295 | College students; Avg age NR; 58.3% female; 24% Asian, 2% Black, 0% Latinx, 59% White, 15% other | Web-based/computer intervention; Web-based personalized feedback 2 days before their 21st birthday; One way communication; No human contact; Length NR; Frequency NR | No treatment/waitlist control | BAC on 21st birthday (modified DDQ); No. drinks on 21st birthday (modified DDQ); Intended BAC (modified DDQ); Intended no. drinks on 21st birthday (modified DDQ) |
| (Neighbors et al., 2012); USA; N=599 | College students; Avg age 21; 53.9% female; 16% Asian, 0% Black, 0% Latinx, 68% White, 16% other | Web-based/computer intervention; 21 WEB BASICS intervention consisted of the components of 21 BASICS individual intervention, but was presented in a webcompatible format two days prior to the 21st birthday; One way communication; No human contact; Length NR; Frequency NR | No treatment/waitlist control | eBAC on 21st birthday |
| (Neville et al., 2013); Scotland; N=60 | College students; Avg age 21.5; % female NR; Race/ethnicity NR | App + web intervention; digital data from an anklet analyzed by AMS Technologies, tracked transdermal alcohol concentration (TAC); Two way communication; Some human contact; Length 2 minutes; Frequency 1 | No treatment/waitlist control | Total units of alcohol consumed (AUDIT and 14-day alcohol TLFB) |
| (Palfai et al., 2014); USA; N=1080 | College students; Avg age 19.7; 57.5% female; 2% Asian, 0% Black, 14% Latinx, 84% White, 0% other | Web-based/computer intervention; Marijuana eCHECKUP TO GO; One way communication; No human contact; Length NR; Frequency NR | Treatment as usual: feedback on general health-related behaviors | # of cannabis-related negative consequences (Cannabis-related Consequences—The Marijuana Problems Scale); # of days using cannabis in past 90 days (MJ_FRQ) |
| (Paschall & Bersamin, 2006)  ; USA; N=370 | College students; Avg age 18.1; 52% female; 42% Asian, 3% Black, 16% Latinx, 30% White, 9% other | Web-based/computer intervention; online course with 5 units, interactive animations, online assignments, and a quiz; One way communication; No human contact; Length 6 minutes; Frequency NR | No treatment/waitlist control | Frequency of getting drunk in past 30 days; frequency of alcohol use in past 30 days; frequency of having five or mor drinks in past 30 days; negative drinking consequences (27 item index) |
| (Patrick et al., 2023)  ; USA; N=891 | College students; Avg age 18.5; 62.4% female; 10.6% Asian, 4% Black, 4.8% Latinx, 75% White, 5.6% other | A two-stage Adaptive Preventive Intervention (API) aimed at reducing binge drinking and alcohol-related consequences among college students; Two-way communication; No human contact; Length 16 weeks; Frequency 2 sessions/week | Assessment only | Binge drinking, Alcohol-related consequences |
| (Pedersen et al., 2017); USA; N=343 | College students; Avg age 21.1; 78% female; 14 % Asian, 2% Black, 2% Latinx, 72% White, 10% other | Web-based/computer interventions; TBI a) brief online personalized normative feedback intervention (PNF) to prevent increased and problematic use by correcting misperceptions of study abroad student drinking norms, correcting misperceptions of country-specific native adult drinking norms, and promoting positive adjustment and engagement into the host culture (sojourner adjustment) prior to departure abroad; TBI b) PNF + a sojourner adjustment feedback intervention; One way communication; No human contact; Length NR; Frequency NR | No treatment/waitlist control | Alcohol-related consequences (RAPI); drinks per week (DDQ) |
| (Pietsch et al., 2023); Germany; N=4591 | College students; Avg age 19.2; 45.2% female; Race/ethnicity NR | “Meine Zeit ohne—Die Challenge” (“My time off—the Challenge”), an app-based program targeting vocational students in Germany, aimed to reduce substance use, gambling, and digital media usage; Two-way communication; Some human contact; Length 2 weeks; Frequency 2 sessions/week | No treatment/waitlist control | Alcohol use, cigarette smoking, use of e-products, cannabis use |
| (Pischke et al., 2021); Germany; N=1255 | College students; Avg age 23.6; 59% female; Race/ethnicity NR | Web-based/computer intervention; gender-specific, normative feedback administered via website; One way communication; No human contact; Length NR; Frequency NR | No treatment/waitlist control (delayed) | Unchanged cannabis consumption; unchanged tobacco consumption; unchanged episodes of drunkenness; unchanged alcohol consumption; increased alcohol consumption; decreased episodes of drunkenness; decreased alcohol consumption; increased episodes of drunkenness; increased tobacco consumption; increased cannabis consumption; decreased tobacco consumption; decreased cannabis consumption |
| (Reavh Tley et al., 2014); New Zealand; N=767 | College students; Avg age 24.4; 60.9% female; Race/ethnicity NR | App + web intervention; Mindwise: an electronic educational course; One way communication; Some human contact; Length 64 minutes; Frequency NR | No treatment/waitlist control | % risky or hazardous levels of drinking (those who had drunk alcohol at some point in their lives were administered the AUDIT) |
| (Rew et al., 2022); USA; N= 602 | General young adult population; Avg age 21; 45% female; 50% White, 50% other | Laptop based one-on-one intervention focused on communication, goal-setting, and substance use; One-way communication; Some human contact; Length NR; Frequency NR | No treatment/waitlist control | Substance refusal self-efficacy |
| (Ridout & Campbell, 2014); Australia; N=244 | College students; Avg age 19; 78% female; 19% Asian, 0% Black, 0% Latinx, 56% White, 25% other | Social media-based intervention; social norms feedback in the form of Facebook private messages generated using Microsoft Word macros; One way communication; Some human contact; Length NR; Frequency NR | No treatment/waitlist control | # of drinking days in past month (AUDIT); # of drinks in past month (AUDIT) |
| (Riggs et al., 2018); USA; N=298 | College students; Avg age 20; 49% female; 2% Asian, 4% Black, 0% Latinx, 86% White, 8% other | Web-based/computer intervention; Marijuana eCHECKUP TO GO, a web-based marijuana use intervention providing university-specific personalized feedback  with normative information and protective behavioral strategies to students; One way communication; No human contact; Length NR; Frequency NR | Treatment as usual: healthy stress management | Weeks using cannabis/typical month (ranges of times used cannabis per week); days high/week; hours high/using day; # of time periods high per week; hours high/week; Cannabis use consequences (by summing the total number of consequences experienced in the last month and the average severity of the endorsed consequences, measured on a 5-point scale from “never” to “always” experienced.) |
| (Riordan et al., 2023); New Zealand; N=783 | College students; Avg age 18.1; 77.7% New Zealand European, 4.7 % Maori, 7.2 % Asian, 10.4 %  other) | Text messaging intervention; Two way communication; No human contact; Length 13 minutes; Frequency NR | No treatment/waitlist control (assessment only) | Weekend drinks (assessed using text message questions); Overall O'Week drinking measure; Consequences (B-YAACQ); typical weekly alcohol use (modified DDQ) |
| (Scharer, 2019); USA; N=310 | College students; Avg age 22.7; 48% female; 0% Asian, 48% Black, 0% Latinx, 70% White, 0% other | Web-based/computer intervention; e-CHUG: an online, self-administered, personalized normative feedback intervention (PNFI) that reports on various aspects of alcohol consumption; One way communication; No human contact; Length NR; Frequency NR | No treatment/waitlist control | Peak quantity of alcohol (Quantity/Frequency Index (QFI)); alcohol problems (RAPI); drinks per week (DDQ) |
| (Schuckit et al., 2015); USA; N=454 | College students; Avg age 18.2; 63.1% female; 29% Asian, 0% Black, 10% Latinx, 0% White, 60% other | Web-based/computer interventions; videos with didactic lectures incorporating MI and BI techniques; TBI a) Low level of Response (Low  LR) where all examples were given the context of the Low LR model of heavy drinking; TBI b) a State Of The Art (SOTA) intervention where the same lessons were taught but  without an emphasis on LR; One way communication; No human contact; Length 4 minutes, Frequency 1 | No treatment/waitlist control | 4+ times/occasion (Semi-Structured Assessment for the Genetics of Alcoholism [SSAGA]); usual quantity (SSAGA); usual frequency (SSAGA); maximum quantity (SSAGA) |
| (Shuai et al., 2022); UK; N=52 | College students; Avg age 20.4; 87% female; Race/ethnicity NR | App + web intervention; PowerPoint presentations containing text and images; One way communication; No human contact; Length 6 minutes; Frequency NR | Treatment as usual: control intervention video | Daily drinking |
| (Shuai et al., 2024)  ; South Africa; N=50 | General adult population; Avg age 19.2; 42% female; Race/ethnicity NR | An online Functional Imagery Training (FIT) focused on reducing alcohol consumption among hazardous student drinkers who consumed alcohol to cope with negative emotions; Two-way communication; Some human contact; Length 4 weeks; Frequency 1 session/week | Standard risk information | Drink motives-social, Drink motives-coping, Drink motives-enhancement, Drink motives-conformity |
| (Stapinski et al., 2021); Australia; N=123 | General young adult population; Avg age 21.6; 67.5% female; Race/ethnicity NR | Web-based/computer intervention; Youth focused, web-based cognitive behavioral therapy (CBT) program with weekly psychologist support via email/phone; Two way communication; Some human contact; Length 8 minutes; Frequency NR | Non-tech intervention: participants in the control group received an online  information pamphlet outlining the effects of alcohol and risks of  overuse, the Australian National Health and Medical Research Council’s recommended guidelines for safe alcohol consumption, and a list  of links to national telephone helplines and alcohol information websites. | Alcohol use above recommended guidelines (defined according to Australian National Health and Medical Research council's guidelines of < 10 standard drinks per week); Alcohol use above recommended guidelines (same as above); Probable alcohol use dependence (recommended cut-off of >20 on the AUDIT); Average drinks per day, past month (TLFB); Binge drinking frequency, past month (TLFB); Alcohol-related consequences (b-YAACQ); Hazardous alcohol use (AUDIT total) |
| (Stappenbeck et al., 2021); USA; N=200 | College students; Avg age 20.9; 100% female; 14% Asian, 1% Black, 10% Latinx, 69% White, 7% other | Web-based/computer intervention; brief 5–10 min skill modules, presented once per day in a web-based format that was mobile compatible; One way communication; No human contact; Length 2 minutes, Frequency 7 | No treatment/waitlist control: assessment only | Heavy episodic drinking (One item taken from the National Institute on Alcohol Abuse and Alcoholism); Drinks per week (DDQ) |
| (Strohman et al., 2016); USA; N=58 | College students; Avg age NR; 79.4% female; 12% Asian, 3% Black, 14% Latinx, 54% White, 16% other | Web-based/computer intervention; Alcohol-Wise: an alcohol education course designed for prevention and as an intervention; One way communication; No human contact; Length NR; Frequency NR | No treatment/waitlist control | Peak BAC (eCHECKUPTOGO assessment); typical drinking days (eCHECKUPTOGO assessment); heaviest drinking occasion (eCHECKUPTOGO assessment); total negative consequences (AUDIT) |
| (Suffoletto et al., 2012); USA; N=45 | General young adult population; Avg age 21; 64% female; 0% Asian, 25% Black, 0% Latinx, 0% White, 76% other | Text messaging intervention; text message based intervention with alcohol assessments; Two way communication; No human contact; Length 12 minutes; Frequency 1 | No treatment/waitlist control | Drinks per drinking day (maximum number of drinks per drinking day in prior month); Heavy drinking days (how many heavy drinking days in the prior month) |
| (Suffoletto et al., 2015); USA; N=765 | General young adult population; Avg age 21.9; 65.4% female; 0% Asian, 45% Black, 7% Latinx, 49% White, 0% other | Text messaging intervention; SMS based intervention with texts focused on drinking behaviors; Two way communication; No human contact; Length 12 minutes; Frequency 1 | Treatment as usual | Binge drinking prevalence (TLFB); alcohol-related injury prevalence (Injury Behavior Checklist); drinks per day drinking day (TLFB); days with binge drinking (TLFB) |
| (Tanner et al., 2021); USA; N=3098 | College students; Avg age 18; 64% female; 0% Asian, 19% Black, 58% Latinx, 18% White, 5% other | Web-based/computer intervention : itMatters; One way communication; No human contact; Length NR; Frequency NR | Non-tech intervention: active comparison focused on mental health and sleep | Hookup and alcohol risk (intersection of hookup and alcohol); sex and alcohol risk (intersection of sex and alcohol) |
| (Teeters et al., 2022a); USA; N=97 | College students; Avg age 21.3; 67.4% female; 2% Asian, 7% Black, 1% Latinx, 80% White, 10% other | App + web intervention; personalized feedback intervention + Motivational Interviewing-style interactive text messaging intervention: link sent via text message to a secure website containing personalized feedback; One way communication; No human contact; Length NR; Frequency NR | Treatment as usual: Substance information control condition (IC) | Driving after combined use of alcohol and cannabis; past month cannabis use days (modified TLFB); driving after cannabis use |
| (Teeters et al., 2022b)  ; USA; N=97 | College students; Avg age 21.34; 67.4% female; 2% Asian, 7% Black, 1% Latinx, 80.4% White, 9.6% other | A mobile phone-based brief intervention targeting college cannabis users to reduce driving after cannabis use and riding with a cannabis-impaired driver, using Personalized Feedback (PF) and Motivational Interviewing (MI); Two-way communication; Some human contact; Length 3 weeks; Frequency 1 session/week | Substance use information | Driving after cannabis use, Riding with a cannabis-impaired driver, Driving after combined use of alcohol and cannabis, Past month cannabis use days |
| (Thomas et al., 2018); Sweden; N=896 | College students; Avg age 25.5; 57% female; Race/ethnicity NR | Text messaging intervention; 6-week automated text message–based program; Two way communication; No human contact; Length 6 minutes; Frequency 7 | Treatment as usual: the typical practice at the SHCs, besides motivating advice delivered face-to-face, is to recommend a website to the students where they can estimate their alcohol consumption, receive  feedback on their drinking levels | Frequency of binge drinking; Highest eBAC; Number of negative consequences of excessive drinking; Weekly alcohol consumption |
| (K. Thompson et al., 2018); Canada; N=245 | College students; Avg age 17.8; 66% female; Race/ethnicity NR | Web-based/computer intervention; e-CHUG: an online, self-administered, personalized normative feedback intervention (PNFI) that reports on various aspects of alcohol consumption; One way communication; No human contact; Length NR; Frequency NR | No treatment/waitlist control | Drinking frequency (e-CHUG program assessment); Drinking Harm (AUDIT); Drinking quantity (e-CHUG program assessment) |
| (R. G. Thompson et al., 2020); USA; N=60 | General young adult population; Avg age 19.1; 30% female; 0% Asian, 58% Black, 40% Latinx, 3% White, 0% other | Mobile application intervention; OnTrack BMI: brief daily technology-supported self-monitoring of alcohol, cannabis, and sexual risk behaviors; Two way communication; Some human contact; Length 4 minutes; Frequency NR | Treatment as usual: included two components: (a) substance use treatment and referral and HIV testing, as regularly offered to all participants who report substance use and sexual risk behaviors at the shelter, and (b) brief meetings (20 min or less) with a research coordinator | Number of drinks (TLFB); times used cannabis (TLFB) |
| (Tossmann et al., 2011); Germany; N=1292 | General young adult population; Avg age 24.7; 29.5% female; Race/ethnicity NR | Web-based/computer intervention; web based counseling program; Two way communication; Some human contact; Length 7.1 minutes; Frequency NR | No treatment/waitlist control | Satisfaction with life (Satisfaction with Life Scale-5 Items); Depression (General Depression Scale); Use frequency days/last 30 days (a German adaptation of the Drug-Taking Confidence Questionnaire-8); Anxiety (State-Trait Anxiety Inventory); Use quantity grams/last 30 days (a German adaptation of the Drug-Taking Confidence Questionnaire-8) |
| (Towe & Stephens, 2014); USA; N=82 | College students; Avg age 19.6; 47.6% female; 2% Asian, 1% Black, 6% Latinx, 88% White, 3% other | Web-based/computer intervention; brief web-based feedback intervention; One way communication; No human contact; Length NR; Frequency NR | Treatment as usual: education only | DSM-IV total criteria for cannabis abuse (DSM-IV classification); days of other drug use in past 30 days; DSM-IV total criteria for cannabis dependence (DSM-IV classification); cannabis use consequence (Marijuana Problems Index); days of alcohol use in past 30 days; cannabis use consequence (Cannabis Use Problems Identification Test); days of cannabis use in past 30 days |
| (Tucker et al., 2021); USA; N=77 | General young adult population; Avg age 22.6; 18.4% female; 44.12% Black, 29.41% Latinx, 11.76% White, 14.71% other | Text messaging intervention; CRUSH IT: an automated text based intervention for smoking cessation; Two way communication; Some human contact; Length 6; Frequency 31.5 | Treatment as usual: brief group smoking cessation counseling session delivered by a trained Bachelor’s degree level facilitator | 7-day point prevalence smoking abstinence, 7-day point prevalance abstinence (% who quit smoking), post-quit smoking date abstinence (1^st^, 2^nd^, 3^rd^, and 4^th^ week), # of nicotine patches used, % any nicotine patch use |
| (Voogt et al., 2013); Netherlands; N=913 | College students; Avg age 20.8; 39.8% female; Race/ethnicity NR | Web-based/computer intervention; WDYD: a single session web-based brief alcohol intervention using MI protocol; One way communication; No human contact; Length NR; Frequency NR | No treatment/waitlist control | Heavy drinking; frequency of binge drinking (how often they had drunk five or more glasses of standard alcohol units in the previous week on one drinking occasion); weekly alcohol consumption |
| (Wagener et al., 2012); USA; N=152 | College students; Avg age 20.3; 45.4 % female; 86% White, 14% other | Web-based/computer intervention; DrAFT-CS (Drinking Assessment and Feedback Tool for College Students): a computer-delivered interactive program using personalized feedback and perceived norms; Two way communication; Some human contact; Length NR; Frequency NR | No treatment/waitlist control | Typical BAC (BAC for each day of the week using Widmark's formula, then by averaging the BACs across the week); Peak BAC (# and duration of their heaviest drinking occasion in the past month and then combining this info along with their gender and weight, again using Widmark's formula); alcohol-related problems (BYAACQ); weekly quantity (DDQ) |
| (Walters et al., 2007); USA; N=106 | College students; Avg age NR; 48.1% female; 73% White, 27% other | Web-based/computer intervention; web-based personalized feedback report; One way communication; No human contact; Length NR; Frequency NR | No treatment/waitlist control | Peak BAC; consequences related to drinking in the last 30 days (RAPI); Drinks per week (Participants were asked to think about a typical week during the last month and for each day, to record the number of standard drinks they typically consumed on that day) |
| (Walters et al., 2009); USA; N=279 | College students; Avg age 19.8; 64.2% female; 86% White, 15% other | Web-based/computer intervention; e-CHUG: an online, self-administered, personalized normative feedback intervention (PNFI) that reports on various aspects of alcohol consumption; Two way communication; Some human contact; Length NR; Frequency NR | No treatment/waitlist control | Peak BAC (DDQ); alcohol related problems (RAPI); mean drinks per week (DDQ) |
| (Weaver et al., 2014); USA; N=176 | College students; Avg age 19.7; 49.4% female; 83% White, 17% other | Web-based/computer interventions; TBI a) DrAFT-CS (Drinking Assessment and Feedback Tool for College Students): a computer-delivered interactive program using personalized feedback and perceived norms, and an educational slide presentation on alcohol related topics; One way communication; TBI b) DrAFT-CS plus moderation skills (DrAFT-CS+); No human contact; Length NR; Frequency NR | No treatment/waitlist control | Average BAC, peak BAC (TLFB); consequences associated with binge drinking (B-YAACQ); alcohol use difficulties (AUDIT); peak month (Frequency-Quantity Questionnaire); typical week total (Frequency-Quantity Questionnaire) |
| (Witkiewitz et al., 2014); USA; N=94 | College students; Avg age 20.5; 27.7% female; 21% Asian, 3% Black, 2% Latinx, 71% White, 2% other | Text messaging intervention; a mobile intervention based on BASICS feedback materials ; One way communication; No human contact; Length 2 minutes; Frequency NR | Two no treatment/waitlist controls: mobile assessments only, minimal assessments only | Frequency of alcohol-related problems over the past year (YAAPST); days of drinking and smoking per week (DDQ and Daily Smoking Questionnaire); Typical alcohol quantity (DDQ); Frequency of drinking (DDQ); Number of drinking days (DDQ); Total consumption (DDQ) |

Note. yo= years old. NR=not reported.

Alfonso, J., Hall, T. V., & Dunn, M. E. (2013). Feedback‐Based Alcohol Interventions for Mandated Students: An Effectiveness Study of Three Modalities. *Clinical Psychology & Psychotherapy*, *20*(5), 411–423. https://doi.org/10.1002/cpp.1786

Andersson, C. (2015). Comparison of WEB and Interactive Voice Response (IVR) Methods for Delivering Brief Alcohol Interventions to Hazardous-Drinking University Students: A Randomized Controlled Trial. *European Addiction Research*, *21*(5), 240–252. https://doi.org/10.1159/000381017

Andrade, S. B., Greve, J., & Lesner, R. V. (2024). Changing university students’ alcohol use with a web-based intervention: Evidence from a randomized controlled trial. *Journal of Public Health*. https://doi.org/10.1007/s10389-024-02302-2

Arazan, C., Costelloe, M. T., & Willingham, M. T. (2023). Evaluation of a brief harm reduction intervention to reduce celebratory drinking among college students. *Journal of American College Health*, *71*(4), 1293–1300. https://doi.org/10.1080/07448481.2021.1927048

Barnett, N. P., Murphy, J. G., Colby, S. M., & Monti, P. M. (2007). Efficacy of counselor vs. Computer-delivered intervention with mandated college students. *Addictive Behaviors*, *32*(11), 2529–2548. https://doi.org/10.1016/j.addbeh.2007.06.017

Bedendo, A., Gaume, J., McCambridge, J., Noto, A. R., & Souza-Formigoni, M. L. O. (2024). Booster effects and mechanisms of web-based personalised normative feedback alcohol intervention for college students: A pragmatic randomised controlled trial. *Drug and Alcohol Dependence*, *260*, 1–10. https://doi.org/10.1016/j.drugalcdep.2024.111337

Bendtsen, P., Bendtsen, M., Karlsson, N., White, I. R., & McCambridge, J. (2015). Online Alcohol Assessment and Feedback for Hazardous and Harmful Drinkers: Findings From the AMADEUS-2 Randomized Controlled Trial of Routine Practice in Swedish Universities. *Journal of Medical Internet Research*, *17*(7), e170. https://doi.org/10.2196/jmir.4020

Bendtsen, P., McCambridge, J., Bendtsen, M., Karlsson, N., & Nilsen, P. (2012). Effectiveness of a Proactive Mail-Based Alcohol Internet Intervention for University Students: Dismantling the Assessment and Feedback Components in a Randomized Controlled Trial. *Journal of Medical Internet Research*, *14*(5), e142. https://doi.org/10.2196/jmir.2062

Bernstein, M. H., Stein, L. A. R., Neighbors, C., Suffoletto, B., Carey, K. B., Ferszt, G., Caron, N., & Wood, M. D. (2018). A text message intervention to reduce 21st birthday alcohol consumption: Evaluation of a two-group randomized controlled trial. *Psychology of Addictive Behaviors*, *32*(2), 149–161. https://doi.org/10.1037/adb0000342

Bertholet, N., Cunningham, J. A., Faouzi, M., Gaume, J., Gmel, G., Burnand, B., & Daeppen, J. (2015a). Internet‐based brief intervention for young men with unhealthy alcohol use: A randomized controlled trial in a general population sample. *Addiction*, *110*(11), 1735–1743. https://doi.org/10.1111/add.13051

Bertholet, N., Cunningham, J. A., Faouzi, M., Gaume, J., Gmel, G., Burnand, B., & Daeppen, J.-B. (2015b). Internet-Based Brief Intervention to Prevent Unhealthy Alcohol Use among Young Men: A Randomized Controlled Trial. *PLOS ONE*, *10*(12), e0144146. https://doi.org/10.1371/journal.pone.0144146

Bertholet, N., Schmutz, E., Studer, J., Adam, A., Gmel, G., Cunningham, J. A., McNeely, J., & Daeppen, J.-B. (2023). Effect of a smartphone intervention as a secondary prevention for use among university students with unhealthy alcohol use: Randomised controlled trial. *BMJ*, 1–11. https://doi.org/10.1136/bmj-2022-073713

Bertholet, N., Studer, J., Cunningham, J. A., Gmel, G., Burnand, B., & Daeppen, J. (2018). Four‐year follow‐up of an internet‐based brief intervention for unhealthy alcohol use in young men. *Addiction*, *113*(8), 1517–1521. https://doi.org/10.1111/add.14179

Bewick, B. M., Trusler, K., Mulhern, B., Barkham, M., & Hill, A. J. (2008). The feasibility and effectiveness of a web-based personalised feedback and social norms alcohol intervention in UK university students: A randomised control trial. *Addictive Behaviors*, *33*(9), 1192–1198. https://doi.org/10.1016/j.addbeh.2008.05.002

Bewick, B. M., West, R., Gill, J., O’May, F., Mulhern, B., Barkham, M., & Hill, A. J. (2010). Providing Web-Based Feedback and Social Norms Information to Reduce Student Alcohol Intake: A Multisite Investigation. *Journal of Medical Internet Research*, *12*(5), e59. https://doi.org/10.2196/jmir.1461

Bewick, B. M., West, R. M., Barkham, M., Mulhern, B., Marlow, R., Traviss, G., & Hill, A. J. (2013). The Effectiveness of a Web-Based Personalized Feedback and Social Norms Alcohol Intervention on United Kingdom University Students: Randomized Controlled Trial. *Journal of Medical Internet Research*, *15*(7), e137. https://doi.org/10.2196/jmir.2581

Bonar, E. E., Cunningham, R. M., Sweezea, E. C., Blow, F. C., Drislane, L. E., & Walton, M. A. (2021). Piloting a brief intervention plus mobile boosters for drug use among emerging adults receiving emergency department care. *Drug and Alcohol Dependence*, *221*, 108625. https://doi.org/10.1016/j.drugalcdep.2021.108625

Bonar, E. E., Goldstick, J. E., Chapman, L., Bauermeister, J. A., Young, S. D., McAfee, J., & Walton, M. A. (2022). A social media intervention for cannabis use among emerging adults: Randomized controlled trial. *Drug and Alcohol Dependence*, *232*, 109345. https://doi.org/10.1016/j.drugalcdep.2022.109345

Bonar, E. E., Tan, C. Y., Fernandez, A. C., Goldstick, J. E., Chapman, L., Florimbio, A. R., & Walton, M. A. (2024). A social media intervention for high-intensity drinking among emerging adults: A pilot randomized controlled trial. *Alcohol and Alcoholism*, *59*(2), 1–12. https://doi.org/10.1093/alcalc/agae005

Borsari, B., Short, E. E., Mastroleo, N. R., Hustad, J. T. P., Tevyaw, T. O., Barnett, N. P., Kahler, C. W., & Monti, P. M. (2014). Phone-delivered brief motivational interventions for mandated college students delivered during the summer months. *Journal of Substance Abuse Treatment*, *46*(5), 592–596. https://doi.org/10.1016/j.jsat.2014.01.001

Braitman, A. L., & Lau‐Barraco, C. (2018). Personalized Boosters After a Computerized Intervention Targeting College Drinking: A Randomized Controlled Trial. *Alcoholism: Clinical and Experimental Research*, *42*(9), 1735–1747. https://doi.org/10.1111/acer.13815

Bryant, Z. E. (2009). *Testing the Effectiveness of E-mailed Basics Feedback with College Students* [PhD Dissertation]. Auburn.

Buckner, J. D., Zvolensky, M. J., & Lewis, E. M. (2020). On-line personalized feedback intervention for negative affect and cannabis: A pilot randomized controlled trial. *Experimental and Clinical Psychopharmacology*, *28*(2), 143–149. https://doi.org/10.1037/pha0000304

Butler, L. H., & Correia, C. J. (2009). Brief alcohol intervention with college student drinkers: Face-to-face versus computerized feedback. *Psychology of Addictive Behaviors*, *23*(1), 163–167. https://doi.org/10.1037/a0014892

Cameron, D., Epton, T., Norman, P., Sheeran, P., Harris, P. R., Webb, T. L., Julious, S. A., Brennan, A., Thomas, C., Petroczi, A., Naughton, D., & Shah, I. (2015). A theory-based online health behaviour intervention for new university students (U@Uni:LifeGuide): Results from a repeat randomized controlled trial. *Trials*, *16*(1), 555. https://doi.org/10.1186/s13063-015-1092-4

Carey, K. B., Carey, M. P., Henson, J. M., Maisto, S. A., & DeMartini, K. S. (2011). Brief alcohol interventions for mandated college students: Comparison of face‐to‐face counseling and computer‐delivered interventions. *Addiction*, *106*(3), 528–537. https://doi.org/10.1111/j.1360-0443.2010.03193.x

Carey, K. B., DiBello, A. M., Magill, M., & Mastroleo, N. R. (2024). Does self-affirmation augment the effects of a mandated personalized feedback intervention? A randomized controlled trial with heavy drinking college students. *Psychology of Addictive Behaviors*, *38*(8), 836–849. https://doi.org/10.1037/adb0000989

Carey, K. B., Henson, J. M., Carey, M. P., & Maisto, S. A. (2009). Computer versus in-person intervention for students violating campus alcohol policy. *Journal of Consulting and Clinical Psychology*, *77*(1), 74–87. https://doi.org/10.1037/a0014281

Chavez, K., & Palfai, T. P. (2021). Reducing Heavy Episodic Drinking among College Students Using a Combined Web and Interactive Text Messaging Intervention. *Alcoholism Treatment Quarterly*, *39*(1), 82–95. https://doi.org/10.1080/07347324.2020.1784067

Chiauzzi, E., Green, T. C., Lord, S., Thum, C., & Goldstein, M. (2005). My Student Body: A High-Risk Drinking Prevention Web Site for College Students. *Journal of American College Health*, *53*(6), 263–274. https://doi.org/10.3200/JACH.53.6.263-274

Choi, M., Driver, M. N., Balcke, E., Saunders, T., Langberg, J. M., & Dick, D. M. (2023). Bridging the gap between genetic epidemiological research and prevention: A randomized control trial of a novel personalized feedback program for alcohol and cannabis use. *Drug and Alcohol Dependence*, *249*, 1–5. https://doi.org/10.1016/j.drugalcdep.2023.110818

Christoff, A. D. O., & Boerngen-Lacerda, R. (2015). Reducing substance involvement in college students: A three-arm parallel-group randomized controlled trial of a computer-based intervention. *Addictive Behaviors*, *45*, 164–171. https://doi.org/10.1016/j.addbeh.2015.01.019

Chun, J., Lee, H. K., Lee, J., & Lee, S. (2022). Effectiveness of web-based intervention for reducing problematic alcohol use in Korean female college students. *Journal of Substance Use*, *27*(1), 20–26. https://doi.org/10.1080/14659891.2021.1884297

Collins, S. E., Kirouac, M., Lewis, M. A., Witkiewitz, K., & Carey, K. B. (2014). Randomized Controlled Trial of Web-Based Decisional Balance Feedback and Personalized Normative Feedback for College Drinkers. *Journal of Studies on Alcohol and Drugs*, *75*(6), 982–992. https://doi.org/10.15288/jsad.2014.75.982

Conner, B. T., Thompson, K., Prince, M. A., Bolts, O. L., Contreras, A., Riggs, N. R., & Leadbeater, B. J. (2024). Results of a randomized controlled trial of the cannabis eCHECKUP TO GO personalized normative feedback intervention on reducing cannabis use, cannabis consequences, and descriptive norms. *Journal of Substance Use and Addiction Treatment*, *159*, 1–8. https://doi.org/10.1016/j.josat.2023.209267

Côté, J., Tessier, S., Gagnon, H., April, N., Rouleau, G., & Chagnon, M. (2018). Efficacy of a Web-Based Tailored Intervention to Reduce Cannabis Use Among Young People Attending Adult Education Centers in Quebec. *Telemedicine and E-Health*, *24*(11), 853–860. https://doi.org/10.1089/tmj.2017.0144

Croom, K., Lewis, D., Marchell, T., Lesser, M. L., Reyna, V. F., Kubicki-Bedford, L., Feffer, M., & Staiano-Coico, L. (2009). Impact of an Online Alcohol Education Course on Behavior and Harm for Incoming First-Year College Students: Short-Term Evaluation of a Randomized Trial. *Journal of American College Health*, *57*(4), 445–454. https://doi.org/10.3200/JACH.57.4.445-454

Croom, K., Staiano-Coico, L., Lesser, M. L., Lewis, D. K., Reyna, V. F., Marchell, T. C., Frank, J., & Ives, S. (2015). The Glass Is Half Full: Evidence for Efficacy of Alcohol-Wise at One University But Not the Other. *Journal of Health Communication*, *20*(6), 627–638. https://doi.org/10.1080/10810730.2015.1012239

Crotwell, S. (2016). *Incorporating Alternative Sources of Reinforcement through Online CRA Goal Setting and the Effect on Substance Use in College Students* [PhD Dissertation]. University of New Mexico.

Donohue, B., Allen, D. N., Maurer, A., Ozols, J., & DeStefano, G. (2004). A Controlled Evaluation of Two Prevention Programs in Reducing Alcohol Use Among College Students at Low and High Risk for Alcohol Related Problems. *Journal of Alcohol and Drug Education*, *48*(1), 13–33.

Donovan, E., Das Mahapatra, P., Green, T. C., Chiauzzi, E., McHugh, K., & Hemm, A. (2015). Efficacy of an online intervention to reduce alcohol-related risks among community college students. *Addiction Research & Theory*, *23*(5), 437–447. https://doi.org/10.3109/16066359.2015.1043625

Donovan, E., Wood, M., Frayjo, K., Black, R. A., & Surette, D. A. (2012). A randomized, controlled trial to test the efficacy of an online, parent-based intervention for reducing the risks associated with college-student alcohol use. *Addictive Behaviors*, *37*(1), 25–35. https://doi.org/10.1016/j.addbeh.2011.09.007

Doumas, D. M., & Hannah, E. (2008). Preventing high-risk drinking in youth in the workplace: A web-based normative feedback program. *Journal of Substance Abuse Treatment*, *34*(3), 263–271. https://doi.org/10.1016/j.jsat.2007.04.006

Doumas, D. M., Kane, C. M., Navarro, T. B., & Roman, J. (2011). Decreasing Heavy Drinking in First‐Year Students: Evaluation of a Web‐Based Personalized Feedback Program Administered During Orientation. *Journal of College Counseling*, *14*(1), 5–20. https://doi.org/10.1002/j.2161-1882.2011.tb00060.x

Doumas, D. M., Nelson, K., DeYoung, A., & Renteria, C. C. (2014). Alcohol‐Related Consequences Among First‐Year University Students: Effectiveness of a Web‐Based Personalized Feedback Program. *Journal of College Counseling*, *17*(2), 150–162. https://doi.org/10.1002/j.2161-1882.2014.00054.x

Doumas, M., Rapp, M. A., & Krampe, R. Th. (2009). Working Memory and Postural Control: Adult Age Differences in Potential for Improvement, Task Priority, and Dual Tasking. *The Journals of Gerontology Series B: Psychological Sciences and Social Sciences*, *64B*(2), 193–201. https://doi.org/10.1093/geronb/gbp009

Dunn, M. E., Fried-Somerstein, A., Flori, J. N., Hall, T. V., & Dvorak, R. D. (2020). Reducing alcohol use in mandated college students: A comparison of a Brief Motivational Intervention (BMI) and the Expectancy Challenge Alcohol Literacy Curriculum (ECALC). *Experimental and Clinical Psychopharmacology*, *28*(1), 87–98. https://doi.org/10.1037/pha0000290

Edwards, S. M., Tuliao, A. P., Kennedy, J. L. D., & McChargue, D. E. (2020). Weekend Text Messages Increase Protective Behavioral Strategies and Reduce Harm Among College Drinkers. *Journal of Technology in Behavioral Science*, *5*(4), 395–401. https://doi.org/10.1007/s41347-020-00149-4

Elliott, J. C. (2012). *Evaluation of a Web-based intervention for college marijuana use* [Syracuse University]. ProQuest.

Elliott, J. C., & Carey, K. B. (2012). Correcting Exaggerated Marijuana Use Norms Among College Abstainers: A Preliminary Test of a Preventive Intervention. *Journal of Studies on Alcohol and Drugs*, *73*(6), 976–980. https://doi.org/10.15288/jsad.2012.73.976

Epton, T., Norman, P., Dadzie, A.-S., Harris, P. R., Webb, T. L., Sheeran, P., Julious, S. A., Ciravegna, F., Brennan, A., Meier, P. S., Naughton, D., Petroczi, A., Kruger, J., & Shah, I. (2014). A theory-based online health behaviour intervention for new university students (U@Uni): Results from a randomised controlled trial. *BMC Public Health*, *14*(1), 563. https://doi.org/10.1186/1471-2458-14-563

Fearnow-Kenney, M., Wyrick, D. L., Milroy, J. J., Reifsteck, E. J., Day, T., & Kelly, S. E. (2016). The Effects of a Web-Based Alcohol Prevention Program on Social Norms, Expectancies, and Intentions to Prevent Harm among College Student-Athletes. *The Sport Psychologist*, *30*(2), 113–122. https://doi.org/10.1123/tsp.2015-0016

Gajecki, M., Andersson, C., Rosendahl, I., Sinadinovic, K., Fredriksson, M., & Berman, A. H. (2017). Skills Training via Smartphone App for University Students with Excessive Alcohol Consumption: A Randomized Controlled Trial. *International Journal of Behavioral Medicine*, *24*(5), 778–788. https://doi.org/10.1007/s12529-016-9629-9

Gajecki, M., Berman, A. H., Sinadinovic, K., Rosendahl, I., & Andersson, C. (2014). Mobile phone brief intervention applications for risky alcohol use among university students: A randomized controlled study. *Clinical Practice*.

Ganz, T., Braun, M., Laging, M., Schermelleh-Engel, K., Michalak, J., & Heidenreich, T. (2018). Effects of a stand-alone web-based electronic screening and brief intervention targeting alcohol use in university students of legal drinking age: A randomized controlled trial. *Addictive Behaviors*, *77*, 81–88. https://doi.org/10.1016/j.addbeh.2017.09.017

Geisner, I. M., Varvil-Weld, L., Mittmann, A. J., Mallett, K., & Turrisi, R. (2015). Brief web-based intervention for college students with comorbid risky alcohol use and depressed mood: Does it work and for whom? *Addictive Behaviors*, *42*, 36–43. https://doi.org/10.1016/j.addbeh.2014.10.030

Gilmore, A. K., Lewis, M. A., & George, W. H. (2015). A randomized controlled trial targeting alcohol use and sexual assault risk among college women at high risk for victimization. *Behaviour Research and Therapy*, *74*, 38–49. https://doi.org/10.1016/j.brat.2015.08.007

Gonzales, R., Hernandez, M., Murphy, D. A., & Ang, A. (2016). Youth recovery outcomes at 6 and 9 months following participation in a mobile texting recovery support aftercare pilot study: Follow-Up of a Mobile Texting Aftercare Study. *The American Journal on Addictions*, *25*(1), 62–68. https://doi.org/10.1111/ajad.12322

Goodness, T. M. (2019). *Electronic Screening and Brief Intervention to Reduce Marijuana Use and Consequences Among Graduate Students Presenting to a Student Health Center: A Pilot Study* [Ph.D., Boston University]. https://www.proquest.com/docview/2384819280/abstract/F5916AC097CD48ABPQ/1

Haug, S., Boumparis, N., Wenger, A., Schaub, M. P., & Paz Castro, R. (2022). Efficacy of a Mobile App-Based Coaching Program for Addiction Prevention among Apprentices: A Cluster-Randomized Controlled Trial. *International Journal of Environmental Research and Public Health*, *19*(23), 1–12. https://doi.org/10.3390/ijerph192315730

Hester, R. K., Delaney, H. D., & Campbell, W. (2012). The College Drinker’s Check-Up: Outcomes of two randomized clinical trials of a computer-delivered intervention. *Psychology of Addictive Behaviors*, *26*(1), 1–12. https://doi.org/10.1037/a0024753

Hides, L., Quinn, C., Cockshaw, W., Stoyanov, S., Zelenko, O., Johnson, D., Tjondronegoro, D., Quek, L.-H., & Kavanagh, D. J. (2018). Efficacy and outcomes of a mobile app targeting alcohol use in young people. *Addictive Behaviors*, *77*, 89–95. https://doi.org/10.1016/j.addbeh.2017.09.020

Hogan, L. M. (2005). *Devedloping and Evaluating Brief, Computerised Interventions for Excessive Drinkers* [PhD Dissertation, University of Wales, Bangor]. https://regroup-production.s3.amazonaws.com/documents/ReviewReference/408889960/hogan%202005.pdf?response-content-type=application%2Fpdf&X-Amz-Algorithm=AWS4-HMAC-SHA256&X-Amz-Credential=AKIAYSFKCAWYQ4D5IUHG%2F20240214%2Fus-east-1%2Fs3%2Faws4_request&X-Amz-Date=20240214T201715Z&X-Amz-Expires=604800&X-Amz-SignedHeaders=host&X-Amz-Signature=7e6446245a0af88f9110b00b0a7e3a8446079bfae0b5c918d7eea1f48e340bc5

Hogan, L. M., & Cox, W. M. (2024). Evaluating Two Brief Motivational Interventions for Excessive-Drinking University Students. *Behavioral Sciences*, *14*(5), 1–13. https://doi.org/10.3390/bs14050381

Hustad, J. T. P., Barnett, N. P., Borsari, B., & Jackson, K. M. (2010). Web-based alcohol prevention for incoming college students: A randomized controlled trial. *Addictive Behaviors*, *35*(3), 183–189. https://doi.org/10.1016/j.addbeh.2009.10.012

Ji, X., Tang, Y., Jing, L., Zhou, L., Wu, B., Deng, Y., Zhou, S., & Yang, Y. (2023). Effects of a virtual reality-based motivational reinforcement + desensitization intervention program on psychological craving and addiction memory in female MA-dependent young adults. *Frontiers in Psychiatry*, *14*, 1–12. https://doi.org/10.3389/fpsyt.2023.1114878

Kazemi, D. M., Borsari, B., Levine, M. J., Li, S., Shehab, M., Fang, F., & Norona, J. C. (2020). Effectiveness of a Theory-Based mHealth Intervention for High-Risk Drinking in College Students. *Substance Use & Misuse*, *55*(10), 1667–1676. https://doi.org/10.1080/10826084.2020.1756851

Koelen, J., Klein, A., Wolters, N., Bol, E., De Koning, L., Roetink, S., Van Blom, J., Boutin, B., Schaaf, J., Grasman, R., Van Der Heijde, C. M., Salemink, E., Riper, H., Karyotaki, E., Cuijpers, P., Schneider, S., Rapee, R., Vonk, P., & Wiers, R. (2024). Web-Based, Human-Guided, or Computer-Guided Transdiagnostic Cognitive Behavioral Therapy in University Students With Anxiety and Depression: Randomized Controlled Trial. *JMIR Mental Health*, *11*, 1–20. https://doi.org/10.2196/50503

Kofler, D. (2021). *Cannabis Use Motives in Emerging Adulthood: Using Daily Diary Method to Inform Intervention* [Ph.D. Thesis]. University of Toronto.

Kypri, K., & McAnally, H. M. (2005). Randomized controlled trial of a web-based primary care intervention for multiple health risk behaviors. *Preventive Medicine*, *41*(3–4), 761–766. https://doi.org/10.1016/j.ypmed.2005.07.010

Laliberte, B. V. (2017). *The Effect of a Computer-Delivered Brief Intervention on Heavy Alcohol Use: A Pilot Study* [Dissertation, Wayne State University]. https://regroup-production.s3.amazonaws.com/documents/ReviewReference/408891277/Laliberte%202018.pdf?response-content-type=application%2Fpdf&X-Amz-Algorithm=AWS4-HMAC-SHA256&X-Amz-Credential=AKIAYSFKCAWYQ4D5IUHG%2F20240214%2Fus-east-1%2Fs3%2Faws4_request&X-Amz-Date=20240214T201921Z&X-Amz-Expires=604800&X-Amz-SignedHeaders=host&X-Amz-Signature=2a823402f7e45bad6c49ba8bc035cfc12ac86582a7d508ba8ffe6aa42a336185

Lane, D. J., Lindemann, D. F., & Schmidt, J. A. (2012). A Comparison of Computer-Assisted and Self-Management Programs for Reducing Alcohol Use among Students in First Year Experience Courses. *Journal of Drug Education*, *42*(2), 119–135. https://doi.org/10.2190/DE.42.2.a

Leavens, E. L. S., Miller, M. B., Brett, E. I., Baraldi, A., & Leffingwell, T. R. (2020). Influencing college students’ normative perceptions of protective behavioral strategies: A pilot randomized trial. *Addictive Behaviors*, *104*, 106256. https://doi.org/10.1016/j.addbeh.2019.106256

Lee, C. M., Cadigan, J. M., Kilmer, J. R., Cronce, J. M., Suffoletto, B., Walter, T., Fleming, C. B., & Lewis, M. A. (2021). Brief Alcohol Screening and Intervention for Community College Students (BASICCS): Feasibility and preliminary efficacy of web-conferencing BASICCS and supporting automated text messages. *Psychology of Addictive Behaviors*, *35*(7), 840–851. https://doi.org/10.1037/adb0000745

Leeman, R. F., DeMartini, K. S., Gueorguieva, R., Nogueira, C., Corbin, W. R., Neighbors, C., & O’Malley, S. S. (2016). Randomized controlled trial of a very brief, multicomponent web-based alcohol intervention for undergraduates with a focus on protective behavioral strategies. *Journal of Consulting and Clinical Psychology*, *84*(11), 1008–1015. https://doi.org/10.1037/ccp0000132

Lewis, M. A., Patrick, M. E., Litt, D. M., Atkins, D. C., Kim, T., Blayney, J. A., Norris, J., George, W. H., & Larimer, M. E. (2014). Randomized controlled trial of a web-delivered personalized normative feedback intervention to reduce alcohol-related risky sexual behavior among college students. *Journal of Consulting and Clinical Psychology*, *82*(3), 429–440. https://doi.org/10.1037/a0035550

Lovecchio, C. P. (2008). *On-Line Alcohol Education: Impact On Knowledge, Attitudes, and Behaviors of First-Year College Students* [Dissertation, Villanova University]. https://regroup-production.s3.amazonaws.com/documents/ReviewReference/408891325/Lovecchio%202010.pdf?response-content-type=application%2Fpdf&X-Amz-Algorithm=AWS4-HMAC-SHA256&X-Amz-Credential=AKIAYSFKCAWYQ4D5IUHG%2F20240214%2Fus-east-1%2Fs3%2Faws4_request&X-Amz-Date=20240214T202244Z&X-Amz-Expires=604800&X-Amz-SignedHeaders=host&X-Amz-Signature=4fa935ec2716ea624ef0866738d0997ea4ad9fe6cc872b8f986339cb55a006f6

Lovecchio, C. P., Wyatt, T. M., & DeJong, W. (2010). Reductions in Drinking and Alcohol-Related Harms Reported by First-Year College Students Taking an Online Alcohol Education Course: A Randomized Trial. *Journal of Health Communication*, *15*(7), 805–819. https://doi.org/10.1080/10810730.2010.514032

Mason, M., Benotsch, E. G., Way, T., Kim, H., & Snipes, D. (2014). Text Messaging to Increase Readiness to Change Alcohol Use in College Students. *The Journal of Primary Prevention*, *35*(1), 47–52. https://doi.org/10.1007/s10935-013-0329-9

Mason, M. J., Zaharakis, N. M., Moore, M., Brown, A., Garcia, C., Seibers, A., & Stephens, C. (2018). Who responds best to text-delivered cannabis use disorder treatment? A randomized clinical trial with young adults. *Psychology of Addictive Behaviors*, *32*(7), 699–709. https://doi.org/10.1037/adb0000403

Maybery, D., Reupert, A., Bartholomew, C., Cuff, R., Duncan, Z., McAuliffe, C., McLean, L., Pettenuzzo, L., Swing, A., & Foster, K. (2022). An online intervention for 18–25‐year‐old youth whose parents have a mental illness and/or substance use disorder: A pilot randomized controlled trial. *Early Intervention in Psychiatry*, *16*(11), 1249–1258. https://doi.org/10.1111/eip.13274

McCambridge, J., Bendtsen, M., Karlsson, N., White, I. R., Nilsen, P., & Bendtsen, P. (2013). Alcohol assessment and feedback by email for university students: Main findings from a randomised controlled trial. *British Journal of Psychiatry*, *203*(5), 334–340. https://doi.org/10.1192/bjp.bp.113.128660

Miller, M. B. (2015). *COMPARATIVE EFFICACY OF ALTERNATE PERSONALIZED FEEDBACK INTERVENTIONS FOR COLLEGE ALCOHOL MISUSE* [Dissertation]. Oklahoma State University.

Miller, M. B., Leavens, E. L., Meier, E., Lombardi, N., & Leffingwell, T. R. (2016). Enhancing the efficacy of computerized feedback interventions for college alcohol misuse: An exploratory randomized trial. *Journal of Consulting and Clinical Psychology*, *84*(2), 122–133. https://doi.org/10.1037/ccp0000066

Moore, M. J., Soderquist, J., & Werch, C. (2005). Feasibility and Efficacy of a Binge Drinking Prevention Intervention for College Students Delivered via the Internet Versus Postal Mail. *Journal of American College Health*, *54*(1), 38–44. https://doi.org/10.3200/JACH.54.1.38-44

Moreira, M. T., Oskrochi, R., & Foxcroft, D. R. (2012). Personalised Normative Feedback for Preventing Alcohol Misuse in University Students: Solomon Three-Group Randomised Controlled Trial. *PLoS ONE*, *7*(9), e44120. https://doi.org/10.1371/journal.pone.0044120

Murphy, J. G., Dennhardt, A. A., Skidmore, J. R., Martens, M. P., & McDevitt-Murphy, M. E. (2010). Computerized versus motivational interviewing alcohol interventions: Impact on discrepancy, motivation, and drinking. *Psychology of Addictive Behaviors*, *24*(4), 628–639. https://doi.org/10.1037/a0021347

Murphy, J. G., Dennhardt, A. A., Yurasek, A. M., Skidmore, J. R., Martens, M. P., MacKillop, J., & McDevitt-Murphy, M. E. (2015). Behavioral economic predictors of brief alcohol intervention outcomes. *Journal of Consulting and Clinical Psychology*, *83*(6), 1033–1043. https://doi.org/10.1037/ccp0000032

Neighbors, C., Lee, C. M., Atkins, D. C., Lewis, M. A., Kaysen, D., Mittmann, A., Fossos, N., Geisner, I. M., Zheng, C., & Larimer, M. E. (2012). A randomized controlled trial of event-specific prevention strategies for reducing problematic drinking associated with 21st birthday celebrations. *Journal of Consulting and Clinical Psychology*, *80*(5), 850–862. https://doi.org/10.1037/a0029480

Neighbors, C., Lee, C. M., Lewis, M. A., Fossos, N., & Walter, T. (2009). Internet-based personalized feedback to reduce 21st-birthday drinking: A randomized controlled trial of an event-specific prevention intervention. *Journal of Consulting and Clinical Psychology*, *77*(1), 51–63. https://doi.org/10.1037/a0014386

Neighbors, C., Lewis, M. A., Bergstrom, R. L., & Larimer, M. E. (2006). Being controlled by normative influences: Self-determination as a moderator of a normative feedback alcohol intervention. *Health Psychology*, *25*(5), 571–579. https://doi.org/10.1037/0278-6133.25.5.571

Neville, F. G., Williams, D. J., Goodall, C. A., Murer, J. S., & Donnelly, P. D. (2013). An Experimental Trial Exploring the Impact of Continuous Transdermal Alcohol Monitoring upon Alcohol Consumption in a Cohort of Male Students. *PLoS ONE*, *8*(6), e67386. https://doi.org/10.1371/journal.pone.0067386

Palfai, T. P., Saitz, R., Winter, M., Brown, T. A., Kypri, K., Goodness, T. M., O’Brien, L. M., & Lu, J. (2014). Web-based screening and brief intervention for student marijuana use in a university health center: Pilot study to examine the implementation of eCHECKUP TO GO in different contexts. *Addictive Behaviors*, *39*(9), 1346–1352. https://doi.org/10.1016/j.addbeh.2014.04.025

Paschall, M. J., & Bersamin, M. (2006). Short-Term Evaluation of a Web-based College Alcohol Misuse and Harm Prevention Course (College Alc). *Journal of Alcohol and Drug Education*, *50*(3), 49–65.

Patrick, M. E., Sur, A., Arterberry, B., Peterson, S., Morrell, N., & Vock, D. M. (2023). Examining engagement effects in an adaptive preventive intervention for college student drinking. *Journal of Consulting and Clinical Psychology*, *91*(11), 652–664. https://doi.org/10.1037/ccp0000845

Pedersen, E. R., Neighbors, C., Atkins, D. C., Lee, C. M., & Larimer, M. E. (2017). Brief online interventions targeting risk and protective factors for increased and problematic alcohol use among American college students studying abroad. *Psychology of Addictive Behaviors*, *31*(2), 220–230. https://doi.org/10.1037/adb0000242

Pietsch, B., Arnaud, N., Lochbühler, K., Rossa, M., Kraus, L., Gomes De Matos, E., Grahlher, K., Thomasius, R., Hanewinkel, R., & Morgenstern, M. (2023). Effects of an App-Based Intervention Program to Reduce Substance Use, Gambling, and Digital Media Use in Adolescents and Young Adults: A Multicenter, Cluster-Randomized Controlled Trial in Vocational Schools in Germany. *International Journal of Environmental Research and Public Health*, *20*(3), 1–14. https://doi.org/10.3390/ijerph20031970

Pischke, C. R., Helmer, S. M., Pohlabeln, H., Muellmann, S., Schneider, S., Reintjes, R., Schmidt-Pokrzywniak, A., Girbig, M., Krämer, A., Icks, A., Walter, U., & Zeeb, H. (2021). Effects of a Brief Web-Based “Social Norms”-Intervention on Alcohol, Tobacco and Cannabis Use Among German University Students: Results of a Cluster-Controlled Trial Conducted at Eight Universities. *Frontiers in Public Health*, *9*, 659875. https://doi.org/10.3389/fpubh.2021.659875

Reavley, N. J., McCann, T. V., Cvetkovski, S., & Jorm, A. F. (2014). A multifaceted intervention to improve mental health literacy in students of a multicampus university: A cluster randomised trial. *Social Psychiatry and Psychiatric Epidemiology*, *49*(10), 1655–1666. https://doi.org/10.1007/s00127-014-0880-6

Rew, L., Slesnick, N., Johnson, K., & Sales, A. (2022). Promoting Healthy Attitudes and Behaviors in Youth Who Experience Homelessness: Results of a Longitudinal Intervention Study. *Journal of Adolescent Health*, *70*(6), 942–949. https://doi.org/10.1016/j.jadohealth.2021.12.025

Ridout, B., & Campbell, A. (2014). Using F acebook to deliver a social norm intervention to reduce problem drinking at university. *Drug and Alcohol Review*, *33*(6), 667–673. https://doi.org/10.1111/dar.12141

Riggs, N. R., Conner, B. T., Parnes, J. E., Prince, M. A., Shillington, A. M., & George, M. W. (2018). Marijuana eCHECKUPTO GO: Effects of a personalized feedback plus protective behavioral strategies intervention for heavy marijuana-using college students. *Drug and Alcohol Dependence*, *190*, 13–19. https://doi.org/10.1016/j.drugalcdep.2018.05.020

Riordan, B. C., Winter, T., Carey, K. B., Conner, T. S., Moradi, S., Jang, K., Reid, K. E., Mason, A., & Scarf, D. (2023). A combined web based intervention and ecological momentary intervention for reducing alcohol use among incoming first-year university students: Results from a three-arm randomised controlled trial. *Addictive Behaviors*, *136*, 107471. https://doi.org/10.1016/j.addbeh.2022.107471

Scharer, J. L. (2019). *Evaluation of a Brief Online Alcohol Intervention for College Students: The Role of Race-Specific Normative Feedback, Racial/Ethnic Identity, and Readiness to Change* [Dissertation, University of Missouri, St. Louis]. https://regroup-production.s3.amazonaws.com/documents/ReviewReference/408890438/Scharer%202019.pdf?response-content-type=application%2Fpdf&X-Amz-Algorithm=AWS4-HMAC-SHA256&X-Amz-Credential=AKIAYSFKCAWYQ4D5IUHG%2F20240214%2Fus-east-1%2Fs3%2Faws4_request&X-Amz-Date=20240214T202929Z&X-Amz-Expires=604800&X-Amz-SignedHeaders=host&X-Amz-Signature=4558005f9cb9fe6421a5ce55391d183ed5b5d9c3a6069d9ce3b4e275ca5fb7e4

Schuckit, M. A., Smith, T. L., Kalmijn, J., Skidmore, J., Clausen, P., Shafir, A., Saunders, G., Bystritsky, H., & Fromme, K. (2015). The Impact of Focusing a Program to Prevent Heavier Drinking on a Pre‐existing Phenotype, the Low Level of Response to Alcohol. *Alcoholism: Clinical and Experimental Research*, *39*(2), 308–316. https://doi.org/10.1111/acer.12620

Shuai, R., Ahmed-Leitao, F., Bloom, J., Seedat, S., & Hogarth, L. (2024). Brief online negative affect focused functional imagery training (FIT) improves four-week drinking outcomes in hazardous student drinkers: A pilot randomised controlled trial replication in South Africa. *Addictive Behaviors Reports*, *19*, 1–9. https://doi.org/10.1016/j.abrep.2024.100540

Shuai, R., Bakou, A. E., Andrade, J., Hides, L., & Hogarth, L. (2022). Brief Online Negative Affect Focused Functional Imagery Training Improves 2-Week Drinking Outcomes in Hazardous Student Drinkers: A Pilot Randomised Controlled Trial. *International Journal of Behavioral Medicine*, *29*(3), 346–356. https://doi.org/10.1007/s12529-021-10019-9

Stapinski, L. A., Prior, K., Newton, N. C., Biswas, R. K., Kelly, E., Deady, M., Lees, B., Teesson, M., & Baillie, A. J. (2021). Are we making Inroads? A randomized controlled trial of a psychologist-supported, web-based, cognitive behavioral therapy intervention to reduce anxiety and hazardous alcohol use among emerging adults. *EClinicalMedicine*, *39*, 101048. https://doi.org/10.1016/j.eclinm.2021.101048

Stappenbeck, C. A., Gulati, N. K., Jaffe, A. E., Blayney, J. A., & Kaysen, D. (2021). Initial efficacy of a web-based alcohol and emotion regulation intervention for college women with sexual assault histories. *Psychology of Addictive Behaviors*, *35*(7), 852–865. https://doi.org/10.1037/adb0000762

Strohman, A. S., Braje, S. E., Alhassoon, O. M., Shuttleworth, S., Van Slyke, J., & Gandy, S. (2016). Randomized controlled trial of computerized alcohol intervention for college students: Role of class level. *The American Journal of Drug and Alcohol Abuse*, *42*(1), 15–24. https://doi.org/10.3109/00952990.2015.1105241

Suffoletto, B., Callaway, C., Kristan, J., Kraemer, K., & Clark, D. B. (2012). Text‐Message‐Based Drinking Assessments and Brief Interventions for Young Adults Discharged from the Emergency Department. *Alcoholism: Clinical and Experimental Research*, *36*(3), 552–560. https://doi.org/10.1111/j.1530-0277.2011.01646.x

Suffoletto, B., Kristan, J., Chung, T., Jeong, K., Fabio, A., Monti, P., & Clark, D. B. (2015). An Interactive Text Message Intervention to Reduce Binge Drinking in Young Adults: A Randomized Controlled Trial with 9-Month Outcomes. *PLOS ONE*, *10*(11), e0142877. https://doi.org/10.1371/journal.pone.0142877

Tanner, A. E., Guastaferro, K. M., Rulison, K. L., Wyrick, D. L., Milroy, J. J., Bhandari, S., Thorpe, S., Ware, S., Miller, A. M., & Collins, L. M. (2021). A Hybrid Evaluation-Optimization Trial to Evaluate an Intervention Targeting the Intersection of Alcohol and Sex in College Students and Simultaneously Test an Additional Component Aimed at Preventing Sexual Violence. *Annals of Behavioral Medicine*, *55*(12), 1184–1187. https://doi.org/10.1093/abm/kaab003

Teeters, J. B., Armstrong, N. M., King, S. A., & Hubbard, S. M. (2022a). A randomized pilot trial of a mobile phone–based brief intervention with personalized feedback and interactive text messaging to reduce driving after cannabis use and riding with a cannabis impaired driver. *Journal of Substance Abuse Treatment*, *142*, 108867. https://doi.org/10.1016/j.jsat.2022.108867

Teeters, J. B., Armstrong, N. M., King, S. A., & Hubbard, S. M. (2022b). A randomized pilot trial of a mobile phone–based brief intervention with personalized feedback and interactive text messaging to reduce driving after cannabis use and riding with a cannabis impaired driver. *Journal of Substance Abuse Treatment*, *142*, 1–9. https://doi.org/10.1016/j.jsat.2022.108867

Thomas, K., Müssener, U., Linderoth, C., Karlsson, N., Bendtsen, P., & Bendtsen, M. (2018). Effectiveness of a Text Messaging–Based Intervention Targeting Alcohol Consumption Among University Students: Randomized Controlled Trial. *JMIR mHealth and uHealth*, *6*(6), e146. https://doi.org/10.2196/mhealth.9642

Thompson, K., Burgess, J., & MacNevin, P. D. (2018). An Evaluation of e-CHECKUP TO GO in Canada: The Mediating Role of Changes in Social Norm Misperceptions. *Substance Use & Misuse*, *53*(11), 1849–1858. https://doi.org/10.1080/10826084.2018.1441306

Thompson, R. G., Aivadyan, C., Stohl, M., Aharonovich, E., & Hasin, D. S. (2020). Smartphone application plus brief motivational intervention reduces substance use and sexual risk behaviors among homeless young adults: Results from a randomized controlled trial. *Psychology of Addictive Behaviors*, *34*(6), 641–649. https://doi.org/10.1037/adb0000570

Tossmann, Dr. H.-P., Jonas, B., Tensil, M.-D., Lang, P., & Strüber, E. (2011). A Controlled Trial of an Internet-Based Intervention Program for Cannabis Users. *Cyberpsychology, Behavior, and Social Networking*, *14*(11), 673–679. https://doi.org/10.1089/cyber.2010.0506

Towe, S., & Stephens, R. S. (2014). The impact of personalized feedback on marijuana use: Examining a brief intervention delivered via the Internet. *Drug and Alcohol Dependence*, *140*, e227. https://doi.org/10.1016/j.drugalcdep.2014.02.628

Tucker, J. S., Linnemayr, S., Pedersen, E. R., Shadel, W. G., Zutshi, R., DeYoreo, M., & Cabreros, I. (2021). Pilot Randomized Clinical Trial of a Text Messaging-Based Intervention for Smoking Cessation Among Young People Experiencing Homelessness. *Nicotine & Tobacco Research*, *23*(10), 1691–1698. https://doi.org/10.1093/ntr/ntab055

Voogt, C. V., Poelen, E. A. P., Kleinjan, M., Lemmers, L. A. C. J., & Engels, R. C. M. E. (2013). The Effectiveness of the ‘What Do You Drink’ Web-based Brief Alcohol Intervention in Reducing Heavy Drinking among Students: A Two-arm Parallel Group Randomized Controlled Trial. *Alcohol and Alcoholism*, *48*(3), 312–321. https://doi.org/10.1093/alcalc/ags133

Wagener, T. L., Leffingwell, T. R., Mignogna, J., Mignogna, M. R., Weaver, C. C., Cooney, N. J., & Claborn, K. R. (2012). Randomized trial comparing computer-delivered and face-to-face personalized feedback interventions for high-risk drinking among college students. *Journal of Substance Abuse Treatment*, *43*(2), 260–267. https://doi.org/10.1016/j.jsat.2011.11.001

Walters, S. T., Vader, A. M., & Harris, T. R. (2007). A Controlled Trial of Web-Based Feedback for Heavy Drinking College Students. *Prevention Science*, *8*(1), 83–88. https://doi.org/10.1007/s11121-006-0059-9

Walters, S. T., Vader, A. M., Harris, T. R., Field, C. A., & Jouriles, E. N. (2009). Dismantling motivational interviewing and feedback for college drinkers: A randomized clinical trial. *Journal of Consulting and Clinical Psychology*, *77*(1), 64–73. https://doi.org/10.1037/a0014472

Weaver, C. C., Leffingwell, T. R., Lombardi, N. J., Claborn, K. R., Miller, M. E., & Martens, M. P. (2014). A computer-based feedback only intervention with and without a moderation skills component. *Journal of Substance Abuse Treatment*, *46*(1), 22–28. https://doi.org/10.1016/j.jsat.2013.08.011

Witkiewitz, K., Desai, S. A., Bowen, S., Leigh, B. C., Kirouac, M., & Larimer, M. E. (2014). Development and evaluation of a mobile intervention for heavy drinking and smoking among college students. *Psychology of Addictive Behaviors*, *28*(3), 639–650. https://doi.org/10.1037/a0034747
