# Supplementary material for: Technology-Based Substance Use Interventions for Emerging Adults and College Students: A Systematic Review and Meta-Analysis
Source: Int J Ment Health Addict. 2024 Dec 26;24(1):603–22. doi: 10.1007/s11469-024-01433-7 (PMC12456661; doi:10.1007/s11469-024-01433-7)
Supplement: Supplementary file 5 — Supplementary file5 (PDF 118 KB) [file 11469_2024_1433_MOESM5_ESM.pdf]

Appendix E-Risk of bias results

D1	Randomisation process	+	Low risk
D2	Deviations from the intended interventions	!	Some concerns
D3	Missing outcome data	-	High risk
D4	Measurement of the outcome		
D5	Selection of the reported result		

Author	Year	D1	D2	D3	D4	D5	Overall
Alfonso	2013	+	+	+	+	+	+
Andersson	2015	+	+	-	+	!	-
Andrade	2024	+	+	+	+	+	+
Arazan	2021	!	+	+	+	+	+
Barnett	2007	+	+	+	+	+	+
Bedendo	2024	+	+	+	+	+	+
Bendtsen	2015	+	+	!	+	+	!
Bendtsen	2012	+	+	!	+	+	!
Bernstein	2015	+	+	!	+	+	!
Bertholet	2018	+	+	+	+	!	!
Bertholet	2015	+	+	+	+	!	!
Bertholet	2015	+	+	+	+	!	!
Bertholet	2018	+	+	+	+	+	+
Bewick	2023	+	+	+	+	+	+
Bewick	2008	!	+	-	+	+	-
Bewick	2010	+	+	-	+	!	-
Bonar	2013	+	!	!	+	+	!
Bonar	2021	+	+	+	+	!	!
Bonar	2022	+	+	+	!	+	!
Borsari	2024	!	+	-	+	!	-
Braitman	2014	+	+	+	-	!	-
Bryant	2018	+	+	+	+	+	+
Buckner	2010	+	+	+	+	!	!
Butler	2019	+	!	+	+	!	!
Cameron	2009	+	+	-	-	!	-

Carey	2015	+	+	+	+	+	+
Carey	2009	+	+	+	+	+	+
Carey	2011	+	+	+	+	+	+
Chavez	2024	+	+	+	+	!	!
Chiauzzi	2021	+	+	+	+	+	+
Choi	2005	+	!	+	+	+	!
Christoff	2023	+	+	+	+	!	!
Chun	2015	+	+	+	+	+	+
Collins	2022	+	+	+	+	+	+
Conner	2014	!	+	+	+	+	!
Cote	2024	+	+	-	+	!	-
Croom	2018	!	+	+	+	!	!
Croom- A	2009	+	+	+	+	+	+
Croom- B	2015	+	+	+	+	+	+
Crotwell	2015	!	+	+	+	+	!
Donohue	2017	+	+	-	+	+	-
Donovan	2004	+	+	-	+	+	-
Donovan	2012	+	+	+	+	+	+
Doumas	2015	+	+	+	+	+	+
Doumas	2008	+	+	+	+	!	!
Doumas	2009	+	+	+	+	!	!
Doumas	2011	+	+	-	+	!	-
Dunn	2014	+	+	+	+	!	+
Edwards	2020	+	!	+	+	+	!
Elliot	2020	+	+	-	+	+	!
Elliott	2012	+	+	+	+	+	+
Epton	2013	!	+	-	+	!	-
Fearnow-Ker	2014	+	+	-	!	!	-
Gajecki	2016	+	+	+	+	!	!

Gajecki	2014	+	+	-	-	!	-
Ganz	2017	+	+	+	+	!	!
Geisner	2018	+	-	+	-	+	-
Gilmore	2015	+	+	+	-	+	-
Gonzales	2015	!	+	+	+	!	!
Goodness	2016	+	+	+	+	+	+
Haug	2020	+	+	+	+	+	+
Hester-1	2022	+	+	+	+	+	+
Hester-2	2012	+	+	+	+	+	+
Hides	2012	+	+	+	+	!	!
Hogan	2018	+	+	+	+	!	!
Hogan	2019	+	+	+	+	+	+
Hustad	2023	!	+	+	+	+	!
Ji	2010	+	+	-	+	+	-
Kazemi-1	2023	+	+	+	+	!	!
Koelen	2024	+	+	+	+	+	+
Kofler	2022	+	+	-	+	+	-
Kypri	2005	+	-	+	+	!	-
LaLiberte	2018	+	+	+	+	+	+
Lane	2012	+	!	+	!	!	!
Leavens	2020	+	+	+	+	!	!
Lee	2021	+	+	+	+	!	!
Lee	2014	+	+	+	+	+	+
Leeman	2016	!	+	+	+	!	!
Lewis	2014	+	+	+	+	!	!
Lovecchio	2008	+	+	+	+	+	+
Lovecchio	2010	+	+	-	-	!	-
Mason	2014	!	-	+	-	!	-
Mason	2018	+	+	+	+	!	!

Maybery	2022	+	+	+	+	!	!
McCambridg	2013	+	+	-	+	!	-
Miller	2016	+	+	-	+	!	-
Miller	2017	+	+	-	!	!	-
Moore	2005	+	+	-	-	!	-
Moreira	2012	+	+	+	+	!	!
Murphy	2010	+	+	+	+	!	!
Murphy	2015	+	+	+	+	+	+
Neighbors	2006	+	+	+	-	!	-
Neighbors	2009	!	+	+	+	!	!
Neighbors	2012	+	+	+	+	+	+
Neville	2013	+	+	-	+	!	-
Palfai	2014	+	+	+	+	!	!
Paschall	2006	+	+	-	+	!	-
Patrick	2023	+	+	+	+	+	+
Pedersen	2017	+	+	-	-	!	-
Pietsch	2023	+	+	+	+	+	+
Pischke	2021	+	+	-	+	!	-
Reavley	2014	!	+	-	!	!	-
Rew	2022	+	+	+	+	!	!
Ridout	2014	+	!	+	+	!	!
Riggs	2018	+	!	-	!	!	-
Riordan	2023	+	+	+	+	!	!
Scharer	2019	+	+	+	+	+	+
Schuckit	2015	+	+	+	+	!	!
Shuai	2022	+	+	+	+	!	!
Shuai	2024	+	+	+	+	+	+
Stapinski	2021	+	+	+	+	!	!
Stappenbeck	2021	!	+	+	+	!	!

Strohman	2016	+	+	-	+	!	-
Suffoletto	2012	+	+	+	+	!	!
Suffoletto	2015	+	+	+	+	!	!
Tanner	2021	+	+	+	+	+	+
Teeters	2022	+	!	+	+	+	!
Teeters	2022	+	+	-	+	+	-
Thomas	2018	+	+	+	!	!	!
Thompson	2018	+	+	+	+	+	+
Thompson	2020	+	!	+	-	!	-
Tossman	2011	+	!	+	!	!	!
Towe	2014	+	+	+	+	+	+
Tucker	2020	+	+	+	+	!	!
Voogt	2013	+	!	+	+	+	!
Wagener	2012	+	+	+	-	!	-
Walters	2007	+	+	+	+	!	!
Walters	2009	+	+	+	+	+	+
Weaver	2014	+	+	+	+	!	!
Witkiewitz	2014	+	+	+	+	+	+
