# Supplementary material for: Technology-Based Substance Use Interventions for Emerging Adults and College Students: A Systematic Review and Meta-Analysis
Source: Int J Ment Health Addict. 2024 Dec 26;24(1):603–22. doi: 10.1007/s11469-024-01433-7 (PMC12456661; doi:10.1007/s11469-024-01433-7)
Supplement: Supplementary file 6 — Supplementary file6 (PDF 95 KB) [file 11469_2024_1433_MOESM6_ESM.pdf]

Appendix F- Individual effect sizes

Author	Year	Hedges'g	Variance
Alfonso	2013	0.000	0.021
Paschall	2006	-0.055	0.006
Paschall	2006	-0.103	0.006
Paschall	2006	-0.058	0.006
Paschall	2006	0.029	0.006
Lane	2012	-0.408	0.031
Leavens	2020	0.022	0.012
Leavens	2020	0.179	0.012
Leavens	2020	0.098	0.012
Leavens	2020	-0.007	0.012
Lee	2021	0.091	0.017
Lee	2021	0.352	0.018
Lee	2021	0.339	0.018
Lee	2021	0.551	0.019
Lee	2021	0.334	0.018
Lee	2021	0.434	0.018
Lee	2021	0.392	0.018
Lee	2021	0.876	0.023
Lee	2010	0.050	0.006
Lee	2010	-0.066	0.006
Lee	2010	-0.107	0.006
Lee	2014	0.621	0.029
Lee	2014	0.993	0.037
Lee	2014	0.273	0.026
Lee	2014	0.576	0.029
Lee	2014	0.505	0.028
Lee	2014	0.577	0.029
Lee	2014	0.511	0.028
Lee	2014	0.661	0.030
Lee	2014	0.293	0.011
Lee	2014	0.124	0.011
Lee	2014	0.662	0.013
Lee	2014	0.080	0.011
Lee	2014	0.000	0.011
Barnett	2007	0.270	0.009
Barnett	2007	0.034	0.009
Barnett	2007	0.306	0.009
Barnett	2007	0.057	0.009
Barnett	2007	-0.017	0.009
Barnett	2007	-0.158	0.009
Barnett	2007	0.000	0.009

Barnett	2007	0.000	0.009
Barnett	2007	0.138	0.018
Barnett	2007	-0.067	0.018
Barnett	2007	-0.701	0.125
Barnett	2007	0.010	0.086
Hogan	2019	0.469	0.070
Hogan	2019	0.351	0.067
Hogan	2019	0.323	0.138
Hogan	2019	0.207	0.065
Hogan	2019	0.750	0.110
Hogan	2019	0.699	0.075
Hogan	2019	0.613	0.102
Hogan	2019	0.690	0.074
Hogan	2019	-0.095	0.085
Hogan	2019	0.484	0.067
Scharer	2019	0.219	0.016
Scharer	2019	0.307	0.016
Scharer	2019	0.503	0.018
Scharer	2019	0.042	0.017
Scharer	2019	-0.008	0.017
Scharer	2019	0.130	0.017
Lovecchio	2010	-0.260	0.001
Lovecchio	2010	0.118	0.001
Lovecchio	2010	-0.064	0.001
Lovecchio	2010	-0.343	0.001
Lovecchio	2010	0.320	0.001
Lovecchio	2010	-0.167	0.001
Lovecchio	2010	0.098	0.001
Lovecchio	2010	0.218	0.001
Lovecchio	2010	0.226	0.001
Lovecchio	2010	-0.082	0.001
Lovecchio	2010	-0.318	0.154
Bryant	2010	0.102	0.010
Bryant	2010	0.175	0.011
Bryant	2010	0.082	0.010
Bryant	2010	0.150	0.011
Bryant	2010	0.105	0.010
Bryant	2010	0.092	0.010
Bryant	2010	0.186	0.011
Crotwell	2017	0.352	0.013
Donovan	2015	0.210	0.006
Donovan	2015	0.419	0.007

Donovan	2015	0.207	0.006
Donovan	2015	0.322	0.007
Donovan	2015	0.161	0.006
Donovan	2015	0.088	0.006
Donovan	2015	0.034	0.006
Donovan	2015	0.099	0.006
Donovan	2015	0.000	0.006
Donovan	2015	-0.183	0.006
Donovan	2015	0.067	0.006
Donovan	2015	0.133	0.006
Donovan	2015	0.233	0.006
Donovan	2015	0.344	0.007
Donovan	2015	0.633	0.008
Donovan	2015	0.062	0.006
Donovan	2015	0.094	0.006
Donovan	2015	0.269	0.006
Donovan	2015	0.285	0.007
Donovan	2015	-0.044	0.015
Donovan	2015	0.314	0.007
Doumas	2011	0.277	0.028
Doumas	2011	0.000	0.027
Doumas	2011	0.173	0.028
Doumas	2011	0.278	0.028
Doumas	2014	0.219	0.192
Doumas	2014	0.913	0.890
Doumas	2014	0.865	0.373
Doumas	2014	0.289	0.027
Doumas	2014	1.167	0.118
Elliott	2013	0.032	0.013
Elliott	2013	0.074	0.013
Elliott	2013	0.000	0.013
Elliott	2013	0.107	0.013
Elliott	2013	0.080	0.013
Elliott	2013	-0.096	0.013
arnow-Kenr	2016	-0.013	0.002
Goodness	2019	0.418	0.044
Goodness	2019	0.392	0.044
Kypri	2005	-0.107	0.049
LaLiberte	2018	0.285	0.021
LaLiberte	2018	-0.116	0.039
Lovecchio	2008	0.152	0.002
Lovecchio	2008	0.193	0.002

Lovecchio	2008	0.223	0.002
Lovecchio	2008	0.257	0.026
Lovecchio	2008	0.247	0.006
Miller	2009	0.497	0.029
Miller	2009	0.687	0.032
Miller	2009	0.515	0.029
Miller	2009	0.453	0.034
Miller	2009	0.853	0.042
Miller	2009	0.603	0.036
Miller	2009	0.306	0.031
Miller	2009	0.572	0.035
Miller	2009	0.633	0.036
Mason	2014	0.866	0.161
Mason	2014	0.311	0.120
Mason	2014	0.037	0.114
Mason	2014	0.312	0.120
Mason	2014	0.323	0.121
Neighbors	2009	0.904	0.010
Neighbors	2009	0.035	0.007
Neighbors	2009	0.102	0.014
Neighbors	2009	0.249	0.014
Neighbors	2009	0.099	0.007
Neighbors	2009	0.585	0.008
Neighbors	2009	1.229	0.012
Stappenbec	2021	0.732	0.013
Stappenbec	2021	0.762	0.013
Stappenbec	2021	0.886	0.014
Stappenbec	2021	0.581	0.012
Stappenbec	2021	0.681	0.012
Stappenbec	2021	0.775	0.013
Towe	2014	0.266	0.025
Towe	2014	0.246	0.025
Towe	2014	0.312	0.025
Towe	2014	1.283	0.044
Towe	2014	0.957	0.035
Towe	2014	0.291	0.025
Towe	2014	0.539	0.027
Towe	2014	0.818	0.032
Towe	2014	0.317	0.025
Towe	2014	0.404	0.026
Towe	2014	0.330	0.025
Towe	2014	-0.396	0.026

Towe	2014	0.208	0.024
Walters	2009	0.447	0.016
Walters	2009	0.604	0.017
Walters	2009	0.521	0.016
Walters	2009	0.729	0.019
Walters	2009	0.233	0.015
Walters	2009	0.420	0.016
lcCambridg	2013	0.045	0.001
lcCambridg	2013	0.050	0.001
lcCambridg	2013	0.026	0.001
lcCambridg	2013	0.058	0.001
lcCambridg	2013	0.082	0.001
Miller	2016	0.369	0.021
Miller	2016	0.624	0.023
Miller	2016	0.539	0.023
Moreira	2012	0.049	0.006
Moreira	2012	0.108	0.006
Moreira	2012	0.736	0.004
Moreira	2012	0.787	0.004
Moreira	2012	0.204	0.006
Moreira	2012	0.110	0.006
Moreira	2012	0.348	0.003
Moreira	2012	0.469	0.003
Moreira	2012	-0.213	0.003
Moreira	2012	-0.351	0.003
Murphy	2010	0.202	0.029
Murphy	2010	-0.025	0.028
Murphy	2015	0.318	0.023
Murphy	2015	0.318	0.023
Murphy	2015	0.317	0.029
Murphy	2015	0.086	0.028
Murphy	2015	0.055	0.028
Murphy	2015	0.101	0.028
Murphy	2015	0.317	0.029
Murphy	2015	0.086	0.028
Murphy	2015	0.055	0.028
Murphy	2015	0.101	0.028
Neighbors	2012	0.117	0.021
Neighbors	2012	0.187	0.021
Neighbors	2012	0.257	0.021
Neighbors	2012	0.050	0.021
Neighbors	2012	0.181	0.021

Neighbors	2012	0.244	0.021
Neighbors	2012	0.055	0.020
Neighbors	2012	0.064	0.020
Neighbors	2012	0.252	0.021
Neighbors	2012	0.170	0.021
Neighbors	2012	0.249	0.021
Neighbors	2012	0.205	0.021
Neighbors	2012	0.175	0.020
Neighbors	2012	0.265	0.021
Neighbors	2012	0.307	0.021
Neighbors	2006	0.336	0.010
Neighbors	2006	0.356	0.010
Palfai	2014	0.001	0.016
Palfai	2014	0.034	0.016
Palfai	2014	0.436	0.017
Palfai	2014	0.471	0.018
Bendsten	2015	0.088	0.004
Bendsten	2015	1.317	0.005
Bendsten	2015	0.026	0.004
Bendsten	2015	0.075	0.004
Bendsten	2015	1.425	0.005
Bendsten	2015	1.272	0.005
Bendsten	2015	0.016	0.010
Bendsten	2015	0.940	0.005
Bendsten	2015	0.134	0.004
Pedersen	2017	-0.315	0.012
Pedersen	2017	-0.203	0.012
Pedersen	2017	0.123	0.012
Pedersen	2017	0.140	0.012
Pedersen	2017	-0.590	0.014
Pedersen	2017	-0.589	0.014
Pedersen	2017	0.045	0.012
Pedersen	2017	0.154	0.012
Pedersen	2017	-0.428	0.013
Pedersen	2017	-0.416	0.013
Pedersen	2017	0.138	0.012
Pedersen	2017	0.094	0.012
Pischke	2021	0.082	0.025
Pischke	2021	0.157	0.008
Pischke	2021	0.308	0.026
Pischke	2021	0.331	0.144
Pischke	2021	-0.172	0.009

Pischke	2021	-0.067	0.149
Pischke	2021	0.122	0.053
Pischke	2021	0.059	0.008
Pischke	2021	-0.088	0.020
Ridout	2014	0.628	0.025
Ridout	2014	0.652	0.025
Ridout	2014	0.705	0.026
Ridout	2014	0.723	0.026
Riggs	2018	0.137	0.009
Riggs	2018	-0.054	0.009
Riggs	2018	0.310	0.010
Riggs	2018	0.455	0.010
Riggs	2018	0.266	0.009
Thomas	2018	-0.028	0.005
Thomas	2018	0.100	0.005
Thomas	2018	-0.037	0.005
Thomas	2018	0.132	0.036
Thomas	2018	0.056	0.037
Thomas	2018	-0.054	0.010
Thomas	2018	0.012	0.008
Thomas	2018	0.018	0.006
Thomas	2018	0.061	0.020
Bertholet	2015	0.041	0.002
Bertholet	2015	-0.037	0.002
Bertholet	2015	-0.039	0.002
Bertholet	2015	0.379	0.002
Bertholet	2015	0.173	0.005
Bertholet	2015	-0.145	0.005
Bertholet	2015	0.063	0.003
Bertholet	2015	0.207	0.003
Bertholet	2015	0.398	0.003
Bertholet	2015	0.367	0.003
Bertholet	2015	0.339	0.011
Bertholet	2015	0.516	0.011
Bertholet	2018	-0.057	0.003
Bertholet	2018	1.051	0.004
Bewick	2008	0.242	0.006
Bewick	2008	0.056	0.006
Bewick	2008	0.124	0.006
Bewick	2010	0.310	0.003
Bewick	2010	0.338	0.003
Bewick	2010	0.347	0.003

Bewick	2010	0.356	0.003
Bewick	2010	0.441	0.003
Bewick	2010	0.395	0.003
Bewick	2010	0.374	0.003
Bewick	2010	0.398	0.003
Bewick	2013	0.232	0.001
Bewick	2013	0.369	0.003
Bewick	2013	0.207	0.004
Bewick	2013	0.280	0.001
Bewick	2013	0.424	0.003
Bewick	2013	0.414	0.004
Bewick	2013	0.033	0.004
Bewick	2013	0.104	0.006
Bewick	2013	0.024	0.007
Reavley	2014	0.268	0.007
Reavley	2014	0.132	0.006
Stapinski	2021	0.724	0.020
Stapinski	2021	0.715	0.020
Stapinski	2021	0.843	0.022
Stapinski	2021	0.962	0.023
Stapinski	2021	1.143	0.026
Stapinski	2021	0.772	0.021
Stapinski	2021	0.787	0.021
Stapinski	2021	0.849	0.274
Stapinski	2021	0.994	0.326
Stapinski	2021	0.360	0.129
Stapinski	2021	0.469	0.129
Bonar	2022	0.255	0.013
Bonar	2022	0.265	0.013
Bonar	2022	0.068	0.013
Bonar	2022	0.271	0.014
Bonar	2022	0.263	0.013
Bonar	2022	0.360	0.014
Bonar	2022	0.159	0.013
Bonar	2022	0.309	0.014
Bonar	2022	0.113	0.013
Bonar	2022	0.207	0.013
Bonar	2022	0.057	0.013
Bonar	2022	0.270	0.014
Bonar	2022	0.222	0.013
Bonar	2022	0.304	0.014
Bonar	2022	0.262	0.013

Bonar	2022	0.251	0.013
Bonar	2022	0.226	0.013
Bonar	2022	0.150	0.013
Bonar	2022	0.011	0.013
Bonar	2022	0.010	0.013
Bonar	2022	0.135	0.013
Bonar	2022	0.098	0.013
Bonar	2022	0.148	0.013
Bonar	2022	0.071	0.013
Bonar	2022	0.079	0.013
Bonar	2022	0.280	0.014
Bonar	2022	0.000	0.013
Bonar	2022	0.287	0.014
Bonar	2022	-0.150	0.013
Bonar	2022	-0.092	0.013
Bonar	2022	0.027	0.017
Bonar	2022	0.179	0.017
Bonar	2022	-0.049	0.017
Bonar	2022	0.066	0.017
Bonar	2022	0.042	0.017
Bonar	2022	0.286	0.018
Stapinski	2021	0.724	0.020
Stapinski	2021	0.715	0.020
Stapinski	2021	0.843	0.022
Stapinski	2021	0.962	0.023
Stapinski	2021	1.143	0.026
Stapinski	2021	0.772	0.021
Stapinski	2021	0.787	0.021
Chun	2022	0.489	0.018
Chun	2022	0.774	0.023
Suffoletto	2012	0.752	0.082
Suffoletto	2012	1.116	0.105
Suffoletto	2015	0.224	0.005
Suffoletto	2015	0.281	0.005
Suffoletto	2015	0.286	0.005
Suffoletto	2015	0.194	0.005
Suffoletto	2015	0.181	0.005
Suffoletto	2015	0.143	0.005
Suffoletto	2015	-0.101	0.005
Suffoletto	2015	-0.104	0.005
Suffoletto	2015	-0.039	0.005
Suffoletto	2015	-0.038	0.005

Suffoletto	2015	-0.019	0.005
Suffoletto	2015	0.407	0.017
Suffoletto	2015	0.423	0.021
Suffoletto	2015	0.333	0.024
Suffoletto	2015	0.299	0.045
Suffoletto	2015	0.502	0.085
Suffoletto	2015	0.958	0.323
Suffoletto	2015	0.079	0.028
Suffoletto	2015	-0.146	0.037
Suffoletto	2015	-0.102	0.042
Suffoletto	2015	0.202	0.057
Suffoletto	2015	0.471	0.116
Suffoletto	2015	0.779	0.272
Kofler	2022	-0.320	0.063
Kofler	2022	0.128	0.062
Kofler	2022	0.233	0.062
Maybery	2022	-0.119	0.051
Maybery	2022	-0.088	0.051
Thompson	2018	-0.181	0.006
Thompson	2018	-0.007	0.006
Thompson	2018	0.019	0.006
Thompson	2018	0.191	0.006
Thompson	2018	-0.002	0.006
Thompson	2018	-0.004	0.006
Thompson	2018	0.396	0.006
Thompson	2018	0.550	0.007
Thompson	2018	0.435	0.007
Thompson	2018	0.422	0.007
Thompson	2020	0.392	0.052
Thompson	2020	0.260	0.050
Riordan	2023	-0.164	0.004
Riordan	2023	-0.175	0.004
Riordan	2023	-0.103	0.004
Riordan	2023	-0.172	0.004
Riordan	2023	0.050	0.004
Riordan	2023	-0.158	0.004
Riordan	2023	-0.065	0.004
Riordan	2023	-0.345	0.005
Riordan	2023	0.106	0.008
Riordan	2023	0.060	0.008
Riordan	2023	-0.016	0.008
Riordan	2023	0.074	0.008

Riordan	2023	0.082	0.008
Riordan	2023	0.020	0.008
Riordan	2023	0.071	0.008
Riordan	2023	-0.046	0.008
Riordan	2023	-0.013	0.008
Riordan	2023	0.082	0.008
Riordan	2023	0.193	0.008
Riordan	2023	0.046	0.008
Riordan	2023	0.022	0.008
Riordan	2023	-0.019	0.008
Tossmann	2011	0.125	0.001
Tossmann	2011	0.256	0.001
Tossmann	2011	0.327	0.001
Tossmann	2011	0.248	0.001
Tossmann	2011	0.466	0.001
Tossmann	2011	0.328	0.001
Shuai	2022	0.485	0.045
Teeters	2022	0.442	0.028
Teeters	2022	0.003	0.035
Teeters	2022	0.411	0.028
Teeters	2022	0.360	0.037
Teeters	2022	0.281	0.027
Teeters	2022	0.191	0.035
Teeters	2022	0.526	0.029
Teeters	2022	0.214	0.036
Edwards	2020	0.153	0.025
Edwards	2020	-0.080	0.025
Voogt	2013	-0.328	0.002
Voogt	2013	0.027	0.002
Voogt	2013	0.026	0.009
Voogt	2013	0.451	0.008
Voogt	2013	0.057	0.009
Voogt	2013	0.438	0.007
Wagener	2012	0.141	0.025
Wagener	2012	0.534	0.029
Wagener	2012	0.495	0.028
Wagener	2012	0.430	0.028
Walters	2007	0.562	0.022
Walters	2007	0.679	0.023
Walters	2007	1.065	0.029
Walters	2007	0.895	0.026
Walters	2007	0.165	0.019

Walters	2007	0.245	0.019
Walters	2007	0.738	0.024
Walters	2007	0.697	0.023
Weaver	2014	0.250	0.031
Weaver	2014	-0.068	0.031
Weaver	2014	0.343	0.032
Weaver	2014	0.332	0.032
Weaver	2014	0.597	0.036
Weaver	2014	0.473	0.034
Weaver	2014	0.478	0.039
Weaver	2014	0.300	0.036
Weaver	2014	0.606	0.041
Weaver	2014	0.471	0.039
Weaver	2014	0.443	0.038
Weaver	2014	0.497	0.039
Witkiewitz	2014	0.270	0.034
Witkiewitz	2014	0.270	0.034
Witkiewitz	2014	0.146	0.033
Witkiewitz	2014	0.146	0.033
Witkiewitz	2014	0.123	0.033
Witkiewitz	2014	0.124	0.033
Witkiewitz	2014	0.877	0.045
Witkiewitz	2014	0.877	0.045
Andersson	2015	0.375	0.004
Andersson	2015	0.281	0.004
Andersson	2015	0.230	0.004
Andersson	2015	0.200	0.004
Andersson	2015	0.285	0.004
Andersson	2015	0.173	0.004
Andersson	2015	0.175	0.004
Andersson	2015	0.250	0.004
Andersson	2015	0.152	0.004
Andersson	2015	0.325	0.004
Arazan	2021	0.126	0.008
Arazan	2021	-0.038	0.006
Arazan	2021	0.087	0.006
Arazan	2021	0.053	0.008
Arazan	2021	-0.010	0.012
Arazan	2021	0.031	0.012
Arazan	2021	0.359	0.013
Arazan	2021	0.349	0.013
Arazan	2021	0.039	0.019

Arazan	2021	0.159	0.034
Arazan	2021	0.093	0.037
Arazan	2021	0.105	0.041
Arazan	2021	0.327	0.042
Arazan	2021	0.436	0.046
Arazan	2021	-0.138	0.071
Arazan	2021	-0.029	0.074
Arazan	2021	0.101	0.080
Arazan	2021	0.101	0.080
Arazan	2021	0.565	0.095
Arazan	2021	-0.038	0.095
Arazan	2021	0.453	0.104
Arazan	2021	0.450	0.211
Borsari	2014	0.149	0.044
Borsari	2014	0.964	0.062
Borsari	2014	0.555	0.045
Borsari	2014	0.438	0.048
Bendsten	2012	0.000	0.003
Bendsten	2012	0.000	0.003
Bendsten	2012	0.000	0.003
Bendsten	2012	-0.075	0.004
Bendsten	2012	-0.032	0.004
Bendsten	2012	-0.094	0.003
Bendsten	2012	0.090	0.005
Bendsten	2012	-0.610	0.483
Braitman	2018	0.154	0.006
Braitman	2018	0.154	0.006
Braitman	2018	0.154	0.006
Braitman	2018	0.154	0.006
Braitman	2018	0.230	0.006
Braitman	2018	0.307	0.006
Braitman	2018	0.209	0.006
Braitman	2018	0.298	0.006
Braitman	2018	0.248	0.006
Braitman	2018	0.211	0.006
Braitman	2018	0.171	0.006
Braitman	2018	0.221	0.006
Braitman	2018	0.171	0.006
Braitman	2018	0.186	0.006
Braitman	2018	0.112	0.006
Braitman	2018	0.151	0.006
Braitman	2018	0.171	0.006

Braitman	2018	0.179	0.006
Braitman	2018	0.271	0.006
Braitman	2018	0.472	0.006
Braitman	2018	0.385	0.006
Braitman	2018	0.343	0.006
Braitman	2018	0.479	0.006
Braitman	2018	0.431	0.006
Braitman	2018	0.138	0.006
Braitman	2018	0.211	0.006
Braitman	2018	0.086	0.006
Braitman	2018	0.147	0.006
Braitman	2018	0.062	0.006
Braitman	2018	0.177	0.006
Braitman	2018	0.000	0.011
Braitman	2018	0.000	0.011
Braitman	2018	-0.074	0.011
Braitman	2018	0.000	0.011
Braitman	2018	0.074	0.011
Braitman	2018	0.077	0.006
Braitman	2018	0.000	0.006
Braitman	2018	0.077	0.006
Braitman	2018	0.077	0.006
Braitman	2018	0.089	0.006
Braitman	2018	0.230	0.006
Braitman	2018	0.285	0.006
Braitman	2018	0.392	0.006
Braitman	2018	0.374	0.006
Braitman	2018	0.171	0.006
Braitman	2018	0.219	0.006
Braitman	2018	0.188	0.006
Braitman	2018	0.292	0.006
Braitman	2018	0.230	0.006
Braitman	2018	0.234	0.006
Braitman	2018	0.182	0.006
Braitman	2018	0.286	0.006
Braitman	2018	0.316	0.006
Braitman	2018	0.390	0.006
Braitman	2018	0.425	0.006
Braitman	2018	0.465	0.006
Braitman	2018	0.326	0.006
Braitman	2018	0.442	0.006
Braitman	2018	0.580	0.007

Braitman	2018	0.324	0.006
Braitman	2018	0.268	0.006
Braitman	2018	0.270	0.006
Braitman	2018	0.162	0.006
Braitman	2018	0.221	0.006
Braitman	2018	0.260	0.006
Braitman	2018	-0.074	0.011
Braitman	2018	0.000	0.011
Braitman	2018	0.074	0.011
Braitman	2018	0.074	0.011
Buckner	2019	0.269	0.040
Buckner	2019	0.122	0.039
Buckner	2019	-0.006	0.039
Buckner	2019	0.530	0.044
Buckner	2019	0.468	0.043
Carey	2009	0.176	0.010
Carey	2009	-0.007	0.010
Carey	2009	-0.089	0.010
Carey	2009	0.216	0.010
Carey	2009	0.065	0.010
Carey	2009	0.197	0.010
Carey	2009	0.204	0.010
Carey	2009	0.008	0.010
Carey	2009	-0.005	0.010
Carey	2009	0.192	0.010
Carey	2009	-0.142	0.010
Carey	2009	-0.185	0.010
Carey	2009	0.294	0.011
Carey	2009	0.000	0.010
Carey	2009	0.189	0.010
Carey	2009	0.081	0.010
Carey	2009	-0.150	0.010
Carey	2009	-0.122	0.010
Cote	2018	0.299	0.009
Cote	2018	0.298	0.016
Cote	2018	0.100	0.020
Cote	2018	0.186	0.017
Butler	2009	-0.074	0.067
Butler	2009	0.224	0.068
Butler	2009	0.306	0.068
Butler	2009	-0.086	0.067
Butler	2009	0.760	0.075

Butler	2009	0.958	0.078
Butler	2009	1.053	0.080
Butler	2009	0.478	0.072
Doumas	2008	0.303	0.017
Doumas	2008	0.219	0.016
Doumas	2008	0.263	0.008
Doumas	2008	0.312	0.017
Doumas	2008	0.288	0.016
Doumas	2008	0.296	0.008
Doumas	2008	0.325	0.017
Doumas	2008	0.243	0.016
Doumas	2008	0.282	0.008
Elliot	2012	0.349	0.020
Cameron	2015	1.391	0.003
Cameron	2015	-0.378	0.002
Cameron	2015	-0.331	0.002
Cameron	2015	-0.272	0.002
Cameron	2015	-0.446	0.003
Cameron	2015	-0.461	0.003
Cameron	2015	-0.035	0.004
Cameron	2015	-0.608	0.019
Cameron	2015	-0.671	0.045
Epton	2014	-0.077	0.002
Epton	2014	-0.095	0.002
Epton	2014	-0.258	0.002
Epton	2014	-0.117	0.003
Epton	2014	0.178	0.029
Epton	2014	0.051	0.005
Epton	2014	0.037	0.005
Epton	2014	0.082	0.005
Epton	2014	-0.213	0.036
Epton	2014	-0.214	0.037
Epton	2014	0.000	0.007
Epton	2014	0.000	0.008
Carey	2011	0.095	0.006
Carey	2011	-0.086	0.010
Carey	2011	0.098	0.009
Carey	2011	0.095	0.006
Carey	2011	0.262	0.006
Carey	2011	0.199	0.006
Carey	2011	0.005	0.006
Carey	2011	0.262	0.006

Carey	2011	0.170	0.006
Carey	2011	-0.152	0.006
Carey	2011	-0.192	0.006
Carey	2011	0.170	0.006
Carey	2011	0.142	0.006
Carey	2011	-0.063	0.006
Carey	2011	-0.288	0.006
Carey	2011	0.142	0.006
Hides	2018	0.404	0.011
Hides	2018	0.462	0.011
Hides	2018	0.686	0.013
Hides	2018	0.490	0.012
Hides	2018	-0.112	0.010
Hides	2018	-0.010	0.010
Hides	2018	-0.050	0.010
Hides	2018	-0.032	0.010
Hides	2018	0.168	0.010
Hides	2018	0.234	0.011
Hides	2018	0.268	0.011
Hides	2018	0.249	0.011
Hides	2018	0.176	0.010
Hides	2018	0.191	0.011
Hides	2018	0.101	0.010
Hides	2018	0.102	0.010
Hides	2018	0.272	0.011
Hides	2018	0.353	0.011
Hides	2018	0.242	0.011
Hides	2018	0.241	0.011
Hides	2018	0.290	0.011
Hides	2018	0.367	0.011
Hides	2018	0.235	0.011
Hides	2018	0.287	0.011
Hides	2018	0.068	0.010
Alfonso	2013	-0.104	0.021
Alfonso	2013	-0.035	0.021
Alfonso	2013	0.456	0.023
Alfonso	2013	0.000	0.021
Alfonso	2013	-0.104	0.021
Alfonso	2013	-0.035	0.021
Alfonso	2013	0.456	0.023
Kazemi	2020	-0.003	0.016
Kazemi	2020	0.000	0.016

Kazemi	2020	0.354	0.017
Kazemi	2020	0.206	0.010
Kazemi	2020	0.243	0.010
Kazemi	2020	0.089	0.010
Chiauzzi	2005	0.504	0.011
Chiauzzi	2005	0.791	0.012
Chiauzzi	2005	0.224	0.019
Chiauzzi	2005	0.331	0.010
Chiauzzi	2005	0.470	0.011
Chiauzzi	2005	0.254	0.010
Chiauzzi	2005	0.381	0.010
Chiauzzi	2005	0.118	0.019
Chiauzzi	2005	0.087	0.009
Chiauzzi	2005	0.359	0.010
Chiauzzi	2005	0.361	0.010
Christoff	2015	0.313	0.005
Christoff	2015	0.313	0.005
Christoff	2015	0.337	0.004
Christoff	2015	0.337	0.004
Christoff	2015	1.387	0.080
Christoff	2015	0.209	0.061
Christoff	2015	0.208	0.060
Lewis	2014	0.503	0.009
Lewis	2014	0.516	0.009
Lewis	2014	0.440	0.009
Lewis	2014	0.457	0.009
Lewis	2014	0.549	0.010
Lewis	2014	0.539	0.010
Lewis	2014	0.263	0.009
Lewis	2014	0.297	0.009
Lewis	2014	-0.009	0.008
Lewis	2014	0.242	0.009
Lewis	2014	0.095	0.008
Lewis	2014	0.358	0.009
Lewis	2014	0.284	0.009
Lewis	2014	0.464	0.009
Lewis	2014	0.160	0.008
Lewis	2014	0.315	0.009
Lewis	2014	0.328	0.009
Lewis	2014	0.461	0.009
Lewis	2014	0.295	0.009
Lewis	2014	0.533	0.009

Lewis	2014	0.196	0.008
Lewis	2014	0.362	0.009
Lewis	2014	0.245	0.008
Lewis	2014	0.389	0.009
Lewis	2014	0.450	0.009
Lewis	2014	0.507	0.009
Lewis	2014	0.282	0.009
Lewis	2014	0.384	0.009
Lewis	2014	0.468	0.009
Lewis	2014	0.520	0.009
Lewis	2014	0.295	0.009
Lewis	2014	0.433	0.009
Lewis	2014	0.186	0.008
Lewis	2014	0.299	0.009
Lewis	2014	0.275	0.009
Lewis	2014	0.301	0.009
Collins	2014	0.109	0.004
Collins	2014	0.141	0.004
Collins	2014	0.181	0.005
Collins	2014	0.207	0.005
Collins	2014	0.293	0.005
Collins	2014	0.326	0.005
Collins	2014	0.148	0.004
Collins	2014	0.261	0.005
Collins	2014	0.314	0.005
Collins	2014	0.281	0.005
Collins	2014	0.192	0.005
Collins	2014	0.154	0.005
Collins	2014	0.221	0.005
Collins	2014	0.215	0.005
Collins	2014	0.219	0.005
Collins	2014	0.125	0.005
Collins	2014	0.021	0.005
Collins	2014	0.100	0.005
Moore	2005	-0.037	0.019
Moore	2005	-0.016	0.019
Moore	2005	0.000	0.019
Moore	2005	0.053	0.019
Moore	2005	0.124	0.019
Moore	2005	0.024	0.019
Moore	2005	0.020	0.019
Moore	2005	0.193	0.019

Neville	2013	0.921	0.106
Croom	2015	0.101	0.005
Croom	2015	0.180	0.005
Croom	2015	0.151	0.005
Croom	2015	0.100	0.005
Croom	2015	0.053	0.004
Croom	2015	0.090	0.004
Croom	2015	0.102	0.004
Croom	2015	0.111	0.004
Croom	2015	0.038	0.002
Croom	2015	0.052	0.002
Croom	2015	0.062	0.002
Croom	2015	0.099	0.002
Croom	2015	0.098	0.002
Croom	2015	0.062	0.006
Croom	2015	0.108	0.003
Croom	2015	0.213	0.005
Croom	2015	0.210	0.012
Croom	2015	-0.068	0.002
Croom	2015	-0.044	0.002
Croom	2015	0.049	0.002
Croom	2015	-0.053	0.002
Croom	2015	0.020	0.002
Croom	2015	-0.015	0.008
Croom	2015	0.026	0.003
Croom	2015	0.077	0.005
Croom	2015	0.032	0.012
Strohman	2016	0.327	0.067
Strohman	2016	0.710	0.080
Strohman	2016	0.736	0.081
Strohman	2016	0.493	0.071
Strohman	2016	0.450	0.070
Strohman	2016	0.000	0.067
Strohman	2016	-0.141	0.068
Strohman	2016	-0.204	0.069
Strohman	2016	-0.029	0.068
Strohman	2016	0.655	0.083
Tanner	2021	-0.044	0.029
Tanner	2021	-0.060	0.029
Donovan	2012	0.081	0.020
Donovan	2012	-0.034	0.020
Donovan	2012	-0.062	0.020

Dunn	2020	0.497	0.019
Dunn	2020	0.477	0.019
Dunn	2020	0.502	0.019
Dunn	2020	0.630	0.020
Dunn	2020	0.242	0.018
Dunn	2020	0.281	0.018
Dunn	2020	0.416	0.019
Dunn	2020	0.448	0.019
Berntein	2018	0.446	0.002
Bonar	2021	0.286	0.042
Bonar	2021	0.144	0.041
Bonar	2021	0.023	0.040
Bonar	2021	0.023	0.040
Bonar	2021	0.179	0.041
Bonar	2021	0.131	0.041
Bonar	2021	0.320	0.043
Bonar	2021	0.313	0.042
Doumas	2009	0.073	0.054
Doumas	2009	-0.192	0.055
Doumas	2009	0.058	0.054
Gonzales	2016	0.179	0.060
Gonzales	2016	0.164	0.060
Gonzales	2016	0.371	0.067
Gonzales	2016	0.448	0.066
Haug	2013	0.163	0.017
Haug	2013	0.330	0.005
Haug	2013	0.330	0.005
Haug	2013	0.078	0.029
Leeman	2016	0.292	0.021
Leeman	2016	0.183	0.021
Leeman	2016	0.282	0.021
Leeman	2016	0.223	0.021
Leeman	2016	0.298	0.019
Leeman	2016	0.282	0.019
Leeman	2016	0.406	0.022
Leeman	2016	0.271	0.021
Leeman	2016	0.021	0.020
Leeman	2016	0.247	0.021
Leeman	2016	0.278	0.021
Leeman	2016	0.044	0.021
Leeman	2016	0.234	0.021
Leeman	2016	0.385	0.022

Mason	2018	0.444	0.023
Mason	2018	0.516	0.024
Mason	2018	0.494	0.024
Mason	2018	0.082	0.021
Mason	2018	-0.339	0.022
Mason	2018	0.114	0.021
Mason	2018	0.505	0.024
Mason	2018	0.509	0.024
Mason	2018	0.382	0.022
Mason	2018	0.447	0.023
Mason	2018	0.641	0.025
Mason	2018	0.638	0.025
Mason	2018	0.286	0.022
Mason	2018	0.136	0.021
Mason	2018	0.265	0.022
Mason	2018	0.245	0.053
Mason	2018	1.205	0.090
Gajecki	2017	0.063	0.002
Gajecki	2017	0.016	0.002
Gajecki	2017	0.181	0.002
Gajecki	2017	0.000	0.002
Gajecki	2017	0.069	0.002
Gajecki	2017	0.067	0.002
Gajecki	2017	0.151	0.002
Gajecki	2017	0.059	0.002
Gajecki	2017	0.193	0.002
Gajecki	2017	0.000	0.002
Gajecki	2017	0.132	0.002
Gajecki	2017	0.114	0.002
Gajecki	2014	0.040	0.007
Gajecki	2014	0.000	0.007
Gajecki	2014	0.114	0.007
Gajecki	2014	0.000	0.007
Gajecki	2014	0.105	0.007
Gajecki	2014	-0.019	0.003
Gajecki	2014	-0.099	0.003
Gajecki	2014	0.070	0.003
Gajecki	2014	0.000	0.003
Gajecki	2014	-0.117	0.003
Ganz	2018	0.391	0.006
Ganz	2018	0.251	0.012
Ganz	2018	0.298	0.005

Ganz	2018	0.175	0.012
Ganz	2018	0.480	0.006
Ganz	2018	0.120	0.012
Ganz	2018	0.467	0.006
Ganz	2018	0.136	0.012
Geisner	2015	0.347	0.013
Geisner	2015	0.427	0.014
Geisner	2015	0.207	0.013
Geisner	2015	0.515	0.015
Bonar	2022	0.255	0.013
Bonar	2022	0.265	0.013
Bonar	2022	0.068	0.013
Bonar	2022	0.271	0.014
Bonar	2022	0.263	0.013
Bonar	2022	0.360	0.014
Bonar	2022	0.159	0.013
Bonar	2022	0.309	0.014
Bonar	2022	0.113	0.013
Bonar	2022	0.207	0.013
Bonar	2022	0.057	0.013
Bonar	2022	0.270	0.014
Bonar	2022	0.222	0.013
Bonar	2022	0.304	0.014
Bonar	2022	0.262	0.013
Bonar	2022	0.251	0.013
Bonar	2022	0.226	0.013
Bonar	2022	0.150	0.013
Bonar	2022	0.011	0.013
Bonar	2022	0.010	0.013
Bonar	2022	0.135	0.013
Bonar	2022	0.098	0.013
Bonar	2022	0.148	0.013
Bonar	2022	0.071	0.013
Bonar	2022	0.079	0.013
Bonar	2022	0.280	0.014
Bonar	2022	0.000	0.013
Bonar	2022	0.287	0.014
Bonar	2022	-0.150	0.013
Bonar	2022	-0.092	0.013
Bonar	2022	0.027	0.017
Bonar	2022	0.179	0.017
Bonar	2022	-0.049	0.017

Bonar	2022	0.066	0.017
Bonar	2022	0.042	0.017
Bonar	2022	0.286	0.018
Gilmore	2015	0.536	0.021
Gilmore	2015	0.186	0.019
Schuckit	2015	0.253	0.011
Schuckit	2015	0.360	0.012
Schuckit	2015	0.336	0.012
Schuckit	2015	0.387	0.012
Schuckit	2015	0.233	0.011
Schuckit	2015	0.192	0.011
Schuckit	2015	0.175	0.011
Schuckit	2015	0.135	0.011
Schuckit	2015	0.171	0.011
Schuckit	2015	0.193	0.011
Schuckit	2015	0.079	0.011
Schuckit	2015	0.038	0.011
Schuckit	2015	0.122	0.011
Schuckit	2015	0.184	0.011
Schuckit	2015	0.241	0.011
Schuckit	2015	0.243	0.011
Schuckit	2015	0.208	0.010
Schuckit	2015	0.295	0.010
Schuckit	2015	0.176	0.009
Schuckit	2015	0.241	0.010
Schuckit	2015	0.195	0.010
Schuckit	2015	0.172	0.009
Schuckit	2015	0.081	0.009
Schuckit	2015	0.078	0.009
Schuckit	2015	0.147	0.009
Schuckit	2015	0.255	0.010
Schuckit	2015	0.121	0.009
Schuckit	2015	0.161	0.009
Schuckit	2015	0.312	0.010
Schuckit	2015	0.257	0.010
Schuckit	2015	0.160	0.009
Schuckit	2015	0.157	0.009
Tucker	2020	0.423	0.163
Tucker	2020	0.416	0.080
Tucker	2020	0.340	0.089
Tucker	2020	0.406	0.128
Tucker	2020	0.403	0.155

Tucker	2020	0.486	0.192
Tucker	2020	0.027	0.095
Tucker	2020	0.219	0.235
Chavez	2021	1.244	0.108
Chavez	2021	0.451	0.066
Hester	2012	0.418	0.018
Hester	2012	0.908	0.024
Hester	2012	0.717	0.021
Hester	2012	1.177	0.028
Hester	2012	0.754	0.022
Hester	2012	1.015	0.025
Hester	2012	0.660	0.020
Hester	2012	0.961	0.025
Hester	2012	0.322	0.025
Hester	2012	0.656	0.028
Hester	2012	0.662	0.029
Hester	2012	0.702	0.029
Croom	2009	-0.315	0.001
Croom	2009	-0.394	0.004
Croom	2009	-0.530	0.004
Croom	2009	-0.369	0.003
Croom	2009	-0.445	0.004
Croom	2009	-0.388	0.018
Croom	2009	-0.673	0.006
Croom	2009	-0.362	0.004
Croom	2009	-0.645	0.009
Croom	2009	-0.340	0.006
Croom	2009	-0.468	0.007
Croom	2009	-0.512	0.010
Croom	2009	-0.287	0.006
Croom	2009	-0.916	0.033
Croom	2009	-0.945	0.025
Donohue	2004	0.015	0.039
Donohue	2004	0.145	0.065
Donohue	2004	0.103	0.039
Donohue	2004	0.304	0.067
Donohue	2004	0.528	0.044
Donohue	2004	0.132	0.064
Hustad	2010	-0.094	0.033
Hustad	2010	-0.077	0.032
Hustad	2010	0.152	0.033
Hustad	2010	-0.232	0.033

Hustad	2010	0.000	0.032
Hustad	2010	0.000	0.032
Hustad	2010	0.076	0.032
Hustad	2010	0.058	0.037
Hustad	2010	0.009	0.037
Hustad	2010	0.152	0.038
Hustad	2010	0.177	0.038
Hustad	2010	0.000	0.037
Hustad	2010	0.309	0.039
Gonzales	2016	0.804	0.090
Epton	2014	-0.707	0.121
Epton	2014	-0.698	0.099
Donovan	2015	-0.084	0.015
Donovan	2015	0.112	0.015
Donovan	2015	0.168	0.015
Gonzales	2016	0.903	0.089
Andrade	2024	0.221	0.009
Andrade	2024	0.000	0.009
Andrade	2024	-0.084	0.009
Bedendo	2024	0.085	0.006
Bedendo	2024	0.101	0.006
Bedendo	2024	0.128	0.006
Bedendo	2024	0.182	0.006
Bedendo	2024	0.135	0.006
Bedendo	2024	0.113	0.006
Bedendo	2024	0.064	0.006
Bedendo	2024	0.124	0.006
Bedendo	2024	0.193	0.006
Bedendo	2024	0.075	0.006
Bedendo	2024	0.153	0.006
Bedendo	2024	0.133	0.006
Bedendo	2024	0.214	0.007
Bedendo	2024	0.132	0.007
Bedendo	2024	0.222	0.007
Bedendo	2024	0.282	0.007
Bedendo	2024	0.139	0.007
Bedendo	2024	0.258	0.007
Bedendo	2024	0.198	0.007
Bedendo	2024	0.121	0.007
Bedendo	2024	0.132	0.007
Bedendo	2024	0.195	0.007
Bedendo	2024	0.106	0.007
Bedendo	2024	0.172	0.007
Bertholet	2023	0.067	0.002
Bertholet	2023	0.078	0.002
Bertholet	2023	0.077	0.002
Bertholet	2023	0.079	0.002
Bertholet	2023	0.108	0.002

Bertholet	2023	0.055	0.002
Bertholet	2023	0.112	0.002
Bertholet	2023	0.075	0.002
Bertholet	2023	0.035	0.002
Bertholet	2023	0.029	0.002
Bertholet	2023	-0.012	0.002
Bertholet	2023	0.069	0.002
Bertholet	2023	0.000	0.002
Bertholet	2023	0.027	0.002
Bertholet	2023	0.000	0.002
Bonar	2024	-0.090	0.044
Bonar	2024	-0.076	0.044
Bonar	2024	0.000	0.044
Bonar	2024	0.194	0.044
Bonar	2024	0.032	0.044
Bonar	2024	0.051	0.044
Bonar	2024	-0.281	0.045
Bonar	2024	0.000	0.044
Bonar	2024	0.161	0.044
Bonar	2024	0.235	0.044
Bonar	2024	-0.210	0.044
Bonar	2024	0.173	0.044
Bonar	2024	0.032	0.044
Bonar	2024	0.168	0.044
Bonar	2024	0.062	0.044
Bonar	2024	0.341	0.045
Bonar	2024	0.227	0.044
Bonar	2024	0.204	0.044
Bonar	2024	0.000	0.044
Bonar	2024	0.325	0.045
Carey	2024	0.175	0.012
Carey	2024	0.056	0.012
Carey	2024	-0.125	0.013
Carey	2024	0.069	0.013
Carey	2024	-0.139	0.013
Carey	2024	0.096	0.012
Carey	2024	-0.052	0.012
Carey	2024	-0.156	0.013
Carey	2024	0.066	0.013
Carey	2024	-0.142	0.013
Carey	2024	0.014	0.012
Carey	2024	-0.063	0.012
Carey	2024	-0.133	0.013
Carey	2024	-0.026	0.013
Carey	2024	-0.119	0.013
Carey	2024	-0.134	0.012
Carey	2024	-0.031	0.012
Carey	2024	0.000	0.013
Carey	2024	0.051	0.013
Carey	2024	-0.217	0.013
Choi	2023	-0.122	0.046
Choi	2023	-0.343	0.046

Choi	2023	-0.192	0.046
Choi	2023	0.000	0.046
Conner	2024	-0.193	0.016
Conner	2024	-0.126	0.024
Conner	2024	0.021	0.017
Haug	2022	0.169	0.003
Haug	2022	0.035	0.003
Hogan	2023	0.396	0.075
Hogan	2023	0.460	0.075
Hogan	2023	0.118	0.073
Hogan	2023	0.479	0.075
Hogan	2023	0.071	0.073
Hogan	2023	0.078	0.073
Hogan	2023	1.045	0.084
Hogan	2023	-0.175	0.074
Hogan	2023	0.894	0.081
Hogan	2023	-0.074	0.073
Hogan	2023	0.099	0.073
Hogan	2023	-0.323	0.074
Patrick	2023	0.127	0.005
Patrick	2023	0.000	0.005
Pietsch	2023	-0.062	0.002
Pietsch	2023	-0.042	0.002
Shuai	2024	0.321	0.079
Shuai	2024	-0.076	0.078
Shuai	2024	0.202	0.078
Shuai	2024	0.394	0.079
Shuai	2024	-0.300	0.078
Shuai	2024	0.071	0.078
Shuai	2024	0.673	0.082
Shuai	2024	0.757	0.083
Shuai	2024	0.489	0.080
Shuai	2024	0.305	0.078
Teeters	2022	0.309	0.058
Teeters	2022	0.137	0.057
Teeters	2022	0.501	0.059
Teeters	2022	0.338	0.058
Koelen	2024	0.663	0.020
Koelen	2024	-0.456	0.018
Koelen	2024	-0.349	0.016
Koelen	2024	0.665	0.017
Koelen	2024	1.107	0.022
Koelen	2024	0.443	0.018
Koelen	2024	0.221	0.018
Koelen	2024	-0.524	0.021
